# Costs and cost-effectiveness of treatment setting for children with wasting, oedema and growth failure/faltering: A systematic review

**DOI:** 10.1371/journal.pgph.0002551

**Published:** 2023-11-08

**Authors:** Noreen Dadirai Mdege, Sithabiso D. Masuku, Nozipho Musakwa, Mphatso Chisala, Ernest Ngeh Tingum, Micheal Kofi Boachie, Farhad Shokraneh

**Affiliations:** 1 Department of Health Sciences, University of York, York, United Kingdom; 2 Centre for Research in Health and Development, York, United Kingdom; 3 Health Economics and Epidemiology Research Office, Faculty of Health Sciences, University of the Witwatersrand, Johannesburg, South Africa; 4 Department of Population, Policy and Practice, Great Ormond Street Hospital, Institute of Child Health, University College London, London, United Kingdom; 5 Department of Economics, University of Namibia, Windhoek, Namibia; 6 Discipline of Public Health Medicine, School of Nursing and Public Health, University of KwaZulu-Natal, Durban, South Africa; 7 Department of Evidence Synthesis, Systematic Review Consultants LTD, Nottingham, United Kingdom; Indian Institute of Public Health Hyderabad, INDIA

## Abstract

This systematic review aimed to address the existing evidence gaps, and guide policy decisions on the settings within which to treat infants <12 months of age with growth faltering/failure, and infants and children aged <60 months with moderate wasting or severe wasting and/or bilateral pitting oedema. Twelve electronic databases were searched for studies published before 10 December 2021. The searches yielded 16,709 records from which 31 studies were eligible and included in the review. Three studies were judged as low quality, whilst 14 were moderate and the remaining 14 were high quality. We identified very few cost and cost-effectiveness analyses for most of the models of care with the certainty of evidence being judged at very low or low. However, there were 17 cost and 6 cost-effectiveness analyses for the initiation of treatment in outpatient settings for severe wasting and/or bilateral pitting oedema in infants and children <60 months of age. From this evidence, the costs appear lowest for initiating treatment in community settings, followed by initiating treatment in community and transferring to outpatient settings, initiating treatment in outpatients then transferring to community settings, initiating treatment in outpatient settings, and lastly initiating treatment in inpatient settings. In addition, the evidence suggested that initiation of treatment in outpatient settings is highly cost-effective when compared to doing nothing or no programme implementation scenarios, using country-specific WHO GDP per capita thresholds. The incremental cost-effectiveness ratios ranged from $20 to $145 per DALY averted from a provider perspective, and $68 to $161 per DALY averted from a societal perspective. However, the certainty of the evidence was judged as moderate because of comparisons to do nothing/ no programme scenarios which potentially limits the applicability of the evidence in real-world settings. There is therefore a need for evidence that compare the different available alternatives.

## Background

Child wasting, i.e., a child who is too thin for their height, can develop rapidly in the face of poor nutrient intake and/or disease [1, 2]. In 2020, about 6.7% (i.e., 45.4 million) of the world’s children under 5 years of age were affected by wasting, and 13.6 million were severely wasted [1]. The prevalence of wasting is highest in low- and middle-income countries (LMICs), with the majority of cases being in Sub-Saharan Africa and South Asia [3]. Moderately or severely wasted children have a weakened immunity, are susceptible to long-term developmental delays, and have a 5- to 20-fold increased risk of death [2–4]. Globally, each year, about 4.4% of deaths among children under 5 years of age are attributable to severe wasting [5]. Growth faltering/failure, on the other hand, describes lower weight or rate of weight gain, lower height, or an abnormally slow rate of gain in a child’s height or length, than expected for age and sex in childhood [6–8]. In LMICs the rates of early-life growth faltering/failure are unacceptably high due to poor health and social conditions [6, 7].

The current World Health Organization (WHO) guidelines for severe acute malnutrition (also referred to as severe wasting and oedema) in infants and children [2] have several gaps, including on recommendations for growth failure/faltering in infants under 6 months of age, the management of moderate wasting, and economic evidence to support decision making. Reviews of interventions for growth faltering/failure and child wasting in infants and young children have largely focused on the health and human impacts [9]. The few cost-effectiveness reviews that currently exist have focused on the different child undernutrition treatments, and the treatment of moderate or severe acute malnutrition at the community level [9, 10]. There is a glaring gap in reviews of cost-effectiveness evidence to guide policy decisions on the settings within which to treat this population.

We conducted this WHO-commissioned systematic review in order to complement the existing evidence and strengthen the WHO guidelines and recommendations. The review evaluates costs and cost-effectiveness of: initiation of treatment in a community setting; initiation of treatment in outpatient settings; referral to treatment from community to outpatient settings; referral to treatment in an inpatient setting; transfer from inpatient to outpatient/community treatment; transfer from outpatient to community settings; and discharge from outpatient/community treatment. The review focused on: 1) infants <12 months of age with growth faltering/failure; and 2) infants and children aged <60 months with moderate wasting or severe wasting and/or bilateral pitting oedema.

## Materials and methods

The systematic review was guided by well-established standardised principles and methods, including a pre-written protocol [11, 12]. The protocol was not registered, but was peer-reviewed by child nutrition, health economics and systematic review experts, and published [13]. The PRISMA Checklist is provided as S1 File.

### Inclusion/ Exclusion criteria

The inclusion and exclusion criteria are detailed in Table 1. Definitions used in this review for the population (i.e., moderate wasting, severe wasting and/or bilateral pitting oedema and growth failure/faltering), settings (i.e., community, outpatient and inpatient settings), and type of care (i.e., treatment initiation, referral, transfer or discharge) are provided in S2 File.

**Table 1:** Inclusion/ Exclusion criteria.

Selection Criteria	Inclusion	Exclusion
Population	Infants and children <5 years of age with moderate wasting or severe wasting and/or bilateral pitting oedema. Infants <12 months of age with growth failure/ faltering	For moderate and severe wasting and/or oedema Mixed populations that include the population of interest (i.e., infants and children <5 years of age with moderate or severe wasting and/or oedema) but where data for the population of interest is not reported separately. For growth failure/ faltering: Mixed populations that include the population of interest (i.e., infants <12 months of age with growth failure/ faltering), but where data for the population of interest is not reported separately.
Intervention	For wasting or growth failure/faltering: initiation of treatment in a community setting. initiation of treatment in outpatient settings. referral to treatment in an inpatient setting. transfer from inpatient to outpatient/community treatment discharge from outpatient/community treatment .	Other interventions that are not those listed in the inclusion criteria.
Comparators	Not restricted (with or without a comparator)	N/A
Outcomes	Resource use Costs Cost-effectiveness estimates based on a) cost outcome analysis (e.g., cost per child seen etc.), or b) full cost-effectiveness analysis (e.g., cost per life years saved etc.).	Only indirect costs reported, such as productivity loss. Only including costs of medicinal food with no setting-related costs
Study type	Any type of economic analysis (including cost and cost-effectiveness or cost-utility analyses) reporting cost estimates based on a) patient-level data, b) expenditure or c) ingredients, or a combination thereof, or calculating costs based on treatment pathways in clinical guidelines	Systematic reviews and other types of literature reviews to avoid double counting
Language	No restrictions	N/A
Other	Studies that are available as full text	Publications which do not report relevant outcomes (e.g., study protocols, commentaries and letters for the Editor)

### Search strategy

The following databases were searched on 10^th^ December 2021: Global Health Cost Effectiveness Analysis Registry and the Cost-Effectiveness Analysis Registry via the Center for the Evaluation of Value and Risk in Health; Cochrane Central Register of Controlled Trials (CENTRAL) and Cochrane Database of Systematic Reviews via Cochrane Library; CRD’s NHS Economic Evaluation Database and HTS Database (available only until 2015); EconLit via ProQuest Dialog; Embase via Ovid SP; Epistemonikos; Google Scholar (including Grey Literature); INAHTA HTA Database; and Ovid MEDLINE ALL. The search terms were selected from experts’ opinions, literature review, reviewing the results of scoping searches, and controlled vocabularies (Medical Subject Heading = MeSH and Excerpta Medica Tree = Emtree). The terms were arranged into three blocks: Block 1, terms for children/ infants; Block 2, terms for wasting or growth failure; and Block 3, terms for study design or outcomes (e.g., cost analysis, cost-effectiveness etc). No date, study design, publication type, geographic or language limits were imposed on the searches. All search strategies are reported in S3 File.

We also searched the websites of Action Against Hunger, MSF, Save the Children, UNICEF, WHO, and the World Bank. Citations and reference lists of included publications and previous systematic reviews were also manually reviewed to identify additional literature.

### Study selection

The Rayyan software was used to manage the articles retrieved from the searches [14]. Each article was independently screened for eligibility by two independent reviewers using a piloted study screening form based on the inclusion/exclusion criteria. Titles and abstracts were screened during the first stage of study selection. Studies judged to be potentially eligible in the first stage had their full texts screened in the second stage.

### Data extraction

For each of the included studies, two reviewers independently extracted the relevant data using a standardised Microsoft Excel data extraction table. The table was piloted on five studies before use and adjusted accordingly [11, 12]. The extracted data included general information such as author, publication year, country, WHO region; study methodology; population; details of intervention; and outcomes (for more details see S4 File).

### Quality assessment strategy

The included studies were published between 1972 and 2021. Methodological or reporting quality was assessed using the 2013 ISPOR Consolidated Health Economic Evaluation Reporting Standards (CHEERS), which is the guidance that was applicable during that period [15]. Each item on the checklist was graded for each study as follows: 0 (not considered), 1 (partially considered), 2 (fully considered) and N/A (if not relevant to the study). The item scores were subsequently summed up and a percentage calculated based on the maximum attainable score. Studies with a percentage score less than 50% were categorised as low, those with a score between 50% and 74% as moderate, and those with a score of 75% or higher as good quality studies. The Grading of Recommendations, Assessment, Development and Evaluations (GRADE) system and the UK National Institute for Health and Care Excellence’s economic profiles approach were used to classify the certainty in the evidence across all studies as very low, low, moderate, or high [16–18]. First, we built an economic profile for the available evidence for each topic using the following criteria: resource allocation, cost-effectiveness evidence, overall quality of evidence, applicability, certainty and any other limitations (Table 2).

**Table 2:** Criteria for economic profiles.

Criteria	Considerations	Rationale for judgement
Resource allocation	number of studies reporting the costs of an intervention how the costs compare with other models of care	the higher the costs of one model of care compared to the alternatives, the lower the likelihood that a strong recommendation was warranted [16]. The higher the number of studies reporting consistent results, the higher the likelihood of a strong recommendation.
Cost-effectiveness evidence	number of studies reporting the costs of an intervention and the incremental cost-effectiveness ratio when compared with other models of care against the appropriate threshold	if an intervention is cost-effective compared to the alternatives, a strong recommendation is warranted. The higher the number of studies reporting consistent results, the higher the likelihood of a strong recommendation.
Overall quality of evidence	Based on the CHEERS checklist	The higher the quality of the evidence, the higher the likelihood that a strong recommendation is warranted [16].
Applicability	How well does the included evidence answer the review question [17]? Are the study populations and the interventions being evaluated the same as those depicted in the review question? Are the comparisons being made between real-life/ viable alternatives [18]?	Directly applicable if the studies meet all applicability criteria or fail to meet one or more applicability criteria, but this is unlikely to change the conclusions about cost-effectiveness partially applicable if the studies fail to meet one or more of the applicability criteria, and this could change the conclusions about cost-effectiveness not applicable if the studies fail to meet one or more of the applicability criteria, and this is likely to change the conclusions about cost-effectiveness.
Certainty	The extent to which there was confidence that an estimate of an effect from the whole body of evidence was adequate to make a decision or a recommendation [18]?	The higher the confidence in the estimate, the higher the likelihood that a strong recommendation is warranted
Other limitations	Other limitations either identified in the study report itself, or by the reviewers.	What are the implications on the confidence in the estimates?

Evidence based on cost-effectiveness analysis was considered as high quality. For each model of care, each of the criteria above was given a rating [16]. If no serious concern existed for any of these criteria, the recommendation was not downgraded; if serious concern existed for at least one of the criteria, the evidence was downgraded one level (-1), e.g., from high to moderate. In the case of very serious concern for at least one of the criteria, the downgrade was two levels (-2), e.g., from high to low. Evidence that was only based on cost analysis was considered as low quality, with upgrades (i.e., +1 or +2) for large effect, dose-response, or no confounding.

We used the GRADE definitions where the quality of the evidence was considered as [16].

- high when there was strong confidence that the true value lies close to the estimated value,
- moderate when the true value was likely to be close to the estimated value, but there was a possibility that it was substantially different,
- low when the true value could be substantially different from the estimated value,
- very low when the true value was likely to be substantially different from the estimated value.

One reviewer independently made these judgements, with another reviewer checking them.

Disagreements between reviewers on study selection, data extraction or quality assessments were resolved by discussion; and where consensus could not be reached, they were resolved through referral to a third reviewer.

### Data synthesis

The studies were grouped as follows:

- Management of growth failure/faltering in infants
- Management of moderate wasting
- Management of severe wasting and/or bilateral pitting oedema
- Management of moderate wasting and severe wasting and/or bilateral pitting oedema together

Within these main groups, studies were sub-divided into sub-groups according to the type of management and setting (e.g., initiation of treatment in a community setting, initiation of treatment in outpatient settings, etc.). When reporting our results within these subgroups, we distinguished between evidence from studies of children 6 to 59 months versus 0 to 59 months; or studies of infants <6 months versus <12 months. This was in order to align our work with the evidence requirements of the WHO guidelines for the prevention and management of acute malnutrition in infants and children under 5 years.

Where appropriate, we summarized quantitative outcomes descriptively using means, medians and ranges according to the perspectives adopted by the included studies. This descriptive analysis was conducted using Microsoft Excel. We also used tables, graphs and figures to present and visualize the data. There was significant heterogeneity with regards to interventions, settings, resource use, costs and costing methods such that pooled estimates would not generate robust or meaningful results [19, 20]. We, therefore, performed narrative syntheses to summarize the study results within the sub-groups.

To allow for comparability at an international level, all costs were converted to 2020 US dollars using purchasing power parity (PPP) exchange rates which account for variations between countries in the costs of goods and services [21, 22]. 2021 PPP exchange rates were not available at the time of data extraction. Conversion to 2020 USD was done after allowing for inflation using country-specific consumer price indexes [23].

### Presentation to the WHO Guideline Development Group (GDG)

The methods and results of the systematic review were shared with the WHO Prevention and Management of Wasting and Nutritional Oedema GDG for validation. The group comprises interdisciplinary international experts in child nutrition. We also presented our findings to this group during their GDG meeting on 21 to 24 March 2023. During the meeting, the GDG members discussed the review findings and asked any clarification questions. We revised our review according to the feedback provided where necessary.

## Results

### Search results

**Fig 1:**
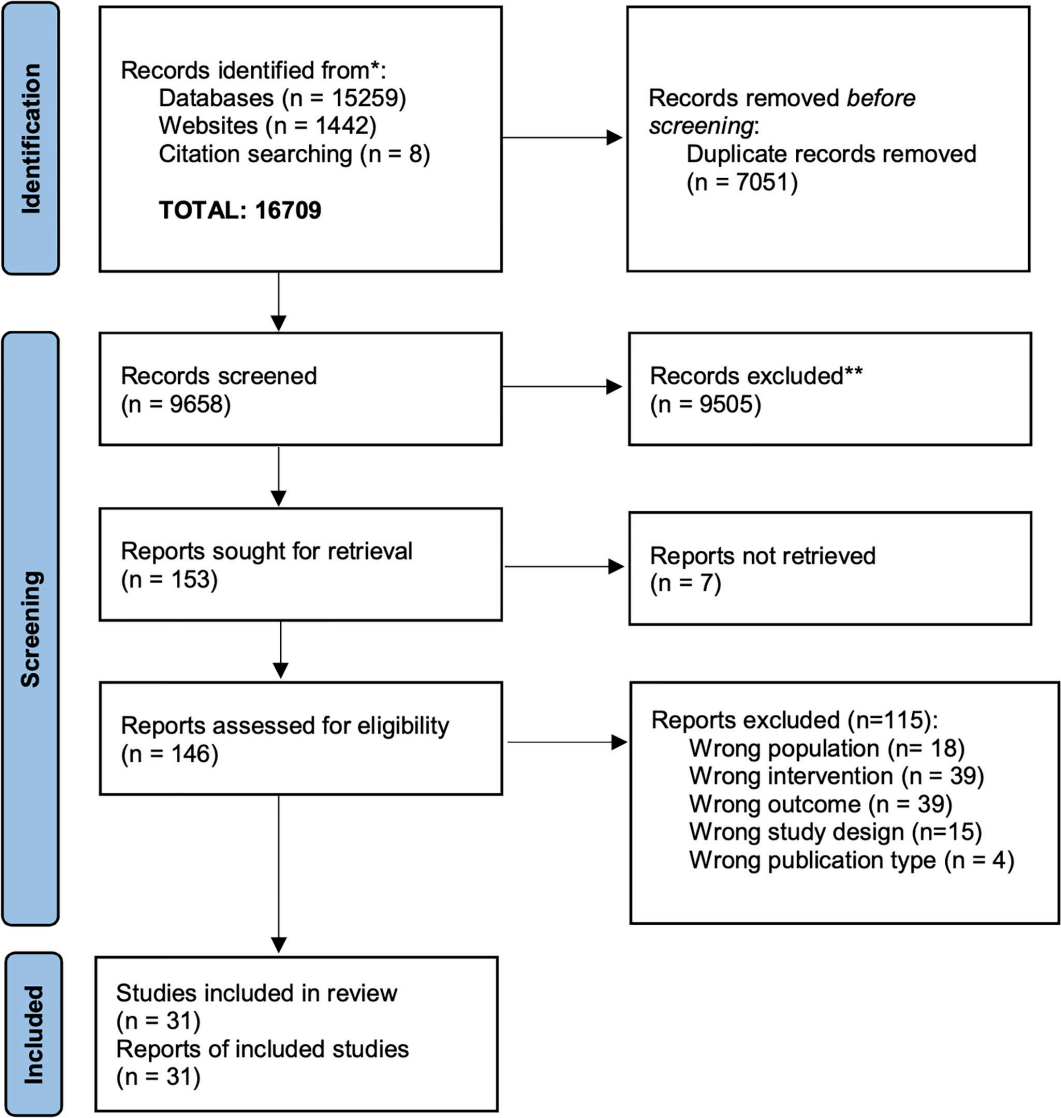
Flow of studies through the review process.

A total of 16,709 records were identified (Fig 1). The titles and abstracts of 9663 records were screened after removing duplicates. Of the 153 full texts sought for retrieval, seven either did not have a full text [24] or could not be retrieved even after contacting the authors [25–30]. 146 full texts were retrieved and screened, and 115 records were excluded [31–145]. Thus, 31 reports [146–176] representing 31 unique studies were included in the review. Details of excluded studies are provided in S5 File.

### General study characteristics

General study characteristics are shown in Table 3. Below is a narrative summary of the following characteristics: WHO region in which each study was conducted, study population, intervention, setting, outcomes explored, type of economic evaluation and cost items and costing methods. The study-level definitions for moderate wasting, severe wasting and/or bilateral pitting oedema and growth failure/faltering are provided as S6 File.

**Table 3:** General characteristics of included studies.

Author, year	Country, WHO region	Population	Intervention	Setting (rural/urban), level of care/treatment setting	Outcomes	Study design	Type of economic evaluation	Cost data collection method	Cost perspective	Sample size	Reporting quality based on CHEERS checklist and % score^+^
Akram (2016) [146]	Pakistan; Eastern Mediterranean	Severe wasting and/or bilateral pitting oedema; 6–23 months	Initiation of treatment in a community setting	Rural; home	Cost per child rehabilitated	Retrospective analysis	Cost analysis	NR	Provider	123	Low; 39%
Ali (2017) [147]	Nigeria; Africa	Severe wasting and/or bilateral pitting oedema; <60 months^#^^	Initiation of treatment in outpatient settings	Not reported (NR); Outpatient therapeutic centre	Cost per: child, DALY averted, & life saved	Decision analytic model	Cost-effectiveness analysis (CEA)	Bottom-up	Societal	NR	Good; 79%
Ashraf (2019) [148]	Bangladesh; South-East Asia	Severe wasting and/or bilateral pitting oedema; 2–59 months^#^^	Initiation of treatment in outpatient settings	Urban; day clinic	Cost per child treated	RCT	Cost analysis	Ingredients	Societal	235	Moderate; 55%
			Initiation of treatment in an inpatient setting	Urban; Inpatient hospital care						235	
Ashworth (1997) [149]	Bangladesh; South-East Asia	Severe wasting and/or bilateral pitting oedema; 12–60 months	Initiation of treatment in an inpatient setting	Urban; children’s nutrition unit	Cost per child rehabilitated	RCT	Cost analysis	Bottom-up & ingredients	Societal	173	Moderate; 66%
			Transfer from outpatient treatment at a health facility day care centre to domiciliary care	Urban; household care						130	
			Initiation of treatment in outpatient settings	Urban; health facility day care centre						134	
Bachmann (2009) [150]	Zambia; Africa	Severe wasting and/or bilateral pitting oedema; <60 months^#^^	Initiation of treatment in outpatient settings	Urban; PHC	Cost per: child, death averted & DALY averted	Decision analytic modelling	CEA	Ingredients	Provider	2523	Good; 95%
Bai (1972) [151]	India; South-East Asia	Severe wasting and/or bilateral pitting oedema; <60 months^#^^	Initiation of treatment in outpatient settings	Rural; health centre	Cost per child	Prospective cohort	Cost analysis	Bottom-up	NR	25	Low; 44%
Bailey (2020) [152]	Kenya, South Sudan; Africa	Moderate wasting and Severe wasting and/or bilateral pitting oedema; 6–59 months	Initiation of treatment in outpatient settings	Rural & urban; PHC	Cost per child recovered & incremental cost per child recovered	Cluster-randomised controlled non-inferiority trial	CEA	Ingredients & bottom-up	Societal	2071; 2039	Good; 84%
Chapko (1994) [153]	Niger; Africa	Moderate wasting and Severe wasting and/or bilateral pitting oedema; 5–28 months	Transfer from inpatient to outpatient	Urban; ambulatory rehabilitation centre	Cost per child treated	RCT	Cost analysis	Bottom-up	Provider	47	Moderate; 58%
			Transfer from an inpatient to another inpatient facility	Urban; hospital						53	
Fotso (2019) [154]	Ethiopia; Africa	Severe wasting and/or bilateral pitting oedema; <60 months^#^^	Initiation of treatment in outpatient settings	NR; Health centres and health posts	Cost per: child cured, death averted & DALY averted	Prospective cohort	CEA	Bottom-up	Societal	891; 1,286	Good; 78%
Frankel (2015) [155]	Nigeria; Africa	Severe wasting and/or bilateral pitting oedema; NR months	Initiation of treatment in outpatient settings	NR; PHC	Cost per: child cured, death averted, & DALY averted	Prospective cohort and CEA model	CEA	Bottom up	Societal	NR	Good; 91%
Garg (2018) [156]	India; South-East Asia	Severe wasting and/or bilateral pitting oedema; 6–59 months	Initiation of treatment in a community setting	Urban; home	Cost per: child treated & child recovered	RCT	Cost analysis	Bottom-up	Provider	124; 123	Moderate; 68%
Gomez (1983) [175]	Chile; Americas	Moderate wasting and Severe wasting and/or bilateral pitting oedema; 0–23, 24–71 & 0–71 months^#^^	Treatment initiation in the outpatient	Urban; Outpatient	Cost per: child recovered/day & child/day	Retrospective Cohort	Cost analysis	Top down	Societal	745	Moderate; 62%
			Treatment initiation in the community	Urban; Kindergarten						420	
International Rescue Committee (IRC) (2016) [157]	Mali; Africa	Severe wasting and/or bilateral pitting oedema; <60 months^#^^	Initiation of treatment in outpatient settings	NR; PHC	Cost per child treated	Retrospective cohort	Cost analysis	NS	Provider	2838; 2874; 6324	Moderate; 50%
	Niger; Africa									4976	
	Kenya; Africa									4,000*; 4,250*	
	Yemen; Eastern Mediterranean									400*; 250*	
Isanaka (2017) [160]	Niger; Africa	Severe wasting and/or bilateral pitting oedema; 6–59 months	Initiation of treatment in a community/inpatient/outpatient settings	Rural; hospital	Cost per child treated	Cross-sectional	Cost analysis	Top-down	Provider	6,903	Moderate; 74%
				Rural; community						13,395	
				Rural; hospital & community						20,298	
Isanaka (2019) [158]	Mali; Africa	Moderate wasting and Severe wasting and/or bilateral pitting oedema; 6–35 months	Initiation of treatment in outpatient settings	Rural; community health centre	Cost per child identified, incremental cost per: death & DALY averted	Decision tree: Cluster-randomised trial	CEA	Ingredients & bottom-up	Provider	1,766	Good; 87%
Karniski (1986) [159]	USA; Americas	Growth faltering; <12 months^	Transfer from inpatient to community settings	Urban; medical placement home	Cost per child treated	Retrospective cohort	Cost analysis	Ingredients and top-down	Provider	17	Moderate; 55%
			Initiation of treatment in an inpatient setting	Urban; hospital						18	
Masiiwa (2013) [161]	Zimbabwe; Africa	Severe wasting and/or bilateral pitting oedema; 0–59 months^#^^	Referral to treatment in an inpatient setting	Urban; hospital	Cost per household	Cross sectional study	Cost analysis	Bottom-up	Household	142	Good; 84%
N’Diaye (2020) [162]	Burkina Faso; Africa	Severe wasting and/or bilateral pitting oedema; 6–59 months	Initiation of treatment in outpatient settings	Urban & non-urban; PHC	Cost per child treated	Decision analytic model (Part of a clinical trial)	Cost minimisation analysis	Top-down & bottom-up	Societal	399; 399	Good; 92%
Nkonki (2017) [163]	South Africa; Africa	Moderate wasting; 0–59 months^#^^	Initiation of treatment in a community setting	NR; community	Total cost	Deterministic mathematical model	Cost analysis	Ingredients	Provider	NR	Moderate; 63%
		Severe wasting and/or bilateral pitting oedema; 0–59 months^#^^									
Puette (2013) [164]	Bangladesh; South-East Asia	Severe wasting and/or bilateral pitting oedema; 6–36 months	Referral to treatment in an inpatient setting	Rural; Upazila health complex	Cost per: child treated, recovered, death averted, & DALY averted	Cost model	Cost analysis	Top-down & bottom-up	Societal	633	Good; 80%
			Initiation of treatment in outpatient settings	Rural; community						724	
Purwestri (2012) [165]	Indonesia; South-East Asia	Moderate & mild wasting; 6–59 months	Initiation of treatment in a community setting	Semi-urban & rural; community	Cost per: child & child reaching discharge criterion	Cohort study	Cost analysis	Top-down & bottom-up	Societal	103;101	Moderate; 72%
Reed (2012a) [168]	Pakistan; Eastern Mediterranean	Moderate wasting; 6–59 months	Initiation of treatment in outpatient settings	Rural & urban; NS	Cost per beneficiary	Retrospective study	Cost analysis	Top-down	Provider	57,946	Moderate; 56%
				Rural & urban; hospital						NS	
				Rural & urban; outpatient centres						12,701	
Reed (2012b) [167]	Kenya; Africa	Severe wasting and/or bilateral pitting oedema; 6–59 months	Initiation of treatment in outpatient settings	Arid, semi-arid lands and urban; PHC	Cost per child treated	Cross-sectional study	Cost analysis	Top-down	Provider	13,501	Moderate; 56%
			Referral to treatment in an inpatient setting	Arid, semi-arid lands and urban; Hospital						990	
		Moderate wasting; 6–59 months	Initiation of treatment in a community setting	Arid, semi-arid lands and urban; Community based care						44,148	
Reed (2012c) [166]	Nepal; South-East Asia	Severe wasting and/or bilateral pitting oedema; 6–59 months	Initiation of treatment in outpatient settings	NR; PHC	Cost per child treated	Cross-sectional study	Cost analysis	Top-down	Provider	7,548	Moderate; 56%
		Moderate wasting; 6–59 months	Initiation of treatment in a community setting	NR; Community based care						40,769	
Rogers (2017) [169]	Malawi; Africa	Moderate wasting; 6–59 months	Initiation of treatment in a community setting	Rural; home	Cost per: treated beneficiary & additional caregiver meeting or exceeding target	Cross-sectional study	CEA	Top-down & bottom-up	Societal	196; 192; 196	Moderate; 63%
Rogers (2018) [171]	Mali; Africa	Severe wasting and/or bilateral pitting oedema; 6–59 months	Initiation of treatment in a community setting	Rural; community	Cost per: child treated & child recovered	Clinical cohort trial	CEA	Top-down & bottom-up	Societal	617	Good; 76%
Rogers (2019) [170]	Pakistan; Eastern Mediterranean	Severe wasting and/or bilateral pitting oedema; 6–59 months	Initiation of treatment in outpatient settings	NR; PHC	Cost per child recovered & incremental cost per additional child recovered	Cost model (Part of a clinical trial)	CEA	Top-down & bottom-up	Provider (institutions); Provider (government); Community	393	Good; 82%
			Initiation of treatment in a community setting	NR; community						425	
Tekeste (2012) [172]	Ethiopia; Africa	Severe wasting and/or bilateral pitting oedema; 6–59 months	Initiation of treatment in a community setting	Rural; community	Cost per: child cured & child treated	Retrospective comparative study	CEA	Top-down	Societal	157	Good; 82%
			Initiation of treatment in outpatient settings	Rural; therapeutic feeding centre						149	
UNICEF (2012) [176]	Chad; Africa	Severe wasting and/or bilateral pitting oedema; 0–59 months^#^^	Treatment initiation in the outpatient/inpatient	Rural; Outpatient	Cost per child	Descriptive analysis	Cost analysis	Top down and ingredients-based	Provider	NR	Low; 47%
Wilford (2011) [173]	Malawi; Africa	Severe wasting and/or bilateral pitting oedema; <60 months^#^^	Initiation of treatment in a community/inpatient/outpatient settings	NR; hospital/PHC/community	Cost per child treated & per DALY averted	Cross-sectional study	CEA	Top-down	Provider	3,577	Good; 84%
Wilunda (2021) [174]	Tanzania; Africa	Severe wasting and/or bilateral pitting oedema; 6–59 months	Initiation of treatment in outpatient settings	Rural; PHC	Cost per: child treated & cured & incremental cost per additional child cured	Non-inferiority quasi-experimental study (Part of a clinical trial)	CEA	Bottom-up	Provider	154	Good; 82%
			Initiation of treatment in a community setting	Rural; home						210	

^No subgroup analysis for those aged <6 months

^#^No subgroup analysis for those aged 6–59 months

^+^Excludes items 23 & 24 of the CHEERS checklist

#### WHO region

Sixty-one percent (19/31) of the studies included at least one country in the African Region (Table 3) [147, 150, 152–155, 157, 158, 160–163, 167, 169, 171–174, 176]. Twenty-three percent (7/31) of studies included countries in the South-East Asian region [148, 149, 151, 156, 164–166]. Thirteen percent (4/31) of studies included countries in the East Mediterranean [146, 157, 168, 170], and 6% (2/31) of studies included countries in the American region [159, 175]. Over 90% of the countries were LMICs. South Africa was the only upper-middle income country, while Chile and USA were the only high-income countries.

#### Study population

Twenty studies (65%) were on infants and children with severe wasting and/or bilateral pitting oedema [146–151, 154–157, 160–162, 164, 170–174, 176]. Three studies (10%) were among infants and children with moderate wasting [165, 168, 169]. There was only one study on growth faltering or failure (3%) [159]. The remaining 7 studies (23%) focused on both moderate and severe wasting and/or bilateral pitting oedema [152, 153, 158, 163, 166, 167, 175]. The age group covered by each study is shown in Table 3.

#### Interventions and settings

Nineteen studies (61%) evaluated the initiation of treatment in outpatient settings [147, 149–152, 154, 155, 157, 158, 162, 164, 166–168, 170, 172, 174–176] and 13 (42%) in community settings [146, 148, 156, 163, 165–167, 169–172, 174, 175]. Three studies (10%) evaluated referral from outpatients or community settings to inpatient settings [161, 164, 167], and another 3 (10%) were on the initiation of treatment in a mixture of settings [160, 173, 176]. There was one study (3%) each for transfer from outpatients to community settings [149], inpatients to another inpatient setting [153], inpatient to outpatient settings [153], and inpatient to community settings [159].

#### Outcomes

As shown in Table 3 the outcomes that were reported varied widely. These outcomes were often not clearly defined. When provided, definitions varied widely between studies for the same outcome. For example, the definition for cost per child treated varied from the cost per child admitted for treatment, regardless of the outcome [167], cost per child discharged regardless of the outcome [170, 173], through to cost per child admitted for treatment and successfully recovered [172]. For the purposes of this review, we assumed that "child recovered", "child cured" or “child rehabilitated” referred to the cost per child admitted for treatment and successfully recovered; whilst "child treated", "child covered", “child identified", "treated beneficiary", "child", and "beneficiary" referred to the cost per child admitted for treatment, regardless of the outcome.

#### Type of economic evaluation

Eighteen (58%) studies were cost or cost-efficiency analyses (Table 3; see S2 File for definitions) [146, 148, 149, 151, 153, 156, 157, 159–161, 163, 164, 166–168, 175, 176], and 12 studies (39%) were cost-effectiveness analyses [147, 150, 152, 154, 155, 158, 169–174]. N’Diaye and colleagues carried out a cost-minimisation analysis to identify the option that results in the lowest costs [162].

#### Cost items and costing methods

The items that were costed and the costing method used by each study are shown in Table 4. The type of costs and costing methods are also defined in S2 File.

**Table 4:** Costs and data collection methods.

Author, Year	Reference year of costs^+^	Costing method	Cost categories												
			Personnel	Training	Administrative	Capital	Diagnostics	Medication	Transport (provider)	Transport (household)	Home food	Specially formulated foods	Productivity loss	Work for care giver	Other
Akram (2016) [146]	2012	Incremental financial cost, NR approach	x						x			x			Parental counselling
Ali (2017) [147]	(2016)	Incremental economic cost, bottom-up approach	x		x			x				x			Community volunteer
Ashraf (2019) [148]	(2018)	Full economic cost, ingredients approach	x			x	x	x	x	x	x	x	x		Supportive care & hotel
Ashworth (1997) [149]	(1996)	Full financial cost, bottom-up & ingredients approach	x			x	x	x		x	x	x	x		Other parental costs
Bachmann (2009) [150]	2008	Incremental financial cost, ingredients approach		x								x			Hospitalisation, community mobilisation & health centre visits
Bai (1972) [151]	(1971)	Incremental economic cost, bottom-up approach										x	x		
Bailey (2020) [152]	2017	Incremental economic cost, ingredients & bottom-up approach	x			x			x			x			Outreach
Chapko (1994) [153]	(1993)	Full financial cost, bottom-up approach	x			x	x	x			x	x			
Fotso (2019) [154]	(2018)	Incremental economic cost, bottom-up approach	x	x	x			x	x	x		x	x		
Frankel (2015) [155]	(2014)	Full economic cost, bottom-up approach	x	x	x		x	x	x			x	x		
Garg (2018) [156]	2014	Incremental financial cost, bottom-up approach	x	x	x	x	x	x	x			x			Peer support
Gomez (1983) [175]	1979	Incremental Economic cost, top-down approach	x			x						x		x	Distribution and other
IRC (2016) [157]	(2015)	Full financial cost, NS approach; Incremental financial cost, NS approach	x		x	x		x				x			
Isanaka (2017) [160]	2015	Full financial cost, top-down approach	x		x	x	x	x	x			x			Non-medical equipment &supplies
Isanaka (2019) [158]	2015	Full financial cost, ingredients & bottom-up approach	x		x	x			x			x			Medical supplies & materials
Karniski (1986) [159]	(1985)	Incremental financial cost, ingredients and top-down approach	x		x				x			x			Hospital charges & overheads
Masiiwa (2013) [161]	(2012)	Incremental economic cost, bottom-up approach						x		x	x		x		
N’Diaye (2020) [162]	2017	Incremental economic cost, top-down & bottom-up approach	x					x				x	x		Material i.e., thermometers, height boards, weight scales, & transportation boxes
Nkonki (2017) [163]	2015	Incremental financial cost, ingredients approach	x			x		x							Other recurrent costs
Puette (2013) [164]	2010	Incremental economic cost, top-down & bottom-up approach	x	x		x		x		x	x	x			Other household costs
Purwestri (2012) [165]	2007	Incremental economic cost, top-down & bottom-up approach	x			x	x		x			x	x		Volunteer incentives
Reed (2012a) [168]	(2011)	Incremental financial cost, top-down approach	x			x									Utilities, medical equipment& service charges
Reed (2012b) [167]	(2011)	Incremental financial cost, top-down approach	x					x				x			
Reed (2012c) [166]	(2011)	Incremental financial cost, top-down approach										x			
Rogers (2017) [169]	2014	Incremental economic cost, top-down & bottom-up approach	x	x	x				x			x	x		Warehousing
Rogers (2018) [171]	2016	Incremental financial cost, top-down & bottom-up approach	x	x	x				x	x		x	x		Community level rent, utilities & rent
Rogers (2019) [170]	(2018)	Incremental economic cost, top-down & bottom-up approach	x	x	x				x			x	x		Community level rent, utilities & rent
Tekeste (2012) [172]	2006	Full economic cost, top-down approach	x			x		x		x	x	x	x	x	Caregiver’s food
UNICEF (2012) [176]	2010	Incremental Financial cost, top down and ingredients-based approach	x		x	x		x	x			x			Other
Wilford (2011) [173]	2007	Incremental costs financial cost, top-down approach	x	x	x	x		x	x			x			Inpatient costs
Wilunda (2021) [174]	2019	Incremental financial cost, bottom-up approach	x	x	x	x					x				Sensitization & mobilisation

### Management of growth failure/faltering in infants <12 months of age

There was one study on infants aged <12 months conducted in the USA which found that transfer from inpatient to community treatment in a medical placement home was $1003 more expensive per child treated than treatment in an inpatient setting from a provider perspective ($6,776 versus $5,773; Table 5, S1 Table) [159]. The study included those diagnosed with non-organic failure to thrive and excluded those with any indication of organic aetiology for the failure to thrive. The specific treatments provided for growth failure/faltering were not clearly specified, but the analysed costs included the costs of physicians, laboratory tests, radiology, medication and room charges. We did not find any study reporting on infants <6 months of age. No studies were found for the other treatment setting decisions and outcomes of interest in this group.

**Table 5:** Costs and cost-effectiveness of transfer and referral models of care.

	Community to outpatient	Community/outpatient to inpatient	Inpatient to outpatient/ community	Outpatient to community
***What are the associated costs***				
Growth failure/faltering in infants <12 months of age	No studies identified	No studies identified	*Age <12 months (to community)* **Provider costs** $6,776 per child treated	No studies identified
Moderate wasting among infant and children <60 months of age	No studies identified	No studies identified	No studies identified	No studies identified
Severe wasting and/or pitting oedema among infant and children <60 months of age	No studies identified	*Age 6 to 59 months* **Provider costs**: $298–$714 per child treated **Societal costs**: $5,465 per child treated $37,204 per child recovered *Age < 60 months* **Household costs**: $84 per household	No studies identified	*Age 12 to 60 months* **Provider costs**: $163 per child recovered **Parentals costs**: $52 per child recovered
Moderate wasting and severe wasting and/or bilateral pitting oedema together among infants and children <60 months of age	No studies identified	No studies identified	*Age 5 to 28 months (to outpatient)* **Provider costs**: $98 per child treated	No studies identified
***What is the cost-effectiveness***				
Growth failure/faltering in infants <12 months of age	No studies identified	No studies identified	No studies identified	No studies identified
Moderate wasting among infant and children <60 months of age	No studies identified	No studies identified	No studies identified	No studies identified
Severe wasting and/or pitting oedema among infant and children <60 months of age	No studies identified	*Age 6 to 59 months* **Societal perspective**: $5465 per DALY averted (compared to no treatment)	No studies identified	No studies identified
Moderate wasting and severe wasting and/or bilateral pitting oedema together among infants and children <60 months of age	No studies identified	No studies identified	No studies identified	No studies identified

### Management of moderate wasting in infants and children <60 months of age

Studies identified in this group reported on costs, cost-efficiency and/or cost-effectiveness of initiating treatment in community or outpatient settings as summarised below.

#### Initiation of treatment in community settings

The costs or cost-efficiency of initiating treatment for moderate wasting in community settings were reported by five studies: four in those aged 6 to 59 months [165–167, 169] and one in those aged 0 to 59 months [163]. The cost per child treated varied widely: from the provider perspective this was from $18 to $934 [165–167], and from a societal perspective it was from $237 to $1,380 (Table 6; S2 Table) [165, 169]. The treatments provided varied from the provision of counselling, which seemed to incur the lowest cost, to the provision of supplementary food (e.g., $18 for counselling [166] versus $943 for weekly supplementary food distribution from the provider perspective [165]).

**Table 6:** Costs and cost-effectiveness of initiating treatment in community and outpatient settings.

	Community setting	Outpatient setting
***What are the associated costs***		
Growth failure/faltering in infants <12 months of age	No studies identified	No studies identified
Moderate wasting among infant and children <60 months of age	*Age 6 to 59 months* **Provider costs**: $18 -$943 per child treated $467 & $502 per child recovered **Societal costs**: $237 -$1,380 per child treated $604 & $822 per child recovered *Age <60 months* **Total costs**: Total provider costs of nearly $6 million	*Age 6 to 59 months* **Provider costs**: $118 -$277 per child treated
Severe wasting and/or pitting oedema among infant and children <60 months of age	*Age 6 to 59 months* *Initiation at home* **Provider costs**: $1,779 -$2,047 per child treated $13 per child surveyed $126 per child recovered *Initiation through health workers working in proximity to health facilities or through community health workers* **Provider costs**: $202 to $799 per child treated $270 to $1051 per child recovered **Societal costs**: $671–$757 per child treated $732–$817 per child recovered **Community costs**: $64 per child treated *Age <60 months* **Total costs**: Total provider costs of nearly $6 million	*Age 6 to 59 months* **Provider costs**: $103–$1267 per child treated $239 to $1369 per child recovered **Societal costs**: $295 to $1597 per child treated $1419 to $1796 per child recovered **Community costs**: $117 per child treated $141 per child recovered **Parental costs**: $25 per child recovered *Age < 60 months* **Provider costs**: $67 to $3374 per child treated **Societal costs**: $76 to $529 per child treated $438 to $1129 per child recovered **Household costs**: $55 per child treated
Moderate wasting and severe wasting and/or bilateral pitting oedema together among infants and children <60 months of age	**Societal costs**: Cost per child recovered per day of $15 for children and infants aged 0 to 23 months, and $ 21 for those aged 24 to 71 months	**Societal costs**: Cost per child recovered per day of $6 for children and infants aged 0 to 23 months, and $11 for those aged 24 to 71 months
***What is the cost-effectiveness***		
Growth failure/faltering in infants <12 months of age	No studies identified	No studies identified
Moderate wasting among infant and children <60 months of age	No studies identified	No studies identified
Severe wasting and/or pitting oedema among infant and children <60 months of age	*Age 6 to 59 months* **Provider perspective**: $391 per additional child treated, $381 per additional child cured; Outpatient care as reference	*Age 6 to 59 months* **Provider perspective**: $1483 per child recovered; compared to community treatment by lady health workers **Societal perspective**: $106 per DALY averted compared to no treatment $3534 per death averted compared to no treatment *Age < 60 months* **Provider perspective**: $20 to $145 per DALY averted; compared to doing nothing or no programme implementation scenarios **Societal perspective**: $68 to $161 per DALY averted; compared to doing nothing or no programme implementation scenarios
Moderate wasting and severe wasting and/or bilateral pitting oedema together among infants and children <60 months of age	No studies identified	No studies identified

In the counselling support programme in Nepal the cost per child treated was $18 from a provider’s perspective [166]. Supplementary foods were only provided in food security emergencies [166]. In Kenya where the cost per child treated from a provider’s perspective was $201, ready-to-use therapeutic food (RUTF) was provided [167]. In Indonesia, Purwestri et al reported costs per child recovered of $502 and $822 for daily distribution of supplementation food in a semi-urban community from the provider and societal perspectives, respectively [165]. In a rural community, the costs per child recovered were $467 and $604 for weekly distribution of supplementation food from the provider and societal perspectives, respectively [165].

In South Africa, Nkonki et al reported total provider costs of nearly $6 million for a multicomponent intervention package that included the provision of supplementation food, maternal education and provision of oral rehydration solution for diarrhoea and oral antibiotics for suspected respiratory infections [163]. This study did not report the sample size.

In Malawi, Rogers et al [169] found that when compared to a control of monthly rations of 1 litre oil and 8kg corn-soy blend (CSB) and social and behaviour change communication (SBCC), an increased oil allocation (to 2.6 litres of oil for the 8 kg of CSB), either in bulk or four packages, plus enhanced SBCC were cost-effective at $249 and $396 per additional caregiver meeting or exceeding the recommended target of 30g oil:100g CSB respectively (S3 Table).

#### Initiation of treatment in outpatient settings

From the provider’s perspective, the costs per child treated for the initiation of treatment in outpatient settings for infants and children within the age group 6 to 59 months ranged between $118 and $277 from two studies [158, 168] (Table 6; S2 Table). The programme in Pakistan resulting in a cost per child treated of $118 used ready-to-use supplementary food (RUSF), RUTF and fortified blended foods [168]. From the study by Isanaka et al in Mali, the cost per child treated ranged from $247 to $277 and did not seem to vary much with the different types of foods used, i.e., RUSF; a specially formulated corn–soy blend (CSB); Misola, a locally produced, micronutrient-fortified, cereal–legume blend (MI); or locally milled flour (LMF) [158]. Nevertheless, out of the four dietary supplements, moderate wasting treatment with RUSF was the most cost-effective across a wide range of cost-effectiveness thresholds, including WHO’s gross domestic product (GDP) per capita threshold, with incremental cost-effectiveness ratios of $963 per DALY averted and $27,246 per death averted compared to no treatment of moderate wasting (S3 Table) [158]. Treatment using the other three dietary supplements were dominated (S3 Table) [158].

Unfortunately, there were no head-to-head comparisons between settings to enable judgements on whether one setting resulted in less costs or was more cost-efficient or cost-effective than the other.

### Management of severe wasting and/or bilateral pitting oedema in infants and children <60 months of age

Studies in this group covered initiation of treatment in community settings; initiation of treatment in outpatient settings; referral to treatment in an inpatient setting; and transfer from outpatient treatment to treatment in a community setting. Three studies also reported on initiating treatment in a mixture of settings.

#### Initiation of treatment in community settings

There were nine studies in total identified in this group. Three of them reported on the initiation of treatment within the home for infants and children within the age group of 6 to 59 months [146, 156, 174] (S4 Table). One of these three studies was conducted in India, and the costs to the government per child treated ranged from $1,779 to $2,047, depending on the type of RUTF used [156] (Table 6; S4 Table). Centrally produced RUTF (RUTF-C) incurred the lowest costs, followed by locally prepared RUTF (RUTF-L), with micronutrient-enriched (augmented), energy-dense, home-prepared food (A-HPF) incurring the highest costs. They also reported a cost of $13 per child surveyed within the community from a provider (government) perspective. Treatment was initiated by Accredited Social Health Activist (ASHA)-like workers (ALW) in a research setting. The second study, conducted in Pakistan, involved a team comprising a doctor, a lay health supervisor, a lady health worker and a project supervisor visiting malnourished children in their homes to initiate treatment [146]. The study reported a cost per child recovered of $126 from a provider perspective using a locally produced indigenous high-density diet (HDD) plus ’Baby Active’, a micronutrient powder sprinkled on any food the child consumed [146]. In Tanzania, Wilunda et al evaluated an intervention where treatment was at home using RUTF (type not specified), with the dosage based on a child’s body weight [174]. Children were followed up through community health worker weekly home visits [174]. The reports costs were $426 per child treated and $470 per child recovered from a provider perspective.

The remaining six studies involved initiation of treatment within community settings either by health workers working in proximity to health facilities (Niger) [160], or by community health workers (covering Bangladesh, Pakistan, Mali, South Africa and Ethiopia) [163, 164, 170–172]. In certain cases, non-governmental organisations also provided outpatient care to complement these initiatives [170]. From the five that were among infants and children within the age group of 6 to 59 months, the cost per child treated ranged from $202 to $799 from the provider perspective, and from $671 to $757 from a societal perspective (Table 6; S4 Table). The cost per child recovered ranged from $270 to $1,051 from the provider perspective, and from $732 to $817 from a societal perspective. Rodgers et al also reported a cost per child treated of $64 and a cost per child recovered of $84 from the community perspective [170]. In South Africa, Nkoki et al’s total programme costs for the treatment of children aged 0 to 59 months were about $13 million from the provider perspective but the population size was undocumented [163].

In the study by Isanaka et al in Niger where treatment initiation was by health workers, patients were provided with rations of RUTF, routine medical treatment and received weekly visits from health staff [160]. The remaining five studies involved community setting treatment initiation by community health workers. In Bangladesh, nutritional treatment included a weekly ration of RUTF, a single oral dose of folic acid (5 mg) and oral cotrimoxazole [164]. In Pakistan, there was provision of medical and nutritional treatment, and counselling on nutrition and Infant and Young Child Feeding practices in the patient’s home [170]. In Mali, Rodgers et al integrated the treatment of severe wasting into an existing Integrated Community Case Management programme [171]. In Tekeste et al’s study in Ethiopia, treatment was in community-based therapeutic centres, and included medicines to treat complications and therapeutic foods [172]. Patients also received care from community volunteers and daily visits from a nurse [172]. In South Africa, the costs were estimated from a multicomponent intervention package that included the provision of supplementation food, maternal education, oral rehydration solution for diarrhoea and oral antibiotics for respiratory infections [163].

In terms of cost-effectiveness, Wilunda et al reported that initiation of treatment in community settings was cost-effective, with an estimated incremental costs per additional child treated or cured of $391 and $381, respectively, when compared to initiation of treatment in outpatient settings in Tanzania using WHO’s GDP/capita threshold [174] (Table 6; S5 Table).

#### Initiation of treatment in outpatient settings

There were 17 studies included in this group (S4 Table). Initiation of treatment in outpatient settings was mostly in primary health clinics [150, 151, 154, 155, 157, 158, 162, 166–168, 170, 171, 174], with two at therapeutic feeding centres [147, 172] and two at day centres [148, 149]. The studies covered Bangladesh, Burkina Faso, Ethiopia, India, Kenya, Mali, Nepal, Niger, Nigeria, Pakistan, Tanzania, Yemen and Zambia.

Ten of the 17 studies were among children aged 6 to 59 months [149, 158, 162, 166–168, 170–172, 174]. For these 10 studies, the cost per child treated ranged from $103 to $1267 from a provider perspective, $205 to 1597 from a societal perspective, and $117 from a community perspective (Table 6; S4 Table). The cost per child recovered was between $239 and $1369 from a provider perspective; ranged from $1419 to $1796 from a societal perspective; approximately $141 from one study from a community perspective; and $25 for the parents.

The remaining seven studies were among children aged <60 months and did not include a subgroup analysis for those aged 6 to 59 months [147, 148, 150, 151, 154, 155, 157]. We were able to summarise costs for six of these. The cost per child treated ranged from $67 to $3374 from a provider perspective; $76 to 529 from a societal perspective, and approximately $55 from a household perspective (Table 6; S4 Table). The cost per child recovered ranged from $438 to $1129 from a societal perspective. The remaining study, Bai et al, reported a cost of $6,365 per child treated in India, but the perspective was not clear [151].

For most of the 17 studies under this group, care was provided by health workers, mostly nurses, with Lady Health Workers providing care in one study in Pakistan [168]. Most studies included the provision of RUTF, micronutrients such as folic acid or Vitamin A, broad-spectrum antibiotics and health education. Health education included encouragement to breastfeed, milk-based therapeutic diets or other modified diets (e.g., increasing the intake of pulses, nuts and eggs, rice-based meals etc) [148, 149, 151].

Six studies carried out cost-effectiveness analyses of initiation of treatment of severe wasting and/or bilateral pitting oedema in outpatient settings (S5 Table) [147, 150, 154, 155, 164, 170]. These studies reported cost per disability-adjusted life year (DALY) gained/averted [147, 150, 154, 155, 164], cost per death averted [150, 154, 155, 164], cost per life saved [147], and cost per additional child recovered [170] (Table 6; S5 Table). Only two of these studies were specific to the age group 6 to 59 months. One of these two, conducted in Bangladesh, reported that initiating treatment in outpatient settings was highly cost-effective when compared to no treatment, with a cost per DALY averted of $106 and a cost per death averted of $3534 from a societal perspective [164]. Inpatient treatment initiation costed much more by comparison, $5,465 per DALY averted and $185,787 per death averted when compared to no treatment from a societal perspective. The second study, conducted by Rogers et al in Pakistan, had a cost per additional child recovered of $1,483 for outpatient treatment when compared to community treatment by lady health workers from a provider perspective [170]. The differences in costs and recovery rates between the two strategies were small, which resulted in uncertainty in terms of which strategy was the most cost-effective.

The remaining four studies included infants and children <60 months in Nigeria [147, 155], Ethiopia [154] and Zambia [150]. When compared to doing nothing or no programme implementation, initiation of treatment in an outpatient setting was considered to be highly cost-effective using country-specific WHO GDP per capita thresholds. Cost per DALY gained/averted were $20 [150] and $145 [147] from the provider perspective, and $68 [155] and $161 [147] from a societal perspective (Table 6; S5 Table). Fotso et al reported that incorporating a surge approach in Ethiopia to strengthen the health system’s resilience against seasonal ‘surges’ in the demand for treatment of acute malnutrition, and a standard approach of delivering SAM treatment in outpatient were both highly cost-effective using the GDP per capita threshold [154]. However, the surge approach was reported as less cost effective with a cost per DALY averted of $70 (95%CI: $53–$91) when compared to the standard service which had a cost per DALY averted of $35 (95%CI: $27–$48). One study also estimated a cost per death averted from a provider perspective of $677 for initiating treatment in outpatient settings compared to doing nothing in Zambia [150], and this was $2,480 from a societal perspective from one study in Nigeria [155]. From the study by Fotso et al the cost per death averted were $4,973 (95%CI: $3,640–$7,089) with the surge approach, and $2,480 (95%CI: $1,891–$3,781) for the standard services. Ali et al also reported a cost per life saved of $5,376 from a provider perspective and $5,951 from a societal perspective for initiating treatment in outpatient settings when compared to no programme implementation (S5 Table) [147].

#### Referral to treatment in an inpatient setting

There were four studies identified for this group. Two studies included an investigation of the costs of a model of care where children aged 6 to 59 months with severe wasting and/or bilateral pitting oedema and medical complications started their treatment in community and outpatient settings and were then referred for stabilisation in inpatient settings [167, 168]. The costs per child treated were $298 in Kenya [167] and $714 in Pakistan [168] from the provider perspective (Table 5; S4 Table). In the study by Puette et al in Bangladesh, a care model involving referral from trained community health workers to inpatient hospital treatment among 6- to 36-month-olds with severe wasting and/or bilateral pitting oedema resulted in costs of $5,465 per child treated and $37,204 per child recovered from a societal perspective [164]. This was not cost-effective from a societal perspective, at $5,465 per DALY averted when compared to no treatment (Table 5; S5 Table) [164]. Masiiwa had a cost per household of $84 for a model of care where children aged less than 60 months with severe wasting and/or bilateral pitting oedema were referred from community and outpatient primary health care clinic treatment to hospital inpatient clinic in Zimbabwe (Table 5; S4 Table) [161].

#### Transfer from outpatient treatment to treatment in a community setting

In their study in Bangladesh, Ashworth and Khanum included a group of children, aged from 12 to 60 months, that were first treated for one week in the day care facility before sending them to be cared for at home [149]. During their care at home, they were visited by specially trained health care workers every week for one month, and then fortnightly, until reaching a weight-for-height that is 80% of the National Centre for Health Statistics median. The cost per child recovered was $163 from the provider perspective and $52 from the household perspective for this model of care (Table 5; S4 Table).

#### Initiation of treatment in multiple settings

Three studies were identified for this group. In Niger, Isanaka et al reported a cost per child treated of $399 for a cohort where some children, aged 6 to 59 months, initiated treatment in hospital and some in community settings [160]. In the Chad UNICEF study among children aged 0 to 59 months, initiation of treatment in outpatient or community settings resulted in a cost per child treated of $505 [176]. In the study by Wilford et al in Malawi among the 0 to 59 months age group, some initiated treatment in hospital, some in primary health care and some in community settings [173]. The cost per child treated was $445 when including standard health services, and $44 when the standard health service was excluded, from a provider perspective [173]. The cost-effectiveness analysis resulted in a cost per DALY averted of $110 when standard health services were included versus when they were not [173].

### Management of moderate wasting and severe wasting and/or bilateral pitting oedema together in infants and children <60 months of age

Studies identified for this group reported the cost and cost-effectiveness analysis for initiating treatment in outpatient settings and transfer from inpatient to outpatient/community settings.

#### Initiation of treatment in outpatient settings

There were two studies identified for this group. Bailey et al estimated the cost of initiation of treatment in outpatient settings for infants and children aged 6 to 59 months in Kenya [152]. They found that, from a societal perspective, using different standard protocols for severe wasting and/or bilateral pitting oedema and moderate wasting was $337 more expensive per child recovered compared to using the same combined protocol for both severe wasting and/or bilateral pitting oedema and moderate wasting (S6 Table) [152]. In a study by Gomez et al in Chile, the costs of outpatient treatment for those with severe wasting and/or bilateral pitting oedema or moderate wasting were $11 per child recovered per day for those 0 to 23 months old, and $5.89 per child recovered per day for those aged 24 to 71 months, from a societal perspective [175]. The household cost per child treated per day was $5.50 for children aged 0 to 71 months.

#### Initiation of treatment in community settings

In Chile, the costs of initiating treatment at a kindergarten for infants and children with severe wasting and/or bilateral pitting oedema or moderate wasting were $21 per child recovered per day for those 0 to 23 months old, and $15 per child recovered per day for those aged 24 to 71 months, from a societal perspective [175]. The household cost per child treated per day was $1.49 for children aged 0 to 71 months.

#### Transfer from inpatient to outpatient

In a study by Chapko et al in Niger, infants and children with severe wasting and/or bilateral pitting oedema or moderate wasting were first treated in the paediatric service of the national hospital and then randomised to nutritional rehabilitation in either a hospital inpatient setting or ambulatory setting [153]. Ambulatory treatment involved attendance at an ambulatory rehabilitation centre each day. From the provider perspective, the cost of transfer from inpatient to ambulatory treatment was $98, and that of transfer to another hospital inpatient setting was $217, per child treated (Table 5). This study, however, was conducted in 1994 and might not reflect the current treatment protocols.

### Study quality

Reporting quality was good for 45%, moderate for 45%, and low for 10% of studies (Table 3). There were nine areas that were most problematic. Only 14% of the included studies fully reported and justified the choice of discount rate(s) used for costs and outcomes. In addition, only 35% fully reported the dates of the estimated resource quantities and unit costs, methods for adjusting unit costs to the year of reported costs, or methods for converting costs into a common currency base and the exchange rate. The values, ranges, references, and probability distributions (where applicable) for all parameters and justification/ sources were fully reported by 39% of studies. Seventeen percent fully described all analytic methods supporting the evaluation. Thirty percent of studies fully characterised heterogeneity. Only 45% of analyses using single study–based estimates of effectiveness fully described the design features of the single effectiveness study used and why this was a sufficient source of clinical effectiveness data. 48% of studies reported the mean values for the main categories of estimated costs and outcomes of interest for each intervention, as well as mean differences between the comparator groups. Only 9% of single study–based economic evaluation fully describes the effects of sampling uncertainty and methodological assumption on the reported estimates. For model-based analyses, 44% fully described and justified their model choice.

### Economic evidence profiles

We identified very few cost analyses (ranging from 1 to 5), and no cost-effectiveness analyses at all for the different models of care for the management of growth failure/faltering in infants <12 months of age; management of moderate wasting in infants and children <60 months of age; and management of moderate wasting and severe wasting and/or bilateral pitting oedema together in infants and children <60 months of age (Table 7). There were no direct comparisons between different models of care. These issues raise very serious concerns, hence the certainty of evidence for these three groups was judged as very low overall according to the GRADE ratings.

**Table 7:** Economic profiles.

Intervention	Resource allocation considerations		Cost-effectiveness		Quality (Based on CHEERS)	Applicability	Certainty	Other limitations/ comments
	*Number of studies*	*Costs*	*Number of studies*	*Summary of results*				
**Management of growth failure/faltering in infants <12 months of age**								
*Transfer from inpatient to community treatment*	1	Transfer from inpatient to community treatment in a medical placement home was more expensive per child treated than treatment in an inpatient setting [159].	0	-	*Cost analysis*: Moderate	Directly applicable for <12 months age group Partially applicable for <6 months age group	No sensitivity analysis	No CEA studies comparing different settings
**Management of moderate wasting in infants and children <60 months of age**								
*Initiation of treatment in community settings*	5	No direct comparisons made with other settings [163, 165–167, 169].	0	-	*Cost analysis*: Moderate	Partially applicable: no direct comparisons with other settings	Sensitivity analysis only in one study but the study was evaluating increasing the amount of oil used in preparing corn-soy blend porridge in the same setting [169].	No CEA studies comparing different settings
*Initiation of treatment in outpatient settings*	2	No direct comparisons made with other settings [158, 168].	0	-	*Cost analysis*: Good	Partially applicable: no direct comparisons with other settings	Sensitivity analysis only in one study but the study evaluates different interventions in the same setting [158].	No CEA studies comparing different settings
**Management of severe wasting and/or bilateral pitting oedema in infants and children <60 months of age**								
*Initiation of treatment in community settings*	9	Costs per child treated/ recovered were lower overall when compared to outpatient [170–172] or hospital [160] treatment, or initiation in the community then referring to an inpatient setting [164].	1	Cost-effective when compared to outpatient settings using WHO’s GDP/capita threshold [174]	*Cost analyses*: Moderate overall (1 low; 3 moderate; 5 good) *CEA*: Good overall	Directly applicable	No sensitivity analysis performed	Cost comparisons with other settings based on only 5 studies. Cost-effectiveness results are based on one study and the uncertainty of the estimated were not explored.
*Initiation of treatment in outpatient settings*	17	Costs per child treated/ recovered were lower overall compared to treatment in inpatient settings [148, 149]; but higher when compared to treatment in community settings [170–172] or initiating treatment in outpatient settings then transferring to community settings [149].	6	Highly cost-effective when compared to a do nothing/ no programme scenario [147, 150, 154, 155, 164], using WHO’s GDP/capita threshold Uncertainty on which strategy is most cost-effective when compared to initiation of treatment in community settings [170].	*Cost analyses*: Moderate overall (1 low; 6 Moderate; 10 Good) *CEA*: Good overall	Directly applicable for cost analyses Partially applicable for CEA: comparisons were to a do nothing/ no programme scenario	Highly sensitive to projected number of deaths, costs of technical support, RUTF or to recover an additional child.	Cost comparisons with other settings based on only 5 studies. Five CEAs were accompanied by a PSA and/or one-/two-way sensitivity analysis [147, 150, 155, 164, 170]. However, two studies seem to only include scenarios that would favour outpatient settings [147, 155].
*Initiation of treatment in a combination of settings (community*, *outpatient and inpatient)*	3	Initiation of treatment in hospital and community settings costed more than initiation in community settings only [160].	0	-	*Cost analyses*: *Moderate overall (1 Low; 1 Moderate and 1 Good)*	Directly applicable	No sensitivity analysis performed	Cost comparisons with other settings only from one study. No CEA studies comparing different settings
*Referral to treatment in an inpatient setting*	4	Referral from community and outpatient settings was of lower cost per child treated/ recovered compared to outpatient treatment [167, 168]. Referral from community settings was however more expensive that community treatment alone [164].	1	Not cost-effective when compared to no treatment using WHO’s GPD/capita threshold [164].	*Cost-analyses*: Good overall (2 good; 2 moderate) *CEA*: Good	Directly applicable Partially applicable: for CEA as comparisons were to a no treatment scenario.	Highly sensitive to projected number of deaths.	CEA from one study.
*Transfer from outpatient treatment to treatment in a community setting*	1	Costs per child treated/ recovered were lower when compared to treatment in an outpatient or inpatient setting [149].	0	-	*Cost analysis*: Moderate	Directly applicable	No sensitivity analysis	No CEA studies comparing different settings.
**Management of moderate wasting and severe wasting and/or bilateral pitting oedema together in infants and children <60 months of age**								
*Initiation of treatment in outpatient settings*	2	One study made no direct comparisons made with other settings [152]. In one study, the daily societal costs per child recovered were lower for the 0–23 months age group, but higher for the 24 to 71 months age group, than for treatment initiation in community settings [175]. Daily household level costs per child treated were higher than treatment initiation in community settings [175].	0	-	*Cost analysis*: (1 good; 1 moderate)	Directly applicable: direct comparison with community settings in one study.	Sensitivity analysis performed in one study for costs with greatest uncertainty but not clear which ones and their impact [152].	No CEA studies comparing different settings
*Initiation of treatment in community settings*	1	In one study, the daily societal costs per child recovered were higher for the 0–23 months age group, but lower for the 24 to 71 months age group, than for treatment initiation in outpatient settings [175]. Daily household level costs per child treated were lower than treatment initiation in community settings [175].	0	-	*Cost analysis*: Moderate	Directly applicable	No sensitivity analysis	No CEA studies comparing different settings.
*Transfer from inpatient to outpatient*	1	the cost of transfer from inpatient to outpatient treatment was lower than that of transfer to another hospital inpatient setting, per child treated [153].	0	-	*Cost analysis*: Moderate	Directly applicable	Performed for loss to follow-up; per protocol and anthropometric outcomes. These analyses did not substantially differ from the main results.	No CEA studies comparing different settings

Evidence for the management of severe wasting and/or bilateral pitting oedema in infants and children <60 months of age suggests that costs for initiating treatment in community settings are the lowest, followed by initiating treatment in outpatient settings then transferring to community settings, then initiating treatment in outpatient settings, with initiating treatment in hospital settings being the most expensive. The certainty of the cost and cost-effectiveness evidence was, however, judged as very low for the following because only a few studies were identified, and there were no comparisons between different models of care: initiation of treatment in a combination of settings (3 cost and 0 cost-effectiveness studies); referral to treatment in an inpatient setting (4 cost and 1 cost-effectiveness studies); and transfer from outpatient treatment to treatment in a community setting (1 cost and 0 cost-effectiveness studies).

For initiation of treatment in community settings for severe wasting and/or bilateral pitting oedema in infants and children <60 months of age, 5 out of the 9 cost studies included comparisons with other settings. However, the cost-effectiveness results were based on one study and the uncertainty of the estimates were not explored. The certainty of the evidence was therefore judged as low. The certainty of the evidence for the initiation of treatment in outpatient settings for severe wasting and/or bilateral pitting oedema in infants and children <60 months of age was judged as moderate. There were 17 cost and 6 cost-effectiveness analyses. However, cost comparisons with other settings were based on only 5 studies, and for cost-effective analyses all comparisons were to a do nothing/ no programme scenario. This potentially limit the applicability of the evidence.

## Discussion

### Summary of principal findings

This review highlights glaring gaps in economic evidence to support decisions on models of care for growth failure/faltering in infants <12 months of age, and moderate wasting and severe wasting and/or bilateral pitting oedema in infants and children <60 months of age. The evidence remains inconclusive for most models of care. However, the evidence suggests that, for the treatment for severe wasting and/or bilateral pitting oedema in infants and children <60 months of age, the costs are lowest for initiating treatment in community settings, followed by initiating treatment in community and transferring to outpatient settings, initiating treatment in outpatients then transferring to community settings, initiating treatment in outpatient settings, and lastly initiating treatment in inpatient settings. Our findings also suggest that, for infants and children <60 months of age with severe wasting and/or bilateral pitting oedema, initiation of treatment in outpatient settings is highly cost-effective when compared to a do-nothing/ no programme scenario. This is in line with another review that also concluded that outpatient facility-based care for child wasting was highly cost-effective [10]. However, the strength of this evidence is limited due to a lack of comparisons with other alternatives.

Costs seem to vary widely within and across regions. For example, the initiation of treatment for severe wasting and/or bilateral pitting oedema in a community setting for those <60 months of age costs between $202 and $757 in Africa, and $206 to $7,987 in South-East Asia, per child treated from a provider perspective. Even within the same country, costs seem to vary widely. For example, the cost of initiating treatment for severe wasting and/or bilateral pitting oedema in outpatient settings in Mali varied between $103 and $1208 per child treated from a provider perspective. This suggests a strong influence of contextual factors that might limit the transferability of costs data from one setting to another [177]. Cost drivers that have been highlighted in this respect include price levels, population density, the scale of the programme, the underlying health of the population, existing health infrastructure, and implementer capacity [9, 10, 150, 173, 177]. For example, a programme situated in a population where the number of malnourished children is high is likely to experience lower costs per child treated or recovered than one in a population where these numbers are low [10]. This is because the costs, for example, indirect costs, will be divided among more children. A few of the studies included in our review performed sensitivity analysis and they reported that their results were highly sensitive to projected number of deaths, costs of technical support, RUTF or to recover an additional child [147, 150, 155, 164, 170].

Evidence on this topic is dominated by cost or cost-efficiency analyses, rather than cost-effectiveness analyses that can inform decisions on how to maximise outcomes and minimise opportunity costs. Many of the included studies also took a provider perspective despite undernutrition being a multisectoral problem resulting in resource consumption and impacting on outcomes in other sectors such as the education sector [178]. Community and household costs have been largely ignored: for example, only seven studies included productivity losses [161, 162, 165, 169–172], and four included transport costs incurred by families/caregivers [161, 165, 171, 172]. This is despite the fact that these costs can be high and potentially catastrophic, particularly for poor households, those without a reliable source of income, or where most of the costs are paid for out-of-pocket. In addition, these costs are very well-known barriers to accessing treatment and can differ substantially between different treatment models. For example, studies have suggested that community-based treatment for severe wasting and/or bilateral pitting oedema in children under 5 years of age can decrease household costs by six times when compared to inpatient treatment [164], and by three times compared to outpatient facility-based care [170, 171]. A wide range of outcomes were used, with inconsistencies in how they were defined, making comparisons across studies difficult.

### Strengths and limitations of the review

To our knowledge, this is the first comprehensive systematic review that focuses on the cost and cost-effectiveness of different models of care for the management of infants <12 months of age with growth faltering/failure, and infants and children aged <60 months of with moderate wasting and severe wasting and/or bilateral pitting oedema. We used a very comprehensive search strategy to identify both peer-reviewed journal articles and grey literature. We included all eligible study reports regardless of language, date of publication or the country in which the study was conducted. We also clearly defined our key variables of interest, i.e., population, setting and type of care, to ensure consistency and transparency in the way the studies were classified. All costs were converted to 2020 US dollar costs to facilitate comparisons across countries. Nevertheless, heterogeneity in perspectives of the evaluations, included costs, costing methods, outcomes and how these were defined and measured meant that we were not able to pool results or make comparisons across studies.

### Recommendations for policy and practice

Costs vary widely according to context and, therefore, the costs for the different models of care are also likely to vary greatly across countries. For most of the models of care explored in this systematic review, there is not enough cost-effectiveness evidence to inform recommendations for policy and practice. For severe wasting and/or bilateral pitting oedema in infants and children aged <60 months, initiation of treatment in outpatient settings can be recommended. There is no reason to believe that this will be different for the age group 6 to 59 months, which is the focus of the WHO recommendations for child wasting. However, most of the studies compared initiation of treatment in outpatient setting to do nothing/ no programme scenarios rather than to other settings or models of care. This may limit the applicability of these findings in real-world settings.

### Recommendations for future research

There is a need for further research on both the costs and cost-effectiveness of different treatment models for the management of growth failure/faltering in infants <12 months of age, and the management of moderate wasting and severe wasting and/or bilateral pitting oedema in infants and children aged <60 months. The research should compare the different alternatives. There is also a need for the standardisation of outcomes, including definitions and assessment methods. Some researchers have suggested that, as a minimum, studies should include the cost per child recovered [10]. This can be calculated using routine programme data. In addition, more comprehensive outcomes such as the DALY would allow comparisons with child health interventions: this is important for decision-makers. To be able to identify the main costs drivers, including contextual determinants, there is a need to develop a minimum set of costs that have to be included when conducting cost or cost-effectiveness analyses in this research area. Capturing the societal costs, rather than just the provider costs is also important due to the multi-sectoral nature of undernutrition [9, 10]. It will allow for decisions that account for the costs and cost savings to other sectors as well as the beneficiary’s households.

## Conclusions

There is very limited economic evidence to inform policy and practice on the setting of treatment initiation, referral, transfer and discharge of: 1) infants <12 months of age with growth faltering/failure; and 2) infants and children aged <60 months with moderate wasting. For infants and children aged <60 months with severe wasting and/or bilateral pitting oedema, evidence suggests that initiation of treatment in outpatient settings is highly cost-effective. However, the applicability of these findings in real-world settings could be limited as most of the comparisons are to do nothing/ no programme scenarios rather than to other settings or models of care.

## Supporting information

S1 File PRISMA checklist. (PDF)

S2 File Key definitions. (PDF)

S3 File Search strategies. (PDF)

S4 File Data extraction categories and variables. (PDF)

S5 File Characteristics of excluded studies. (PDF)

S6 File Study definitions for moderate wasting, severe wasting and/or bilateral pitting oedema, or growth failure/faltering and inclusion/exclusion criteria. (PDF)

S1 Table Cost analysis results for management of growth failure/faltering in infants <12 months of age. (DOCX)

S2 Table Cost analysis results for management of moderate wasting in infants and children <60 months of age. (DOCX)

S3 Table Cost-effectiveness analysis results for the management of moderate wasting in infants and children <60 months of age. (DOCX)

S4 Table Cost analysis results for the management of severe wasting and/or bilateral pitting oedema in infants and children <60 months of age. (DOCX)

S5 Table Cost-effectiveness analysis results for the management of severe wasting and/or bilateral pitting oedema in infants and children <60 months of age. (DOCX)

S6 Table Cost analysis results for the management of moderate wasting and severe wasting and/or bilateral pitting oedema together in infants and children <60 months of age. (DOCX)

## References

[1] United Nations Children’s Fund (UNICEF), World Health Organization, International Bank for Reconstruction and Development/The World Bank. Levels and trends in child malnutrition: key findings of the 2021 edition of the joint child malnutrition estimates. New York: United Nations Children’s Fund; 2021. https://www.who.int/publications/i/item/9789240025257

[2] WHO. Guideline: Update on the management of severe acute malnutrition in infants and children. Geneva: World Health Organization; 2013. https://iris.who.int/bitstream/handle/10665/95584/9789241506328_eng.pdf?sequence=1 24649519

[3] Black RE, Victora CG, Walker SP, Bhutta ZA, Christian P, de Onis M, et al. Maternal and child undernutrition and overweight in low-income and middle-income countries. Lancet. 2013;382:427–51. doi: 10.1016/S0140-6736(13)60937-X 23746772

[4] Fishman SM, Caulfield LE, De Onis M, Blossner M, Hyder AA, Mullany L, et al. Childhood and maternal underweight. In: Ezzati M, Lopez AD, Rodgers A, Murray CJL, editors. Comparative quantification of health risks: global and regional burden of disease attributable to selected major risk factors. 1. Geneva: World Health Organization; 2004. pp. 39–161. https://www.who.int/publications/i/item/9241580313

[5] Bain LE, Awah PK, Geraldine N, Kindong NP, Sigal Y, Bernard N, et al. Malnutrition in Sub-Saharan Africa: burden, causes and prospects. Pan Afr Med J. 2013;15:120. doi: 10.11604/pamj.2013.15.120.2535 24255726 PMC3830470

[6] Roth DE, Krishna A, Leung M, Shi J, Bassani DG, Barros AJD. Early childhood linear growth faltering in low-income and middle-income countries as a whole-population condition: analysis of 179 Demographic and Health Surveys from 64 countries (1993–2015). Lancet Glob Health. 2017;5:e1249–e57. doi: 10.1016/S2214-109X(17)30418-7 29132614 PMC5695758

[7] Victora CG, Christian P, Vidaletti LP, Gatica-Domínguez G, Menon P, Black RE. Revisiting maternal and child undernutrition in low-income and middle-income countries: variable progress towards an unfinished agenda. Lancet. 2021;397:1388–99. doi: 10.1016/S0140-6736(21)00394-9 33691094 PMC7613170

[8] Gonzalez-Viana E, Dworzynski K, Murphy MS, Peek R. Faltering growth in children: summary of NICE guidance. BMJ. 2017;358:j4219. doi: 10.1136/bmj.j4219 28963099

[9] Njuguna R, Berkley J, Jemutai J. Cost and cost-effectiveness analysis of treatment for child undernutrition in low- and middle-income countries: a systematic review [version 2; peer review: 2 approved]. Wellcome Open Research. 2020;5(62). doi: 10.12688/wellcomeopenres.15781.2 33102783 PMC7569484

[10] Chui J, Donnelly A, Cichon B, Mayberry A, Keane E. The cost-efficiency and cost-effectiveness of the management of wasting in children: A review of the evidence, approaches, and lessons. Save the Children UK; ACF, Action Against Hunger; 2020. https://resourcecentre.savethechildren.net/pdf/cea-report_final.pdf/

[11] Centre for Reviews and Dissemination. Systematic reviews: CRD’s guidance for undertaking reviews in health care. York: Centre for Reviews and Dissemination, University of York; 2008. https://www.york.ac.uk/media/crd/Systematic_Reviews.pdf

[12] Higgins JPT, Thomas J, Chandler J, Cumpston M, Li T, Page MJ, et al., editors. Cochrane handbook for systematic reviews of interventions version 6.2 (updated February 2021).: Cochrane; 2021.

[13] Mdege ND, Masuku SD, Musakwa N, Chisala M, Shokraneh F. Resource use, cost and cost-effectiveness of decisions on the setting of treatment initiation, referral, transfer and discharge of infants and children with moderate/severe wasting and oedema, and infants with growth failure/ faltering: protocol for a systematic review. York: University of York; 2021. https://pure-research.york.ac.uk/ws/portalfiles/portal/89637942/FINAL_PROTOCOL.pdf

[14] Ouzzani M, Hammady H, Fedorowicz Z, Elmagarmid A. Rayyan-a web and mobile app for systematic reviews. Syst Rev. 2016;5:210. doi: 10.1186/s13643-016-0384-4 27919275 PMC5139140

[15] Husereau D, Drummond M, Petrou S, Carswell C, Moher D, Greenberg D, et al. Consolidated Health Economic Evaluation Reporting Standards (CHEERS) statement. Value in Health. 2013;16:e1–5. doi: 10.1016/j.jval.2013.02.010 23538200

[16] Ryan R, Hill S. How to GRADE. Melbourne: Cochrane Consumers and Communication La Trobe University; 2018.

[17] National Institute for Health and Care Excellence. Developing NICE guidelines. 2022. https://www.nice.org.uk/process/pmg20/resources/developing-nice-guidelines-the-manual-pdf-72286708700869 26677490

[18] Brozek JL, Canelo-Aybar C, Akl EA, Bowen JM, Bucher J, Chiu WA, et al. GRADE Guidelines 30: the GRADE approach to assessing the certainty of modeled evidence-An overview in the context of health decision-making. J Clin Epidemiol. 2021;129:138–50. doi: 10.1016/j.jclinepi.2020.09.018 32980429 PMC8514123

[19] Shields GE, Elvidge J. Challenges in synthesising cost-effectiveness estimates. Sys Rev. 2020;9:289. doi: 10.1186/s13643-020-01536-x 33298168 PMC7727163

[20] Shemilt I MM, Byford S, Drummond M, Eisenstein E, Knapp M, Mallender J, et al. Chapter 15: Incorporating economics evidence. In: Higgins JPT, Green S, editors. Cochrane Handbook for Systematic Reviews of Interventions Version 510 (updated March 2011).: Cochrane; 2011.

[21] Liang BC. Chapter 2—Economics. In: Liang BC, editor. The pragmatic MBA for scientific and technical executives. San Diego: Academic Press; 2013. pp. 15–31.

[22] OECD Data. Purchasing power parities. n.d. https://data.oecd.org/conversion/purchasing-power-parities-ppp.htm

[23] International Monetary Fund. Inflation rate, average consumer prices 2022. https://www.imf.org/external/datamapper/PCPIPCH@WEO/OEMDC/ADVEC/WEOWORLD

[24] Gupta A, Kumari R. Community based intervention: potential impact of low cost nutrient–rich foods to alleviate malnutrition among children under 5 years of age. 2018. https://inis.iaea.org/collection/NCLCollectionStore/_Public/51/006/51006679.pdf?r=1

[25] Levinson FJ. Morinda: an economic analysis of malnutrition among young children in rural India. Cambridge, Mass: Cornell-MIT International Nutrition Policy Series; 1974.

[26] Lin YF, Lin CH, Lin YJ, Yeh TF. Outcome and cost of intensive care for very low birth weight infants. Zhonghua Minguo xiao er ke yi xue hui za zhi. 1995;36:266–70. 7572169

[27] Persson LA. Searching for cost-effective solutions to major nutrition problems in the world experiences from Bangladesh. Scand J Nutr. 1999;43:158–9.

[28] Radrigan ME, Atalah E, Fernandez E. Cost of recovery of the malnourished baby in special nutritional rehabilitation centers. Pediatria. 1979;22:122–5.

[29] Shirasaya K, Shimada N, Uemura T, Ikeda S, Miyakawa M, Kondo T. Cost benefit analysis of care of low-birth-weight infants. [Nihon koshu eisei zasshi] Japanese Journal of Public Health. 1995;42:783–91. 8534878

[30] Ssebunnya EE. Use of integrated management of acute malnutrition guidelines and cost implication in Uganda: a case study of severe acutely malnourished children admitted at Mwanamugimu Nutrition Unit, Mulago Hospital. 2013. http://dspace3.mak.ac.ug/handle/10570/4454?show=full

[31] Ahmed S, Sarma H, Hasan Z, Rahman M, Ahmed MW, Islam MA, et al. Cost-effectiveness of a market-based home fortification of food with micronutrient powder programme in Bangladesh. Public Health Nutr. 2021;24 (S1):s59–s70. doi: 10.1017/S1368980020003602 33118899 PMC8042576

[32] Altare C, Ait-Aissa M, Houngbe F, Kolsteren P, Tonguet-Papucci A, Huneau J-F, et al. Unconditional cash transfers do not prevent children’s undernutrition in themoderate acute malnutrition out (MAM’Out) cluster-randomized controlled trial in rural Burkina Faso. J Nutr. 2017;147:1410–7. doi: 10.3945/jn.117.247858 28539413

[33] Aye SKK, Mar SL, Lwin NN, Hnin ZL, Hlaing LM, Washington ML, et al. Stunting: an overlooked problem in Myanmar—an economic evaluation. Int J Technol Assess Health Care. 2020;36:167–72. doi: 10.1017/S0266462319003520 31955725

[34] Colombatti R, Vieira CS, Coin A, Bestagini P, Schiavon L, Ambrosini V, et al. A short-term intervention for the treatment of severe malnutrition in a post-conflict country: Results of a survey in Guinea Bissau. Public Health Nutr. 2008;11:1357–64. doi: 10.1017/S1368980008003297 18652716

[35] Delport D, Hainsworth S, Pearson R, Huang S, Toole M, Homer CS, et al. Ending malnutrition in all its forms requires scaling up proven nutrition interventions and much more: a 129-country analysis. BMC Med. 2020;18:356. doi: 10.1186/s12916-020-01786-5 33183301 PMC7661178

[36] Galasso E, Wagstaff A. The aggregate income losses from childhood stunting and the returns to a nutrition intervention aimed at reducing stunting. Econ Hum Biol. 2019;34:225–38. doi: 10.1016/j.ehb.2019.01.010 31003858

[37] Goudet S, Bogin B, Griffiths P, Jayaraman A, Chanani S, Osrin D, et al. Cost effectiveness of a community based prevention and treatment of acute malnutrition programme in Mumbai slums, India. PLoS ONE. 2018;13:e0205688. doi: 10.1371/journal.pone.0205688 30412636 PMC6226164

[38] Heckert J, Leroy JL, Olney DK, Ruel MT, Richter S, Iruhiriye E. The cost of improving nutritional outcomes through food-assisted maternal and child health and nutrition programmes in Burundi and Guatemala. Matern Child Nutr. 2020;16(1):e12863. doi: 10.1111/mcn.12863 31232512 PMC7038902

[39] Korte R. Operational aspects of different approaches to nutritional rehabilitation. Ecol Food Nutr. 1974;3:131–40. doi: 10.1080/03670244.1974.9990371

[40] Lakdawalla DN, Mascarenhas M, Jena AB, Vanderpuye-Orgle J, Lavallee C, Linthicum MT, et al. Impact of oral nutrition supplements on hospital outcomes in pediatric patients. JPEN J Parenter Enteral Nutr. 2014;38(2 Suppl):42S–9S. doi: 10.1177/0148607114549769 25233942

[41] Langendorf C, Roederer T, de Pee S, Brown D, Doyon S, Mamaty AA, et al. Preventing acute malnutrition among young children in crises: a prospective intervention study in Niger. PLoS Med. 2014;11:e1001714. doi: 10.1371/journal.pmed.1001714 25180584 PMC4152259

[42] Mason JB, Hay RW, Leresche J, Peel S, Darley S. Treatment of severe malnutrition in relief. Lancet. 1974;303:332–5. doi: 10.1016/S0140-6736(74)93077-3 4131171

[43] Melville B, Fidler T, Mehan D, Bernard E, Mullings J. Growth monitoring: the role of community health volunteers. Public Health. 1995;109:111–6. doi: 10.1016/s0033-3506(05)80004-6 7716251

[44] Neufeld LM, Garcia-Guerra A, Quezada AD, Theodore F, Bonvecchio Arenas A, Islas CD, et al. A fortified food can be replaced by micronutrient supplements for distribution in a Mexican social protection program based on results of a cluster-randomized trial and costing analysis. J Nutr. 2019;149:2302S–9S. doi: 10.1093/jn/nxz176 31793645 PMC6888020

[45] Puett C, Salpeteur C, Ait-Aissa M, Israel A-D, Lacroix E, Houngbe F. Protecting child health and nutrition status with ready-to-use food in addition to food assistance in urban Chad: a cost-effectiveness analysis. Cost Eff Resour Alloc. 2013;11:27. doi: 10.1186/1478-7547-11-27 24210058 PMC4176497

[46] Shen Y, Cliffer IR, Langlois BK, Webb P, Rogers BL, Suri DJ, et al. Impact of stakeholder perspectives on cost-effectiveness estimates of four specialized nutritious foods for preventing stunting and wasting in children 6–23 months in Burkina Faso. Nutr J. 2020;19:20. doi: 10.1186/s12937-020-00535-x 32106840 PMC7047349

[47] Tandon S, Arora B, Prasad M, Narula G, Banavali S, Gadge M, et al. A randomised controlled trial of ready to use therapeutic food (RUTF) for moderate/severe acute malnourished indian children with cancer. Arch Dis Child. 2018;103:A146‐A7. doi: 10.1136/archdischild-2018-rcpch.350

[48] Whittaker DE, Le Roux I, Disler P. The cost effectiveness of the Philani Nutrition Day Centre in Crossroads squatter camp, Cape Town. S Afr Med J. 1985;68:174–6. 3927496

[49] Akseer N, Tasic H, Onah MN, Wigle J, Rajakumar R, Sanchez-Hernandez D, et al. Economic costs of childhood stunting to the private sector in low-and middle-income countries. EClinicalMedicine. 2022;45:101320. doi: 10.1016/j.eclinm.2022.101320 35308896 PMC8927824

[50] Ale FGB, Phelan KPQ, Issa H, Defourny I, Le Duc G, Harczi G, et al. Mothers screening for malnutrition by mid-upper arm circumference is non-inferior to community health workers: results from a large-scale pragmatic trial in rural Niger. Arch Public Health. 2016;74:38. doi: 10.1186/s13690-016-0149-5 27602207 PMC5011948

[51] Assad M, Abraham JH, Elliott MJ. Decreased cost and improved feeding tolerance in VLBW infants fed an exclusive human milk diet. J Perinatol. 2016;36:216–20. doi: 10.1038/jp.2015.168 26562370

[52] Bergmann JN, Legins K, Sint TT, Snidal S, Group UR, Amor YB, et al. Outcomes and cost-effectiveness of integrating hiv and nutrition service delivery: pilots in Malawi and Mozambique. AIDS Behav. 2017;21:703–11. doi: 10.1007/s10461-016-1400-3 27094787

[53] Cobb G, Bland RM. Nutritional supplementation: The additional costs of managing children infected with HIV in resource-constrained settings. Trop Med Int Health. 2013;18:45–52. doi: 10.1111/tmi.12006 23107420 PMC3717178

[54] De Lauture H, Wone I, Perier-scheer M, Penot C. A model for combatting malnutrition in children: nutritional rehabilitation centers. J Famil Health Train. 1982;1:18–21. 12312106

[55] Thompson RT, Bennett WE Jr, Finnell SME, Downs SM, Carroll AE. Increased length of stay and costs associated with weekend admissions for failure to thrive. Pediatrics. 2013;131:e805–e10. doi: 10.1542/peds.2012-2015 23439903

[56] Griswold SP, Langlois BK, Shen Y, Cliffer IR, Suri DJ, Walton S, et al. Effectiveness and cost-effectiveness of 4 supplementary foods for treating moderate acute malnutrition: results from a cluster-randomized intervention trial in Sierra Leone. The Am J Clin Nutr. 2021;114(3):973–85. doi: 10.1093/ajcn/nqab140 34020452 PMC8408853

[57] Hossain MI, Dodd NS, Ahmed T, Miah GM, Jamil KM, Nahar B, et al. Experience in managing severe malnutrition in a government tertiary treatment facility in Bangladesh. J Health Popul Nutr. 2009;27:72–9. doi: 10.3329/jhpn.v27i1.3319 19248650 PMC2761803

[58] Seigel JK, Tanaka DT, Goldberg RN, Smith PB, Cotten CM, Bidegain M. Economic impact of human milk on medical charges of extremely low birth weight infants. Breastfeed Med. 2014;9:233–4. doi: 10.1089/bfm.2013.0059 24491037

[59] Yang Y, Van den Broeck J, Wein LM. Ready-to-use food-allocation policy to reduce the effects of childhood undernutrition in developing countries. Proc Natl Acad Sci U S A. 2013;110:4545–50. doi: 10.1073/pnas.1216075110 23487755 PMC3606988

[60] Laillou A, Baye K, Meseret Z, Darsene H, Rashid A, Chitekwe S. Wasted children and wasted time: A challenge to meeting the nutrition sustainable development goals with a high economic impact to ethiopia. Nutrients. 2020;12:3698. doi: 10.3390/nu12123698 33266008 PMC7760409

[61] Lima G, Quintero-Romero S, Cattaneo A. Feasibility, acceptability and cost of kangaroo mother care in Recife, Brazil. Ann Trop Paediatr. 2000;20:22–6. doi: 10.1080/02724930092020 10824209

[62] Lukaczer M. Lessons for the federal effort against hunger and malnutrition—from a case study. Am J Public Health. 1971;61:259–76. doi: 10.2105/ajph.61.2.259 5100332 PMC1530670

[63] Malan AF, Ryan E, Van Der Elst CW, Pelteret R. The cost of neonatal care. S Afr Med J. 1992;82(6):417–9. 1465692

[64] Marino LV, Meyer R, Cooke ML. Cost comparison between powdered versus energy dense infant formula for undernourished children in a hospital setting. e-SPEN Journal. 2013;8(4):e145–e9. doi: 10.1016/J.CLNME.2013.04.002

[65] Nordermoen A, Bratlid D. Costs for treatment of very-low-birth-weight infants. Tidsskr Nor Laegeforen. 2010;130:1130–4. doi: 10.4045/tidsskr.09.0378 20531498

[66] Parker LA, Krueger C, Sullivan S, Kelechi T, Mueller M. Effect of breast milk on hospital costs and length of stay among very low-birth-weight infants in the NICU. Adv Neonatal Care. 2012;12:254–9. doi: 10.1097/ANC.0b013e318260921a 22864006

[67] Peabody JW, Quimbo S, Florentino J, Shimkhada R, Javier X, Paculdo D, et al. Comparative effectiveness of two disparate policies on child health: experimental evidence from the Philippines. Health Policy Plan. 2017;32:563–71. doi: 10.1093/heapol/czw179 28110265 PMC5400045

[68] Rice SJC, Craig D, McCormick F, Renfrew MJ, Williams AF. Economic evaluation of enhanced staff contact for the promotion of breastfeeding for low birth weight infants. Int J Technol Assess Health Care. 2010;26:133–40. doi: 10.1017/S0266462310000115 20392315

[69] Russell RB, Howse JL, Poschman K, Dias T, Potetz L, Davidoff MJ, et al. Cost of hospitalization for preterm and low birth weight infants in the United States. Pediatrics. 2007;120:e1–e9. doi: 10.1542/peds.2006-2386 17606536

[70] Scholz SM, Greiner W. An exclusive human milk diet for very low birth weight newborns-A cost-effectiveness and EVPI study for Germany. PLoS ONE. 2019;14(12):e0226496. doi: 10.1371/journal.pone.0226496 31887150 PMC6936873

[71] Stevenson RC, Pharoah POD, Cooke RWI, Sandhu B. Predicting costs and outcomes of neonatal intensive care for very low birthweight infants. Public Health. 1991;105(2):121–6. doi: 10.1016/s0033-3506(05)80285-9 1906187

[72] Tahir W, Monahan M, Dorling J, Hewer O, Bowler U, Linsell L, et al. Economic evaluation alongside the Speed of Increasing milk Feeds Trial (SIFT). Arch Dis Child Fetal Neonatal Ed. 2020;105:587–92. doi: 10.1136/archdischild-2019-318346 32241810 PMC7592357

[73] Taylor C, Buckle A, Lilford R, Joolay Y. Prioritising allocation of donor human breast milk amongst very low birthweight infants in middle-income countries. Matern Child Nutr. 2018;14:e12595. doi: 10.1111/mcn.12595 30592164 PMC6865934

[74] Thanh NX, Toye J, Savu A, Kumar M, Kaul P. Health service use and costs associated with low birth weight—a population level analysis. J Pediatr. 2015;167:551–3. doi: 10.1016/j.jpeds.2015.06.007 26148659

[75] Tommiska V, Tuominen R, Fellman V. Economic costs of care in extremely low birthweight infants during the first 2 years of life. Pediatr Crit Care Med. 2003;4:157–63. doi: 10.1097/01.PCC.0000059731.74435.02 12749645

[76] Tongo OO, Orimadegun AE, Ajayi SO, Akinyinka OO. The economic burden of preterm/very low birth weight care in Nigeria. J Trop Pediatr. 2009;55:262–4. doi: 10.1093/tropej/fmn107 19066170

[77] Trang S, Zupancic JAF, Unger S, Kiss A, Bando N, Wong S, et al. Cost-effectiveness of supplemental donor milk versus formula for very low birth weight infants. Pediatrics. 2018;141:e20170737. doi: 10.1542/peds.2017-0737 29490909

[78] Tudehope DI, Lee W, Harris F, Addison C. Cost-analysis of neonatal intensive and special care. Aust Paediatr J. 1989;25:61–5. 2735885

[79] Vahidi RG, Jannati A, Gholipour K, Ghoddoosi-Nejad J, Bayan H, Hosseini MB. Cost and effectiveness analysis of Kangaroo mother care and conventional care method in low birth weight neonates in Tabriz 2010–2011. J Clin Neonatol. 2014;3:148–52. doi: 10.4103/2249-4847.140401

[80] Daga S, Verma B, Shahane S, Janged S, Vachagan MM. Syndromic management of common illnesses in hospitalized children and neonates: a cost identification study. Indian Journal of Pediatrics. 2010;77:1383–6. doi: 10.1007/s12098-010-0172-4 20844991

[81] Walker DJB, Vohr BR, Oh W. Economic analysis of regionalized neonatal care for very low-birth-weight infants in the state of Rhode Island. Pediatrics. 1985;76:69–74. 3925429

[82] Westrupp EM, Lucas N, Mensah FK, Gold L, Wake M, Nicholson JM. Community-based healthcare costs for children born low birthweight, preterm and/or small for gestational age: data from the Longitudinal Study of Australian Children. Child Care Health Dev. 2014;40:259–66. doi: 10.1111/cch.12040 23461342

[83] Wong B, Radin M. Benefit-cost analysis of a package of early childhood interventions to improve nutrition in Haiti. J Benefit Cost Anal. 2019;10:154–84. doi: 10.1017/bca.2019.1 32968617 PMC7473066

[84] Wynn A, Rotheram-Borus MJ, Weichle T, Leibowitz AA, Roux I le, Tomlinson M. Mentor mothers program improved child health outcomes at a relatively low cost in South Africa. Health Aff (Millwood). 2017;36:1947–55. doi: 10.1377/hlthaff.2017.0553 29137500 PMC6382468

[85] Zoungrana B, Somda NS, Tapsoba F, Tankoano A, Savadogo A, Sawadogo PS. Effectiveness and cost of management of severe acute malnutrition with complications in kaya, Burkina Faso. Pan Afr Med J. 2019;34:145. doi: 10.11604/pamj.2019.34.145.17946 32110264 PMC7024140

[86] Sharma D, Murki S, Pratap OT. To compare growth outcomes and cost-effectiveness of "Kangaroo ward care" with "intermediate intensive care" in stable extremely low birth weight infants: randomized control trial. J Matern Fetal Neonatal Med. 2017;30:1659–65. doi: 10.1080/14767058.2016.1220531 27492145

[87] Guitti JCS, Udihara IY, Nishitani H. Cost of hospital treatment for children with severe protein calorie malnutrition. Jornal de Pediatria. 1979;47:99–104.

[88] Ackatia-Armah RS, McDonald CM, Doumbia S, Erhardt JG, Hamer DH, Brown KH. Malian children with moderate acute malnutrition who are treated with lipid-based dietary supplements have greater weight gains and recovery rates than those treated with locally produced cereal-legume products: A community-based, cluster-randomized trial. Am J Clin Nutr. 2015;101:632–45. doi: 10.3945/ajcn.113.069807 25733649

[89] Aguayo VM, Jacob S, Badgaiyan N, Chandra P, Kumar A, Singh K. Providing care for children with severe acute malnutrition in India: new evidence from Jharkhand. Public Health Nutr. 2014;17:206–11. doi: 10.1017/S1368980012004788 23137752 PMC10282323

[90] Greco L, Balungi J, Amono K, Iriso R, Corrado B. Effect of a low-cost food on the recovery and death rate of malnourished children. J Pediatr Gastroenterol Nutr. 2006;43:512–7. doi: 10.1097/01.mpg.0000239740.17606.49 17033528

[91] Binns PJ, Dale NM, Banda T, Banda C, Shaba B, Myatt M. Safety and practicability of using mid-upper arm circumference as a discharge criterion in community based management of severe acute malnutrition in children aged 6 to 59 months programmes. Arch Public Health. 2016;74:24. doi: 10.1186/s13690-016-0136-x 27307989 PMC4908708

[92] Bredow MT, Jackson AA. Community based, effective, low cost approach to the treatment of severe malnutrition in rural Jamaica. Arch Dis Child. 1994;71:297–303. doi: 10.1136/adc.71.4.297 7979520 PMC1030005

[93] Coppieters Y, Parent F. Assessment of hospital morbidity, mortality, and cost-effectiveness of a nutritional program for children under 5 years of age in Pala, Chad. J Trop Pediatr. 2000;46:252–4. doi: 10.1093/tropej/46.4.252 10996995

[94] Fink G, Levenson R, Tembo S, Rockers PC. Home- and community-based growth monitoring to reduce early life growth faltering: an open-label, cluster-randomized controlled trial. Am J Clin Nutr. 2017;106:1070–7. doi: 10.3945/ajcn.117.157545 28835364 PMC5611784

[95] Griswold S, Langlois B, Shen Y, Cliffer I, Singh A, Potani I, et al. Comparative cost-effectiveness of four supplementary foods in treating moderate acute malnutrition in children 6–59 months in Sierra Leone, report to USAID from the Food Aid Quality Review. Boston, MA: Tufts University; 2019.

[96] Korachais C, Meessen B, Nkurunziza S, Nimpagaritse M. Impact of the extension of a performance-based financing scheme to nutrition services in Burundi on malnutrition prevention and management among children below five: a cluster-randomized control trial. PLoS ONE. 2020;15:e0239036. doi: 10.1371/journal.pone.0239036 32946500 PMC7500612

[97] Kozuki N, Van Boetzelaer E, Tesfai C, Zhou A. Severe acute malnutrition treatment delivered by low-literate community health workers in South Sudan: a prospective cohort study. J Glob Health. 2020;10:010421. doi: 10.7189/jogh.10.010421 32566163 PMC7295452

[98] Lagrone L, Cole S, Schondelmeyer A, Maleta K, Manary MJ. Locally produced ready-to-use supplementary food is an effective treatment of moderate acute malnutrition in an operational setting. Ann Trop Paediatr. 2010;30:103–8. doi: 10.1179/146532810X12703901870651 20522296

[99] Lake A. A tipping point for child survival, health, and nutrition. Lancet. 2012;380:1286–7. doi: 10.1016/S0140-6736(12)61539-6 22999429

[100] Lansot B. Economic and social factors in infant malnutrition. Study of a sample of 200 families of children admitted to the" Dr. Pedro Visca" hospital, August-September 1961. Arch Pediat Uruguay. 1963;34:143–7.

[101] Laugesen BM. A weight chart and weighing scale for nutrition surveys and grading of malnutrition in clinics. Indian Pediatr. 1974;11:285–8. 4213981

[102] Hummer M, Lehner T, Pruckner G. Low birth weight and health expenditures from birth to late adolescence. Eur J Health Econ. 2014;15:229–42. doi: 10.1007/s10198-013-0468-1 23546738

[103] Leroy J, Olney D. The evaluation of tubaramure in Burundi: an integrated food aid program. Ann Nutr Metab. 2013;63:37.

[104] Leveno KJ, Cunningham FG, Roark ML. Prenatal care and the low birth weight infant. Obstetrics & Gynecology. 1985;66:599–605. 4058817

[105] Lewit EM, Baker LS, Corman H, Shiono PH. The direct cost of low birth weight. The Future Child. 1995;5:35–56. 7633866

[106] Lim G, Tracey J, Boom N, Karmakar S, Wang J, Berthelot J-M, et al. CIHI survey: Hospital costs for preterm and small-for-gestational age babies in Canada. Healthc Q. 2009;12:20–4. doi: 10.12927/hcq.2013.21121 20057225

[107] Magnin M, Stoll B, Jeannot E, Voahangy R. A realistic evaluation approach highlighted the success factors and difficulties of an innovative and comprehensive malnutrition programme in Madagascar. Acta Paediatr. 2018;107:1570–80. doi: 10.1111/apa.14267 29424058

[108] Marini A. Three essays on economic determinants of child malnutrition. New York: Cornell University; 2004.

[109] Marzouk A, Filipovic-Pierucci A, Baud O, Tsatsaris V, Ego A, Charles M-A, et al. Prenatal and post-natal cost of small for gestational age infants: a national study. BMC Health Serv Res. 2017;17:221. doi: 10.1186/s12913-017-2155-x 28320392 PMC5359886

[110] Meregaglia M, Croci I, Brusco C, Herich LC, Di Lallo D, Gargano G, et al. Low socio-economic conditions and prematurity-related morbidities explain healthcare use and costs for 2-year-old very preterm children. Acta Paediatr. 2020;109:1791–800. doi: 10.1111/apa.15183 31977107

[111] Moench-Pfanner R, Bloem MW. ASEAN: insights and considerations toward nutrition programs. Food Nutr Bull. 2013;34:S4–7. doi: 10.1177/15648265130342S102 24049991

[112] Moench-Pfanner R, Silo S, Poirot E, Laillou A, Hong R, Wieringa F, et al. The economic burden of malnutrition in pregnant women and children under 5 years of age in Cambodia. Nutrients. 2016;8:292. doi: 10.3390/nu8050292 27187462 PMC4882705

[113] Newman NM. The very low birthweight infant—what cost? Med J Aust. 1986;145:121–2. doi: 10.5694/j.1326-5377.1986.tb113765.x 3736474

[114] Norgaard SK, Vissing NH, Chawes BL, Stokholm J, Bonnelykke K, Bisgaard H. Cost of illness in young children: a prospective birth cohort study. Children (Basel). 2021;8:173. doi: 10.3390/children8030173 33668336 PMC7996350

[115] Owino VO, Irena AH, Dibari F, Collins S. Development and acceptability of a novel milk-free soybean-maize-sorghum ready-to-use therapeutic food (SMS-RUTF) based on industrial extrusion cooking process. Matern Child Nutr. 2014;10:126–34. doi: 10.1111/j.1740-8709.2012.00400.x 22462436 PMC6860201

[116] Beam AL, Fried I, Palmer N, Agniel D, Brat G, Fox K, et al. Estimates of healthcare spending for preterm and low-birthweight infants in a commercially insured population: 2008–2016. Obstet Gynecol Surv. 2020;75:717–8. doi: 10.1097/OGX.0000000000000878 PMC7314662 32103158

[117] Raketa Ella CW, Ousmane O, Augustin NP, Mamoudou DH, Tiatou S, Kou’santa Emile AS, et al. Evaluation of the effectiveness of cost-free nutrition programme on children in reo health district, Burkina Faso. International Journal of Pharmaceutical Research and Allied Sciences. 2020;9:24–33.

[118] Rashid MA, Rahman ME, Kamruzzaman M, Islam MS, Moniruzzaman MM, Sabiha K, et al. Efficacy of F-75 & F-100 recipes in thetreatment of severe acute malnutrition: a randomized controlled trial. Mymensingh Med J. 2019;28:887–93. 31599256

[119] Rogowski J. Cost-effectiveness of care for very low birth weight infants. Pediatrics. 1998;102:35–43. doi: 10.1542/peds.102.1.35 9651411

[120] Sandhu B, Stevenson RC, Cooke RWI, Pharoah POD. Cost of neonatal intensive care for very-low-birthweight infants. Lancet. 1986;327:600–3. doi: 10.1016/s0140-6736(86)92820-5 2869312

[121] Segre J, Liu G, Komrska J. Local versus offshore production of ready-to-use therapeutic foods and small quantity lipid-based nutrient supplements. Matern Child Nutr. 2017;13:e12376. doi: 10.1111/mcn.12376 27863004 PMC6866252

[122] Shen YK, Griswold S, Suri D, Vosti SA, Rogers B. Costing methods for a cluster-randomized cost-effectiveness trial comparing the performance of four supplementary foods in treating Sierra Leonean children with moderate acute malnutrition (MAM). FASEB journal. 2017;31:786.55–786.55. doi: 10.1096/fasebj.31.1_supplement.786.55

[123] Steenson RC, McCabe CJ, Pharoah POD, Cooke RWI. Cost of care for a geographically determined population of low birthweight infants to age 8–9 years. I. Children without disability. Arch Dis Child Fetal Neonatal Ed. 1996;74:F114–F7. doi: 10.1136/fn.74.2.f114 8777657 PMC2528521

[124] Stevenson RC, Pharoah POD, Stevenson CJ, McCabe CJ, Cooke RWI. Cost of care for a geographically determined population of low birthweight infants to age 8–9 years. II. Children with disability. Arch Dis Child Fetal Neonatal Ed. 1996;74:F118–F21. doi: 10.1136/fn.74.2.f118 8777658 PMC2528526

[125] Wanzira H. Supportive Supervision as an approach to improve the quality of care for children with acute malnutrition in Arua district, Uganda: baseline systematic assessment, cluster randomised controlled trial and cost-effectiveness analysis.: Università degli Studi di Trieste; 2019. https://arts.units.it/bitstream/11368/2962380/2/Final revised PhD Thesis.pdf

[126] Yu VYH, Bajuk B. Medical expenses of neonatal intensive care for very low birthweight infants. Austr Paediatr J. 1981;17:183–5. doi: 10.1111/j.1440-1754.1981.tb01935.x 7325899

[127] Ashworth A. Efficacy and effectiveness of community-based treatment of severe malnutrition. Food Nutr Bull. 2006;27(3 Suppl):S24–48. doi: 10.1177/15648265060273S303 17076212

[128] Ayokunle O, Odusoga AO. Community-based management of micronutrient deficiency in malnourished children in Ghana. Bangladesh Journal of Medical Science. 2014;13(4):383–7. doi: 10.3329/bjms.v13i4.20552

[129] Bachmann MO. Cost-effectiveness of community-based treatment of severe acute malnutrition in children. Expert Rev Pharmacoecon Outcomes Res. 2010;10:605–12. doi: 10.1586/erp.10.54 20950075

[130] Bhutta ZA, Das JK, Rizvi A, Gaffey MF, Walker N, Black RE, et al. Evidence-based interventions for improvement of maternal and child nutrition: what can be done and at what cost? Lancet. 2013;382:452–77. doi: 10.1016/S0140-6736(13)60996-4 23746776

[131] Collins S. Treating severe acute malnutrition seriously. Arch Dis Child. 2007;92:453–61. doi: 10.1136/adc.2006.098327 17449529 PMC2083726

[132] Collins S, Sadler K, Dent N, Khara T, Guerrero S, Myatt M, et al. Key issues in the success of community-based management of severe malnutrition. Food Nutr Bull. 2006;27:S49–82. doi: 10.1177/15648265060273S304 17076213

[133] Horton S. Unit costs, cost—effectiveness, and financing of nutrition interventions. Unlisted: The World Bank, Policy Research Working Paper Series;1992. https://documents1.worldbank.org/curated/en/788981468741317611/pdf/multi0page.pdf

[134] Manary M, Callaghan-Gillespie M. Role of optimized plant protein combinations as a low-cost alternative to dairy ingredients in foods for prevention and treatment of moderate acute malnutrition and severe acute malnutrition. Nestle Nutr Inst Workshop Ser. 2020;93:111–20. doi: 10.1159/000503347 31991424

[135] McCamish MA. Malnutrition and nutrition support interventions: cost, benefits, and outcomes. Nutrition. 1993;9:556–7. 8111148

[136] McLachlan M. Tackling the child malnutrition problem: From what and why to how much and how. J Pediatr Gastroenterol Nutr. 2006;43:S38–S46. doi: 10.1097/01.mpg.0000255849.22777.69 17204977

[137] Mizumoto K, Murakami G, Oshidari K, Trisnantoro L, Yoshiike N. Health economics of nutrition intervention in asia: cost of malnutrition. J Nutr Sci Vitaminol (Tokyo). 2015;61:S47–9. doi: 10.3177/jnsv.61.S47 26598883

[138] Popkin BM. Some economic aspects of planning health interventions among malnourished populations. Am J Clin Nutr. 1978;31:2314–23. doi: 10.1093/ajcn/31.12.2314 103426

[139] Schofield WN. Treatment of malnutrition. Lancet. 1995;345:787–8. doi: 10.1016/s0140-6736(95)90665-7 7741914

[140] Suri DJ, Moorthy D, Rosenberg IH. The role of dairy in the comparative effectiveness and cost of fortified blended foods versus ready‐to‐use foods in treatment of children with moderate acute malnutrition. The FASEB Journal. 2016;30:669.14–.14.

[141] Ticas JM. [Food mixtures of high nutritional value and low cost in the fight against protein-calorie malnutrition]. Las mezclas alimenticias de alto valor nutritivo y bajo costo en la lucha contra la desnutricion proteino-calorica. Bol Oficina Sanit Panam.1978;85:26–39. 150844

[142] Huybregts L, Becquey E, Zongrone A, Le Port A, Khassanova R, Coulibaly L, et al. The impact of integrated prevention and treatment on child malnutrition and health: the PROMIS project, a randomized control trial in Burkina Faso and Mali. BMC Public Health. 2017;17:237. doi: 10.1186/s12889-017-4146-6 28274214 PMC5343313

[143] Levitt C, Watters N, Chance G, Walker R, Avard D. Low-birth-weight symposium: summary of proceedings. CMAJ: Canadian Medical Association journal [Journal de l’Association Medicale Canadienne]. 1993;148:767–71. 8439935 PMC1490579

[144] Mason J, Hunt J, Parker D, Jonsson U. Investing in child nutrition in Asia. Asian Development Review. 1999;17:1–32. doi: 10.1142/S0116110599000019

[145] Mehl AL, Doyle LW. The cost-benefit threshold for low birth weight infants [1] (multiple letters). Pediatrics. 2004;114:508. doi: 10.1542/peds.114.2.508 15286243

[146] Akram D-S, Suleman Y, Hanif HM. Home-based rehabilitation of severely malnourished children using indigenous high-density diet. J Pak Med Assoc. 2016;66(3):251–5. 26968271

[147] Ali S, Vargas P, Keen S. Cost-effectiveness of the WINNN programme: operations research and impact evaluation. Oxford: Operations Research and Impact Evaluation (ORIE); 2017. https://core.ac.uk/download/pdf/237086429.pdf

[148] Ashraf H, Alam NH, Sultana M, Jahan SA, Begum N, Farzana S, et al. Day clinic vs. hospital care of pneumonia and severe malnutrition in children under five: a randomised trial. Trop Med Int Health. 2019;24:922–31. doi: 10.1111/tmi.13242 31046165

[149] Ashworth A, Khanum S. Cost-effective treatment for severely malnourished children: what is the best approach? Health Policy Plan. 1997;12:115–21. doi: 10.1093/heapol/12.2.115 10168194

[150] Bachmann MO. Cost effectiveness of community-based therapeutic care for children with severe acute malnutrition in Zambia: decision tree model. Cost Eff Resour Alloc. 2009;7:2. doi: 10.1186/1478-7547-7-2 19146668 PMC2630929

[151] Bai KI. Teaching better nutrition by domiciliary management of cases of protein calorie malnutrition in rural areas (a longitudinal study of clinical and economical aspects). J Trop Pediatr Environ Child Health. 1972;18:307–12. 4209738

[152] Bailey J, Opondo C, Lelijveld N, Marron B, Onyo P, Musyoki EN, et al. A simplified, combined protocol versus standard treatment for acute malnutrition in children 6–59 months (ComPAS trial): a cluster-randomized controlled non-inferiority trial in Kenya and South Sudan. PLoS Med. 2020;17:e1003192–e. doi: 10.1371/journal.pmed.1003192 32645109 PMC7347103

[153] Chapko MK, Prual A, Gamatie Y, Maazou AA. Randomized clinical trial comparing hospital to ambulatory rehabilitation of malnourished children in Niger. J Trop Pediatr. 1994;40:225–30. doi: 10.1093/tropej/40.4.225 7932936

[154] Fotso C, Myatt M. Cost effectiveness analysis of the community-based management of acute malnutrition (CMAM) surge approach: Ethiopia. EVIHDAF, CONCERN worldwide; 2019. https://admin.concern.org.uk/sites/default/files/documents/2020-11/CEA%20CMAM%20Niger%20Final%20Report%20July%2018%202019_14.pdf

[155] Frankel S, Roland M, Makinen M. Costs, cost-effectiveness, and financial sustainability of community-based management of acute malnutrition in northern Nigeria. Washington DC: Results for Development Institute; 2015. https://ciff.org/wp-content/uploads/2019/09/R4D_CMAM_CostEffectiveness_FinancialSustainability_evaluation.pdf

[156] Garg CC, Mazumder S, Taneja S, Shekhar M, Mohan SB, Bose A, et al. Costing of three feeding regimens for home-based management of children with uncomplicated severe acute malnutrition from a randomised trial in India. BMJ Global Health. 2018;3:e000702. doi: 10.1136/bmjgh-2017-000702 29527358 PMC5841493

[157] International Rescue Committee. Cost efficiency analysis: treating severe acute malnutrition. International Rescue Committee; 2016. https://www.rescue.org/sites/default/files/document/959/nutritiondesignedbrieffinal.pdf

[158] Isanaka S, Barnhart DA, McDonald CM, Ackatia-Armah RS, Kupka R, Doumbia S, et al. Cost-effectiveness of community-based screening and treatment of moderate acute malnutrition in Mali. BMJ Glob Health. 2019;4:e001227. doi: 10.1136/bmjgh-2018-001227 31139441 PMC6509694

[159] Karniski W, Van Buren L, Cupoli JM. A treatment program for failure to thrive: a cost/effectiveness analysis. Child Abuse Negl. 1986;10:471–8. doi: 10.1016/0145-2134(86)90051-7 3098356

[160] Isanaka S, Menzies NA, Sayyad J, Ayoola M, Grais RF, Doyon S. Cost analysis of the treatment of severe acute malnutrition in West Africa. Matern Child Nutr. 2017;13: e12398. doi: 10.1111/mcn.12398 27921381 PMC6866029

[161] Masiiwa R. Inpatient household economic burden of child malnutrition in Zimbabwe: a case study conducted at Harare Central hospital: Cape Town: University of Cape Town; 2013. https://open.uct.ac.za/server/api/core/bitstreams/d04cda85-6ab8-415a-b5dc-c8ebd7780d85/content

[162] N’Diaye DS, Wassonguema B, Salpeteur C, Nikiema V, Kangas ST. Economic evaluation of a reduced dosage of ready-to-use therapeutic foods to treat uncomplicated severe acute malnourished children aged 6–59 months in Burkina Faso. Matern Child Nutr. 2021;17:e13118. doi: 10.1111/mcn.13118 33621428 PMC8189238

[163] Nkonki LLL, Chola LL, Tugendhaft AA, Hofman KK. Modelling the cost of community interventions to reduce child mortality in South Africa using the Lives Saved Tool (LiST). BMJ Open. 2017;7:e011425. doi: 10.1136/bmjopen-2016-011425 28851766 PMC5577872

[164] Puett C, Sadler K, Alderman H, Coates J, Fiedler JL, Myatt M. Cost-effectiveness of the community-based management of severe acute malnutrition by community health workers in southern Bangladesh. Health Policy Plan. 2013;28:386–99. doi: 10.1093/heapol/czs070 22879522

[165] Purwestri RC, Scherbaum V, Inayati DA, Wirawan NN, Suryantan J, Bloem MA, et al. Cost analysis of community-based daily and weekly programs for treatment of moderate and mild wasting among children on Nias Island, Indonesia. Food Nutr Bull. 2012;33:207–16. doi: 10.1177/156482651203300306 23156124

[166] Reed S, Kouam EC, Chhetri D, Subedi PK, Sapkota P, Sigdel U. Evaluation of community management of acute malnutrition (CMAM): Nepal country case study. New York: United Nations Children’s Fund (UNICEF); 2012c. https://assets.publishing.service.gov.uk/media/57a08a6140f0b64974000584/60997-Nepal-CMAM-evaluation-UNICEF-11oct2012.pdf

[167] Reed S, Kouam EC, Njoroge L, Momanyi C, Okuku HS, Onyancha G. Evaluation of integrated management of acute malnutrition (IMAM): Kenya country case study. New York: United Nations Children’s Fund; 2012b. https://evaluationreports.unicef.org/GetDocument?fileID=2997

[168] Reed S, Kouam EC, Paracha P, Din Z-U, Ullah N, Saeed A, et al. Evaluation of community management of acute malnutrition (CMAM): Pakistan country case study. New York: United Nations Children’s Fund (UNICEF); 2012a. https://evaluationreports.unicef.org/GetDocument?fileID=2998

[169] Rogers BL, Wilner LB, Maganga G, Walton SM, Langlois BK, Boiteau JM, et al. Program changes are effective and cost-effective in increasing the amount of oil used in preparing corn soy blend porridge for treatment of moderate acute malnutrition in Malawi. Matern Child Nutr. 2017;13:e12393. doi: 10.1111/mcn.12393 28083927 PMC6866085

[170] Rogers E, Guerrero S, Kumar D, Soofi S, Fazal S, Martinez K, et al. Evaluation of the cost-effectiveness of the treatment of uncomplicated severe acute malnutrition by lady health workers as compared to an outpatient therapeutic feeding programme in Sindh Province, Pakistan. BMC Public Health. 2019;19:84. doi: 10.1186/s12889-018-6382-9 30654780 PMC6337795

[171] Rogers E, Martinez K, Moran JLA, Ale FGB, Charle P, Guerrero S, et al. Cost-effectiveness of the treatment of uncomplicated severe acute malnutrition by community health workers compared to treatment provided at an outpatient facility in rural Mali. Hum Resour Health. 2018;16:12. doi: 10.1186/s12960-018-0273-0 29458382 PMC5819265

[172] Tekeste A, Wondafrash M, Azene G, Deribe K. Cost effectiveness of community-based and in-patient therapeutic feeding programs to treat severe acute malnutrition in Ethiopia. Cost Eff Resour Alloc. 2012;10:4. doi: 10.1186/1478-7547-10-4 22429892 PMC3323427

[173] Wilford R, Golden K, Walker DG. Cost-effectiveness of community-based management of acute malnutrition in Malawi. Health Policy Plan. 2012;27:127–37. doi: 10.1093/heapol/czr017 21378101

[174] Wilunda C, Mumba FG, Putoto G, Maya G, Musa E, Lorusso V, et al. Effectiveness of screening and treatment of children with severe acute malnutrition by community health workers in Simiyu region, Tanzania: a quasi-experimental pilot study. Sci Rep. 2021;11:2342. doi: 10.1038/s41598-021-81811-6 33504865 PMC7840757

[175] Gomez E, Atalah E, Salinas B. [Social cost effectiveness of 2 systems of treatment of malnourished children, in Chile]. Costo-efecto social de dos sistemas de tratamiento del nino desnutrido, en Chile. 1983;33:770–84. 6433832

[176] UNICEF. Evaluation of community management of acute malnutrition (CMAM): Chad country case study. New York: UNICEF; 2012.

[177] Tulloch C. Taking intervention costs seriously: a new, old toolbox for inference about costs. J Dev Effect. 2019;11:273–87. doi: 10.1080/19439342.2019.1684342

[178] Global Panel. The cost of malnutrition: why policy action is urgent. Technical Brief No.3. London: Global Panel on Agriculture and Food Systems for Nutrition; 2016. https://assets.publishing.service.gov.uk/media/59e09cc6e5274a11ac1c4971/CostOfMalnutrition.pdf

