# Supplementary material for: Costs and cost-effectiveness of treatment setting for children with wasting, oedema and growth failure/faltering: A systematic review
Source: PLOS Glob Public Health. 2023 Nov 8;3(11):e0002551. doi: 10.1371/journal.pgph.0002551 (PMC10631642; doi:10.1371/journal.pgph.0002551)
Supplement: S2 File — (PDF) [file pgph.0002551.s002.pdf]

S2 File. Key definitions

Population

For moderate wasting and severe wasting and/or bilateral pitting oedema, we accepted whatever definition or criteria a study used. Moderate wasting and severe wasting and/or bilateral pitting oedema cases are generally defined based on mid-upper arm circumference (MUAC) or weight for height z-score as per WHO's child growth standards and the presence or absence of bilateral oedema. Other studies also include other criteria such as a diagnosis of kwashiorkor, weight for age or height for age z-scores, apathy and skin and hair changes as part of their definitions.

For growth failure/faltering, in addition to the authors' definition, we also specifically defined this population using the following measures:

Single measures

Weight-for-length z scores (WLZ) <-2 (WHO 2006) (only available for infants >45cm)

Weight-for-age z scores (WAZ) <-2 (WHO 2006)

Low MUAC (undefined; generally ranging from <125mm to <110mm)

Length-for-age z scores (LAZ) <-2 (WHO 2006)

Small size at birth

Preterm (<37 weeks gestation)

Small for gestational age (SGA) at birth (using Intergrowth standards)

Low birth weight (LBW) (< 2500g)

Sequential measures

Losing weight

Weight loss in the early days of life is more than 10% of birth weight

Weight does not return to birth weight by 2 or 3 weeks of age

Weight falls across 2 or more weight centile spaces or falls across 1 whole z-score

Weight falls across 1 or more weight centile spaces if birth weight was below the 9th centile

Reported “sudden weight loss” by caregiver

Loss of weight or failure to gain weight over 2 monthly visits

Slow rate of growth (undefined)

Settings

For this review, the following definitions were used to distinguish between community, outpatient and inpatient settings:

> Community setting: any setting outside the health care setting. This includes community institutions, such as neighbourhoods, schools, churches, work sites, voluntary agencies, or other organizations [1].
>
> Outpatient setting: any health care setting where the services given to patients did not involve an overnight stay at a health facility [2].
>
> Inpatient setting: any health care setting where the services given to patients involved an overnight stay at a health facility [2].

Type of care

Treatment initiation, referral, transfer or discharge were defined as follows:

Initiation of treatment: was defined as the occasion when an infant or child is first introduced to treatment for growth faltering/failure, moderate wasting or severe wasting and/or bilateral pitting oedema [2].

Referral: was when an infant or child who has commenced treatment was directed to a different place, specifically a higher-level treatment institution, for further treatment.

Transfer: was defined as the movement of an infant or child from one place to another for treatment, where the movement was to a same- or lower-level treatment setting (i.e., from inpatient at a hospital to outpatient treatment at a primary health care centre).

Discharge: referred to when an infant or child was allowed to officially leave the nutrition treatment programme.

Costs

We defined studies as using financial and/or economic costs in their analyses as follows [3]:

Financial costs: if the analyses only included costs that represent actual expenditure on goods and services.

Economic costs: if the analyses included both financial costs and estimated market values for goods and services for which there is no actual purchase price, etc, for example, volunteer time, donated goods, etc.

We also defined studies as using full or incremental costs in their analyses as follows:

Full costs: if the analyses took into consideration the full costs of delivering services, a project, etc. This will often include basic infrastructure costs.

Incremental costs: if the analyses only consider the cost of adding an additional service, project, component, etc.

The costs were also defined as either modelled or empirical costs as follows:

Modelled costs: if costing involved the estimation of costs based on available information or estimated inputs, etc and/ or extrapolation to other populations and/ or over longer than the observed time period

Empirical costs: if the costs were based on inventories of inputs used to implement the programme and then application of prices to those inputs.

Costing approaches

Costing approaches were defined as follows:

Top-down: Starts from the overall costs of the program or implementation and divide them across the number of patients, users, etc.

Ingredients method: Uses a list of inputs derived from best practice or clinical guidelines.

Ingredients – modified: Uses a list of inputs derived from best practice or clinical guidelines, but some inputs may be modified to better fit the location where the costing is conducted.

Bottom-up: This type of costing would be based on actual resource utilization.

Types of analyses

The types of analyses were defined as follows:

Cost/ cost-efficiency: cost per output achieved, for example cost per child treated/ recovered

Cost-effectiveness: Comparisons of cost and outcomes between interventions to estimate the incremental cost required to gain a unit of health outcome, e.g., cost per death averted, cost per DALY averted etc.

References

1. McLeroy, K.R., et al., *Community-based interventions.* American Journal of Public Health, 2003. **93**(4): p. 529-33.
2. Cambridge University Press. *Cambridge Dictionary*. 2022 [cited 2022 18 March]; Available from: https://dictionary.cambridge.org/.
3. Meyer-Rath, G., et al., *The per-patient costs of HIV services in South Africa: Systematic review and application in the South African HIV Investment Case.* PLoS One, 2019. **14**(2): p. e0210497.
