# Supplementary material for: Costs and cost-effectiveness of treatment setting for children with wasting, oedema and growth failure/faltering: A systematic review
Source: PLOS Glob Public Health. 2023 Nov 8;3(11):e0002551. doi: 10.1371/journal.pgph.0002551 (PMC10631642; doi:10.1371/journal.pgph.0002551)
Supplement: S3 File — (PDF) [file pgph.0002551.s003.pdf]

S3 File. Search strategies

Cochrane Central Register of Controlled Trials (CENTRAL) via Cochrane Library

#1 ([mh Child] or [mh Infant] or [mh ^"Infant, Newborn, Diseases"] or (Child or Children or Childhood or Pre-school or Pre-schools or Preschool or Preschools or Infant or Infants or Infantile or New-Born or New-Borns or Newborn or Newborns or Neonate or Neonates or Neonatal or Toddler or Toddlers or Baby or Babies or "Early Life"):ti,ab) and ([mh "Wasting Syndrome"] or [mh "Failure to Thrive"] or [mh ^"Growth Disorders"] or [mh ^Malnutrition] or [mh "Child Nutrition Disorders"] or [mh "Infant Nutrition Disorders"] or [mh "Severe Acute Malnutrition"] or [mh Starvation] or [mh ^Edema] or [mh "Hydrops Fetalis"] or [mh "Protein Deficiency"] or (Waste or Wasted or Wasting or Stunt or Stunted or Stunting or Under Nutrition or UnderNutrition or Malnutrition or Under Nourished or Under Nourishment or Malnourished or Malnourishment or "Low Weight-For-Height" or "Low WFH" or "Severe Weight Loss" or "Rapid Weight Loss" or "Under Fed" or "Under Feed" or "Under Feeding" or Underfeeding or Underfed or Underfeed or "Under Weight" or Underweight or "Low Weight-For-Age" or "Low WFA" or "Low Birth Weight" or "Low Birthweight" or "Small for Gestational Age" or "Small for Date" or "Small for Age" or "Failure to Thrive" or "Growth Failure" or "Growth Faltering" or "Growth Disorder" or "Growth Disorders" or "Low Weight For Length" or "Low Mid Upper Arm Circumference" or Kwashiorkor or Marasmus or Starved or Starvation or Starving or Oedema or Oedemas or Oedematous or Edema or Edemas or Edematous or Hydrops or Dropsy or Anasarca or "Protein Deficiency" or "Protein Deprivation"):ti,ab) and ([mh "Costs and Cost Analysis"] or [mh "Cost-Benefit Analysis"] or [mh "Cost Control"] or [mh "Health Resources"] or [mh "Resource Allocation"] or [mh "Health Services Accessibility"] or [mh "Health Care Costs"] or [mh "Health Expenditures"] or [mh "Economics, Medical"] or (Cost or Costs or Cost-Effective or CostEffective or Cost-Effectiveness or CostEffectiveness or Cost-Efficiency or CostEfficiency or Cost-Efficient or CostEfficient or Cost Benefit or CostBenefit or Cost Benefitial or CostBenefitial or Cost Utility or CostUtility or "Cost Analysis" or Affordability or "Economic Evaluation" or "Economic Evaluations" or "Econometric Analysis" or "Economic Benefit" or "Economic Benefits" or "Marginal Analysis" or "Resource Allocation" or "Resources Allocation" or "Allocation of Resource" or "Allocation of Resources" or "Allocative

Efficiency" or "Health Care Rationing" or "Healthcare Rationing" or Finance or Finances or Financial or Financed or Expense or Expenses or Budget or Budgets or Budgeting or Expenditure or Expenditures or "Health Care Access" or "Health Care Accessibility" or "Access to Health Care" or "Healthcare Access" or "Healthcare Accessibility" or "Access to Healthcare"):ti,ab) NOT http*:so in Trials 466

Cochrane Database of Systematic Reviews (CDSR) via Cochrane Library

#1 ([mh Child] or [mh Infant] or [mh ^"Infant, Newborn, Diseases"] or (Child or Children or Childhood or Pre-school or Pre-schools or Preschool or Preschools or Infant or Infants or Infantile or New-Born or New-Borns or Newborn or Newborns or Neonate or Neonates or Neonatal or Toddler or Toddlers or Baby or Babies or "Early Life"):ti,ab) and ([mh "Wasting Syndrome"] or [mh "Failure to Thrive"] or [mh ^"Growth Disorders"] or [mh ^Malnutrition] or [mh "Child Nutrition Disorders"] or [mh "Infant Nutrition Disorders"] or [mh "Severe Acute Malnutrition"] or [mh Starvation] or [mh ^Edema] or [mh "Hydrops Fetalis"] or [mh "Protein Deficiency"] or (Waste or Wasted or Wasting or Stunt or Stunted or Stunting or Under Nutrition or UnderNutrition or Malnutrition or Under Nourished or Under Nourishment or Malnourished or Malnourishment or "Low Weight-For-Height" or "Low WFH" or "Severe Weight Loss" or "Rapid Weight Loss" or "Under Fed" or "Under Feed" or "Under Feeding" or Underfeeding or Underfed or Underfeed or "Under Weight" or Underweight or "Low Weight-For-Age" or "Low WFA" or "Low Birth Weight" or "Low Birthweight" or "Small for Gestational Age" or "Small for Date" or "Small for Age" or "Failure to Thrive" or "Growth Failure" or "Growth Faltering" or "Growth Disorder" or "Growth Disorders" or "Low Weight For Length" or "Low Mid Upper Arm Circumference" or Kwashiorkor or Marasmus or Starved or Starvation or Starving or Oedema or Oedemas or Oedematous or Edema or Edemas or Edematous or Hydrops or Dropsy or Anasarca or "Protein Deficiency" or "Protein Deprivation"):ti,ab) and ([mh "Costs and Cost Analysis"] or [mh "Cost-Benefit Analysis"] or [mh "Cost Control"] or [mh "Health Resources"] or [mh "Resource Allocation"] or [mh "Health Services Accessibility"] or [mh "Health Care Costs"] or [mh "Health Expenditures"] or [mh "Economics, Medical"] or (Cost or Costs or Cost-Effective or CostEffective or Cost-Effectiveness or CostEffectiveness or Cost-Efficiency or CostEfficiency or Cost-Efficient or CostEfficient or Cost Benefit or CostBenefit or Cost Benefitial or CostBenefitial or Cost Utility or CostUtility or "Cost Analysis" or Affordability or "Economic Evaluation" or

"Economic Evaluations" or "Econometric Analysis" or "Economic Benefit" or "Economic Benefits" or "Marginal Analysis" or "Resource Allocation" or "Resources Allocation" or "Allocation of Resource" or "Allocation of Resources" or "Allocative Efficiency" or "Health Care Rationing" or "Healthcare Rationing" or Finance or Finances or Financial or Financed or Expense or Expenses or Budget or Budgets or Budgeting or Expenditure or Expenditures or "Health Care Access" or "Health Care Accessibility" or "Access to Health Care" or "Healthcare Access" or "Healthcare Accessibility" or "Access to Healthcare"):ti,ab) NOT http*:so in Cochrane Reviews, Cochrane Protocols 77

Cost-Effectiveness Analysis (CEA) Registry via Center for the Evaluation of Value and Risk in Health (CEVR)

Search for: Methods

Wasting 3

Wasted 3

Waste 8

Stunt 0

Stunted 0

Stunting 0

Malnutrition 12

Growth Failure 0

Growth Faltering 0

Growth Disorder 0

Growth Disorders 0

Kwashiorkor 0

Marasmus 0

Starvation 0

Starving 0

Starved 0

Oedema 12

Edema 49

Protein Deficiency 0

Deprivation 29

Search for: Ratios

Wasting 8

Wasted 5

Waste 9

Stunt 0

Stunted 0

Stunting 0

Malnutrition 12

Growth Failure 0

Growth Faltering 0

Growth Disorder 0

Growth Disorders 0

Kwashiorkor 0

Marasmus 0

Starvation 0

Starving 0

Starved 0

Oedema 27

Edema 100

Protein Deficiency 0

Deprivation 56

DARE, HTA, NHSEED via CRDWeb

(Child OR Children OR Childhood OR Pre-school OR Pre-schools OR Preschool OR Preschools OR Infant OR Infants OR Infantile OR New-Born OR New-Borns OR Newborn OR Newborns OR Neonate OR Neonates OR Neonatal OR Toddler OR Toddlers OR Baby OR Babies OR Early Life) AND (Waste OR Wasted OR Wasting OR Stunt OR Stunted OR Stunting OR Under Nutrition OR UnderNutrition OR Malnutrition OR Under Nourished OR Under Nourishment OR Malnourished OR Malnourishment OR Low Weight-For-Height OR Low WFH OR Severe Weight Loss OR Rapid Weight Loss OR Under Fed OR Under Feed OR Under Feeding OR Underfeeding OR Underfed OR Underfeed OR Under Weight OR Underweight OR Low Weight-For-Age OR Low WFA OR Low Birth Weight OR Low Birthweight OR Small for Gestational Age OR Small for Date OR Small for Age OR Failure to Thrive OR Growth Failure OR Growth Faltering OR Growth Disorder OR Growth Disorders

OR Low Weight For Length OR Low Mid Upper Arm Circumference OR Kwashiorkor OR Marasmus OR Starved OR Starvation OR Starving OR Oedema OR Oedemas OR Oedematous OR Edema OR Edemas OR Edematous OR Hydrops OR Dropsy OR Anasarca OR Protein Deficiency OR Protein Deprivation) AND (Cost or Costs or Cost-Effective or CostEffective or Cost-Effectiveness or CostEffectiveness or Cost-Efficiency or CostEfficiency or Cost-Efficient or CostEfficient or Cost Benefit or CostBenefit or Cost Benefitial or CostBenefitial or Cost Utility or CostUtility or Cost Analysis or Affordability or Economic Evaluation or Economic Evaluations or Econometric Analysis or Economic Benefit or Economic Benefits or Marginal Analysis or Resource Allocation or Resources Allocation or Allocation of Resource or Allocation of Resources or Allocative Efficiency or Health Care Rationing or Healthcare Rationing or Finance or Finances or Financial or Financed or Expense or Expenses or Budget or Budgets or Budgeting or Expenditure or Expenditures or Health Care Access or Health Care Accessibility or Access to Health Care or Healthcare Access or Healthcare Accessibility or Access to Healthcare) IN DARE, NHSEED, HTA
212

EconLit via ProQuest Dialog

(ti(Child OR Children OR Childhood OR Pre-school OR Pre-schools OR Preschool OR Preschools OR Infant OR Infants OR Infantile OR New-Born OR New-Borns OR Newborn OR Newborns OR Neonate OR Neonates OR Neonatal OR Toddler OR Toddlers OR Baby OR Babies OR "Early Life") OR ab(Child OR Children OR Childhood OR Pre-school OR Pre-schools OR Preschool OR Preschools OR Infant OR Infants OR Infantile OR New-Born OR New-Borns OR Newborn OR Newborns OR Neonate OR Neonates OR Neonatal OR Toddler OR Toddlers OR Baby OR Babies OR "Early Life")) AND (ti(Waste OR Wasted OR Wasting OR Stunt OR Stunted OR Stunting OR Under Nutrition OR UnderNutrition OR Malnutrition OR Under Nourished OR Under Nourishment OR Malnourished OR Malnourishment OR "Low Weight-For-Height" OR "Low WFH" OR "Severe Weight Loss" OR "Rapid Weight Loss" OR "Under Fed" OR "Under Feed" OR "Under Feeding" OR Underfeeding OR Underfed OR Underfeed OR "Under Weight" OR Underweight OR "Low Weight-For-Age" OR "Low WFA" OR "Low Birth Weight" OR "Low Birthweight" OR "Small for Gestational Age" OR "Small for Date" OR "Small for Age" OR "Failure to Thrive" OR "Growth Failure" OR "Growth Faltering" OR "Growth Disorder" OR "Growth Disorders" OR "Low Weight

For Length" OR "Low Mid Upper Arm Circumference" OR Kwashiorkor OR Marasmus OR Starved OR Starvation OR Starving OR Oedema OR Oedemas OR Oedematous OR Edema OR Edemas OR Edematous OR Hydrops OR Dropsy OR Anasarca OR "Protein Deficiency" OR "Protein Deprivation") OR ab(Waste OR Wasted OR Wasting OR Stunt OR Stunted OR Stunting OR Under Nutrition OR UnderNutrition OR Malnutrition OR Under Nourished OR Under Nourishment OR Malnourished OR Malnourishment OR "Low Weight-For-Height" OR "Low WFH" OR "Severe Weight Loss" OR "Rapid Weight Loss" OR "Under Fed" OR "Under Feed" OR "Under Feeding" OR Underfeeding OR Underfed OR Underfeed OR "Under Weight" OR Underweight OR "Low Weight-For-Age" OR "Low WFA" OR "Low Birth Weight" OR "Low Birthweight" OR "Small for Gestational Age" OR "Small for Date" OR "Small for Age" OR "Failure to Thrive" OR "Growth Failure" OR "Growth Faltering" OR "Growth Disorder" OR "Growth Disorders" OR "Low Weight For Length" OR "Low Mid Upper Arm Circumference" OR Kwashiorkor OR Marasmus OR Starved OR Starvation OR Starving OR Oedema OR Oedemas OR Oedematous OR Edema OR Edemas OR Edematous OR Hydrops OR Dropsy OR Anasarca OR "Protein Deficiency" OR "Protein Deprivation")) AND (ti(Cost OR Costs OR Cost-Effective OR CostEffective OR Cost-Effectiveness OR CostEffectiveness OR Cost-Efficiency OR CostEfficiency OR Cost-Efficient OR CostEfficient OR Cost Benefit OR CostBenefit OR Cost Benefitial OR CostBenefitial OR Cost Utility OR CostUtility OR "Cost Analysis" OR Affordability OR "Economic Evaluation" OR "Economic Evaluations" OR "Econometric Analysis" OR "Economic Benefit" OR "Economic Benefits" OR "Marginal Analysis" OR "Resource Allocation" OR "Resources Allocation" OR "Allocation of Resource" OR "Allocation of Resources" OR "Allocative Efficiency" OR "Health Care Rationing" OR "Healthcare Rationing" OR Finance OR Finances OR Financial OR Financed OR Expense OR Expenses OR Budget OR Budgets OR Budgeting OR Expenditure OR Expenditures OR "Health Care Access" OR "Health Care Accessibility" OR "Access to Health Care" OR "Healthcare Access" OR "Healthcare Accessibility" OR "Access to Healthcare") OR ab(Cost OR Costs OR Cost-Effective OR CostEffective OR Cost-Effectiveness OR CostEffectiveness OR Cost-Efficiency OR CostEfficiency OR Cost-Efficient OR CostEfficient OR Cost Benefit OR CostBenefit OR Cost Benefitial OR CostBenefitial OR Cost Utility OR CostUtility OR "Cost Analysis" OR Affordability OR "Economic Evaluation" OR "Economic Evaluations" OR "Econometric Analysis" OR "Economic Benefit" OR "Economic Benefits" OR "Marginal Analysis" OR "Resource

Allocation" OR "Resources Allocation" OR "Allocation of Resource" OR "Allocation of Resources" OR "Allocative Efficiency" OR "Health Care Rationing" OR "Healthcare Rationing" OR Finance OR Finances OR Financial OR Financed OR Expense OR Expenses OR Budget OR Budgets OR Budgeting OR Expenditure OR Expenditures OR "Health Care Access" OR "Health Care Accessibility" OR "Access to Health Care" OR "Healthcare Access" OR "Healthcare Accessibility" OR "Access to Healthcare")) 306

Embase via Ovid SP

Database: Embase <1974 to 2021 December 10>

1 (exp *Child/ or exp *Infant/ or *Infant Disease/ or *Childhood Disease/ or (Child or Children or Childhood or Pre-school or Pre-schools or Preschool or Preschools or Infant or Infants or Infantile or New-Born or New-Borns or Newborn or Newborns or Neonate or Neonates or Neonatal or Toddler or Toddlers or Baby or Babies or "Early Life").ti,ab.) and (*Wasting Syndrome/ or *Failure to Thrive/ or *Growth Disorder/ or *Malnutrition/ or *Nutritional Disorder/ or *Starvation/ or *Edema/ or *Fetus Hydrops/ or *Stunting/ or exp *Low Birth Weight/ or *Marasmus/ or *Anasarca/ or exp *Protein Deficiency/ or Prematurity/ or Intrauterine Growth Retardation/ or (Waste or Wasted or Wasting or Stunt or Stunted or Stunting or Under Nutrition or UnderNutrition or Malnutrition or Under Nourished or Under Nourishment or Malnourished or Malnourishment or "Low Weight-For-Height" or "Low WFH" or "Severe Weight Loss" or "Rapid Weight Loss" or "Under Fed" or "Under Feed" or "Under Feeding" or Underfeeding or Underfed or Underfeed or "Under Weight" or Underweight or "Low Weight-For-Age" or "Low WFA" or "Low Birth Weight" or "Low Birthweight" or "Small for Gestational Age" or "Small for Date" or "Small for Age" or "Failure to Thrive" or "Growth Failure" or "Growth Faltering" or "Growth Disorder" or "Growth Disorders" or "Low Weight For Length" or "Low Mid Upper Arm Circumference" or Kwashiorkor or Marasmus or Starved or Starvation or Starving or Oedema or Oedemas or Oedematous or Edema or Edemas or Edematous or Hydrops or Dropsy or Anasarca or "Protein Deficiency" or "Protein Deprivation" or Prematur* or Pre-Matur* or Preterm or Pre-Term or "Fetal Growth Disorder" or "Fetal Growth Restriction" or "Fetal Growth Retardation" or "Fetus Growth Disorder" or "Fetus Growth Retardation" or "Foetal Growth Disorder" or "Foetal Growth Restriction" or "Foetal Growth Retardation" or "Foetus Growth Disorder" or "Foetus Growth Retardation" or "Growth Retardation in

Utero" or "in Utero Growth Restriction" or "in Utero Growth Retardation" or "Intrauterine Growth Restriction" or "intrauterine Growth Restriction" or "Intra-Uterine Growth Restriction" or "Intrauterine Growth Retardation" or "intra-Uterine Growth Retardation" or IUGR or "Prenatal Growth Retardation" or "Retarded Intrauterine Growth").ti,ab.) and (*Health Economics/ or exp *Economic Evaluation/ or *Cost/ or exp *Health Care Cost/ or *Program Cost Effectiveness/ or *Resource Allocation/ or exp *Health Care Access/ or (Cost or Costs or Cost-Effective or CostEffective or Cost-Effectiveness or CostEffectiveness or Cost-Efficiency or CostEfficiency or Cost-Efficient or CostEfficient or Cost Benefit or CostBenefit or Cost Benefitial or CostBenefitial or Cost Utility or CostUtility or "Cost Analysis" or Affordability or "Economic Evaluation" or "Economic Evaluations" or "Econometric Analysis" or "Economic Benefit" or "Economic Benefits" or "Marginal Analysis" or "Resource Allocation" or "Resources Allocation" or "Allocation of Resource" or "Allocation of Resources" or "Allocative Efficiency" or "Health Care Rationing" or "Healthcare Rationing" or Finance or Finances or Financial or Financed or Expense or Expenses or Budget or Budgets or Budgeting or Expenditure or Expenditures or "Health Care Access" or "Health Care Accessibility" or "Access to Health Care" or "Healthcare Access" or "Healthcare Accessibility" or "Access to Healthcare").ti,ab.) (9286)

2 limit 1 to embase (5339)

Epistemonikos

(title:(title:((Child OR Children OR Childhood OR Pre-school OR Pre-schools OR Preschool OR Preschools OR Infant OR Infants OR Infantile OR New-Born OR New-Borns OR Newborn OR Newborns OR Neonate OR Neonates OR Neonatal OR Toddler OR Toddlers OR Baby OR Babies OR "Early Life") AND (Waste OR Wasted OR Wasting OR Stunt OR Stunted OR Stunting OR Under Nutrition OR UnderNutrition OR Malnutrition OR Under Nourished OR Under Nourishment OR Malnourished OR Malnourishment OR "Low Weight-For-Height" OR "Low WFH" OR "Severe Weight Loss" OR "Rapid Weight Loss" OR "Under Fed" OR "Under Feed" OR "Under Feeding" OR Underfeeding OR Underfed OR Underfeed OR "Under Weight" OR Underweight OR "Low Weight-For-Age" OR "Low WFA" OR "Low Birth Weight" OR "Low Birthweight" OR "Small for Gestational Age" OR "Small for Date" OR "Small for Age" OR "Failure to Thrive" OR "Growth Failure" OR "Growth Faltering" OR "Growth Disorder" OR "Growth Disorders" OR "Low Weight For Length" OR "Low Mid Upper

Arm Circumference" OR Kwashiorkor OR Marasmus OR Starved OR Starvation OR Starving OR Oedema OR Oedemas OR Oedematous OR Edema OR Edemas OR Edematous OR Hydrops OR Dropsy OR Anasarca OR "Protein Deficiency" OR "Protein Deprivation") AND (Cost OR Costs OR Cost-Effective OR CostEffective OR Cost-Effectiveness OR CostEffectiveness OR Cost-Efficiency OR CostEfficiency OR Cost-Efficient OR CostEfficient OR Cost Benefit OR CostBenefit OR Cost Benefitial OR CostBenefitial OR Cost Utility OR CostUtility OR "Cost Analysis" OR Affordability OR "Economic Evaluation" OR "Economic Evaluations" OR "Econometric Analysis" OR "Economic Benefit" OR "Economic Benefits" OR "Marginal Analysis" OR "Resource Allocation" OR "Resources Allocation" OR "Allocation of Resource" OR "Allocation of Resources" OR "Allocative Efficiency" OR "Health Care Rationing" OR "Healthcare Rationing" OR Finance OR Finances OR Financial OR Financed OR Expense OR Expenses OR Budget OR Budgets OR Budgeting OR Expenditure OR Expenditures OR "Health Care Access" OR "Health Care Accessibility" OR "Access to Health Care" OR "Healthcare Access" OR "Healthcare Accessibility" OR "Access to Healthcare"))) OR abstract:(title:((Child OR Children OR Childhood OR Pre-school OR Pre-schools OR Preschool OR Preschools OR Infant OR Infants OR Infantile OR New-Born OR New-Borns OR Newborn OR Newborns OR Neonate OR Neonates OR Neonatal OR Toddler OR Toddlers OR Baby OR Babies OR "Early Life") AND (Waste OR Wasted OR Wasting OR Stunt OR Stunted OR Stunting OR Under Nutrition OR UnderNutrition OR Malnutrition OR Under Nourished OR Under Nourishment OR Malnourished OR Malnourishment OR "Low Weight-For-Height" OR "Low WFH" OR "Severe Weight Loss" OR "Rapid Weight Loss" OR "Under Fed" OR "Under Feed" OR "Under Feeding" OR Underfeeding OR Underfed OR Underfeed OR "Under Weight" OR Underweight OR "Low Weight-For-Age" OR "Low WFA" OR "Low Birth Weight" OR "Low Birthweight" OR "Small for Gestational Age" OR "Small for Date" OR "Small for Age" OR "Failure to Thrive" OR "Growth Failure" OR "Growth Faltering" OR "Growth Disorder" OR "Growth Disorders" OR "Low Weight For Length" OR "Low Mid Upper Arm Circumference" OR Kwashiorkor OR Marasmus OR Starved OR Starvation OR Starving OR Oedema OR Oedemas OR Oedematous OR Edema OR Edemas OR Edematous OR Hydrops OR Dropsy OR Anasarca OR "Protein Deficiency" OR "Protein Deprivation") AND (Cost OR Costs OR Cost-Effective OR CostEffective OR Cost-Effectiveness OR CostEffectiveness OR Cost-Efficiency OR CostEfficiency OR Cost-Efficient OR CostEfficient OR Cost Benefit OR CostBenefit

OR Cost Benefitial OR CostBenefitial OR Cost Utility OR CostUtility OR "Cost Analysis" OR Affordability OR "Economic Evaluation" OR "Economic Evaluations" OR "Econometric Analysis" OR "Economic Benefit" OR "Economic Benefits" OR "Marginal Analysis" OR "Resource Allocation" OR "Resources Allocation" OR "Allocation of Resource" OR "Allocation of Resources" OR "Allocative Efficiency" OR "Health Care Rationing" OR "Healthcare Rationing" OR Finance OR Finances OR Financial OR Financed OR Expense OR Expenses OR Budget OR Budgets OR Budgeting OR Expenditure OR Expenditures OR "Health Care Access" OR "Health Care Accessibility" OR "Access to Health Care" OR "Healthcare Access" OR "Healthcare Accessibility" OR "Access to Healthcare"))))
28

Global Health Cost Effectiveness Analysis (GH CEA) Registry via Center for the Evaluation of Value and Risk in Health (CEVR)
Disease: Nutritional deficiencies 357

Google Scholar
allintitle:Child|Children|Childhood|Infant|Infants|Neonate|Neonates|Neonatal Waste|Wasted|Wasting|Stunt|Stunted|Stunting|Malnutrition|"Failure to Thrive"|"Growth Failure"|Starved|Starvation|Starving Cost|Costs|Economic|Economics|Econometric
182

INAHTA HTA Database
(Child OR Children OR Childhood OR Pre-school OR Pre-schools OR Preschool OR Preschools OR Infant OR Infants OR Infantile OR New-Born OR New-Borns OR Newborn OR Newborns OR Neonate OR Neonates OR Neonatal OR Toddler OR Toddlers OR Baby OR Babies OR "Early Life") AND (Waste OR Wasted OR Wasting OR Stunt OR Stunted OR Stunting OR Under Nutrition OR UnderNutrition OR Malnutrition OR Under Nourished OR Under Nourishment OR Malnourished OR Malnourishment OR "Low Weight-For-Height" OR "Low WFH" OR "Severe Weight Loss" OR "Rapid Weight Loss" OR "Under Fed" OR "Under Feed" OR "Under Feeding" OR Underfeeding OR Underfed OR Underfeed OR "Under Weight" OR Underweight OR "Low Weight-For-Age" OR "Low WFA" OR "Low Birth Weight" OR

"Low Birthweight" OR "Small for Gestational Age" OR "Small for Date" OR "Small for Age" OR "Failure to Thrive" OR "Growth Failure" OR "Growth Faltering" OR "Growth Disorder" OR "Growth Disorders" OR "Low Weight For Length" OR "Low Mid Upper Arm Circumference" OR Kwashiorkor OR Marasmus OR Starved OR Starvation OR Starving OR Oedema OR Oedemas OR Oedematous OR Edema OR Edemas OR Edematous OR Hydrops OR Dropsy OR Anasarca OR "Protein Deficiency" OR "Protein Deprivation") AND (Cost or Costs or Cost-Effective or CostEffective or Cost-Effectiveness or CostEffectiveness or Cost-Efficiency or CostEfficiency or Cost-Efficient or CostEfficient or Cost Benefit or CostBenefit or Cost Benefitial or CostBenefitial or Cost Utility or CostUtility or "Cost Analysis" or Affordability or "Economic Evaluation" or "Economic Evaluations" or "Econometric Analysis" or "Economic Benefit" or "Economic Benefits" or "Marginal Analysis" or "Resource Allocation" or "Resources Allocation" or "Allocation of Resource" or "Allocation of Resources" or "Allocative Efficiency" or "Health Care Rationing" or "Healthcare Rationing" or Finance or Finances or Financial or Financed or Expense or Expenses or Budget or Budgets or Budgeting or Expenditure or Expenditures or "Health Care Access" or "Health Care Accessibility" or "Access to Health Care" or "Healthcare Access" or "Healthcare Accessibility" or "Access to Healthcare")

282

MEDLINE via Ovid SP

Database: Ovid MEDLINE(R) ALL <1946 to December 10, 2021>

1 (exp Child/ or exp Infant/ or Infant, Newborn, Diseases/ or (Child or Children or Childhood or Pre-school or Pre-schools or Preschool or Preschools or Infant or Infants or Infantile or New-Born or New-Borns or Newborn or Newborns or Neonate or Neonates or Neonatal or Toddler or Toddlers or Baby or Babies or "Early Life").ti,ab.) and (Wasting Syndrome/ or Failure to Thrive/ or Growth Disorders/ or Malnutrition/ or Child Nutrition Disorders/ or Infant Nutrition Disorders/ or exp Severe Acute Malnutrition/ or Starvation/ or Edema/ or Hydrops Fetalis/ or exp Protein Deficiency/ or Fetal Growth Retardation/ or (Waste or Wasted or Wasting or Stunt or Stunted or Stunting or Under Nutrition or UnderNutrition or Malnutrition or Under Nourished or Under Nourishment or Malnourished or Malnourishment or "Low Weight-For-Height" or "Low WFH" or "Severe Weight Loss" or "Rapid Weight Loss" or "Under Fed" or "Under Feed" or "Under Feeding" or Underfeeding or Underfed or Underfeed or "Under

Weight" or Underweight or "Low Weight-For-Age" or "Low WFA" or "Low Birth Weight" or "Low Birthweight" or "Small for Gestational Age" or "Small for Date" or "Small for Age" or "Failure to Thrive" or "Growth Failure" or "Growth Faltering" or "Growth Disorder" or "Growth Disorders" or "Low Weight For Length" or "Low Mid Upper Arm Circumference" or Kwashiorkor or Marasmus or Starved or Starvation or Starving or Oedema or Oedemas or Oedematous or Edema or Edemas or Edematous or Hydrops or Dropsy or Anasarca or "Protein Deficiency" or "Protein Deprivation" or Prematur* or Pre-Matur* or Preterm or Pre-Term or "Fetal Growth Disorder" or "Fetal Growth Restriction" or "Fetal Growth Retardation" or "Fetus Growth Disorder" or "Fetus Growth Retardation" or "Foetal Growth Disorder" or "Foetal Growth Restriction" or "Foetal Growth Retardation" or "Foetus Growth Disorder" or "Foetus Growth Retardation" or "Growth Retardation in Utero" or "in Utero Growth Restriction" or "in Utero Growth Retardation" or "Intrauterine Growth Restriction" or "intrauterine Growth Restriction" or "Intra-Uterine Growth Restriction" or "Intrauterine Growth Retardation" or "intra-Uterine Growth Retardation" or IUGR or "Prenatal Growth Retardation" or "Retarded Intrauterine Growth").ti,ab.) and ("Costs and Cost Analysis"/ or Cost-Benefit Analysis/ or exp Cost Control/ or Health Resources/ or exp Resource Allocation/ or exp Health Services Accessibility/ or exp Health Care Costs/ or Health Expenditures/ or exp Economics, Medical/ or (Cost or Costs or Cost-Effective or CostEffective or Cost-Effectiveness or CostEffectiveness or Cost-Efficiency or CostEfficiency or Cost-Efficient or CostEfficient or Cost Benefit or CostBenefit or Cost Benefitial or CostBenefitial or Cost Utility or CostUtility or "Cost Analysis" or Affordability or "Economic Evaluation" or "Economic Evaluations" or "Econometric Analysis" or "Economic Benefit" or "Economic Benefits" or "Marginal Analysis" or "Resource Allocation" or "Resources Allocation" or "Allocation of Resource" or "Allocation of Resources" or "Allocative Efficiency" or "Health Care Rationing" or "Healthcare Rationing" or Finance or Finances or Financial or Financed or Expense or Expenses or Budget or Budgets or Budgeting or Expenditure or Expenditures or "Health Care Access" or "Health Care Accessibility" or "Access to Health Care" or "Healthcare Access" or "Healthcare Accessibility" or "Access to Healthcare").ti,ab.) (8263)

WHO via Google

allintitle:Child|Childhood|Children|Infant|Infants|Neonate|Neonates|Neonatal Waste|Wasting|Wasted|Stunt|Stunted|Stunting|Malnutrition|"Failure to Thrive"|"Growth Failure" site:who.int
227

UNICEF via Google
allintitle:Child|Childhood|Children|Infant|Infants|Neonate|Neonates|Neonatal Waste|Wasting|Wasted|Stunt|Stunted|Stunting|Malnutrition|"Failure to Thrive"|"Growth Failure" site:unicef.org
180

World Bank via Google
allintitle:Child|Childhood|Children|Infant|Infants|Neonate|Neonates|Neonatal Waste|Wasting|Wasted|Stunt|Stunted|Stunting|Malnutrition|"Failure to Thrive"|"Growth Failure" site:worldbank.org
64

Action on Hunger via Google
allintitle:Child|Childhood|Children|Infant|Infants|Neonate|Neonates|Neonatal Waste|Wasting|Wasted|Stunt|Stunted|Stunting|Malnutrition|"Failure to Thrive"|"Growth Failure" site:actionagainsthunger.org
30

Save the Children via Google
allintitle:Child|Childhood|Children|Infant|Infants|Neonate|Neonates|Neonatal Waste|Wasting|Wasted|Stunt|Stunted|Stunting|Malnutrition|"Failure to Thrive"|"Growth Failure" site:savethechildren.org
12

MSF via Google
allintitle:Child|Childhood|Children|Infant|Infants|Neonate|Neonates|Neonatal Waste|Wasting|Wasted|Stunt|Stunted|Stunting|Malnutrition|"Failure to Thrive"|"Growth Failure" site:msf.org
57
