# Supplementary material for: Costs and cost-effectiveness of treatment setting for children with wasting, oedema and growth failure/faltering: A systematic review
Source: PLOS Glob Public Health. 2023 Nov 8;3(11):e0002551. doi: 10.1371/journal.pgph.0002551 (PMC10631642; doi:10.1371/journal.pgph.0002551)
Supplement: S4 File — (PDF) [file pgph.0002551.s004.pdf]

S4 File. Data extraction categories and variables

Category	Variables
General information	Author<br>Publication year<br>Country<br>Region
Key study methodology characteristics	Study design e.g., trial-based, microsimulation model<br>Type of economic evaluation e.g., cost, cost-effectiveness analysis (CEA), cost-utility analysis (CUA), budget impact analysis (BIA)<br>Comparators<br>Cost perspective<br>Analytical approach (i.e., cost data collection method)<br>Costing period<br>Cost year (reference year for costs)<br>Cost currency<br>Exchange rate<br>Sample size/number of patients<br>Form of child wasting (moderate or severe and oedema)
Targeted population	Condition (i.e., moderate wasting, severe wasting and/or bilateral pitting oedema, or growth faltering/failure)<br>Age range<br>Gender<br>Ethnicity
Intervention	Type of care i.e., treatment initiation; referral; transfer; discharge

	Care setting i.e., community, outpatient, inpatient etc.
Outcomes	Type of resources (e.g., staff, capital, equipment, overheads, drugs, transport, hospitalisation, other)<br><br>Cost categories (e.g., direct medical costs, direct non-medical costs, indirect costs, total costs, and cost drivers).<br><br>Results of cost analysis (cost per?, cost, type of range, low range, high range)
