# Supplementary material for: Costs and cost-effectiveness of treatment setting for children with wasting, oedema and growth failure/faltering: A systematic review
Source: PLOS Glob Public Health. 2023 Nov 8;3(11):e0002551. doi: 10.1371/journal.pgph.0002551 (PMC10631642; doi:10.1371/journal.pgph.0002551)
Supplement: S5 File — (PDF) [file pgph.0002551.s005.pdf]

S5 File. Characteristics of excluded studies

First author	Title	Reason for exclusion	Comment
Akseer et al 2021 [49]	Economic costs of childhood stunting to the private sector in low-and middle-income countries	Intervention	Not about treatment-looking at impact of stunting on income loss of private sector workforce
Ale et al 2016 [50]	Mothers screening for malnutrition by mid-upper arm circumference is non-inferior to community health workers: results from a large-scale pragmatic trial in rural Niger	Intervention	Evaluating a screening strategy
Assad et al 2016 [51]	Decreased cost and improved feeding tolerance in VLBW infants fed an exclusive human milk diet	Intervention	Not about treatment setting
Cobb and Bland 2013 [53]	Nutritional supplementation: the additional costs of managing children infected with HIV in resource-constrained settings	Intervention	Doesn't seem to include treatment setting costs; mainly comparing the supplementary foods themselves

Bergmann et al 2017 [52]	Outcomes and cost-effectiveness of integrating HIV and nutrition service delivery: pilots in Malawi and Mozambique	Intervention	Integrated HIV and nutrition services; also looking at both prevention and treatment
De Lauture et al 1982 [54]	A model for combatting malnutrition in children: nutritional rehabilitation centers	Intervention	Unclear if it is inpatient or outpatient treatment.
Thompson et al 2017 [55]	Increased length of stay and costs associated with weekend admissions for failure to thrive	Intervention	Weekend hospital admissions
Guiti et al 1979 [87]	Cost of hospital treatment for children with severe protein calorie malnutrition	Intervention	Initiation of treatment in inpatient settings
Griswold et al 2021 [56]	Effectiveness and cost-effectiveness of 4 supplementary foods for treating moderate acute malnutrition: results from a cluster-randomized intervention trial in Sierra Leone	Intervention	Doesn't seem to include treatment setting costs; mainly comparing the supplementary foods themselves
Hossain et al 2009 [57]	Experience in managing severe malnutrition in a government tertiary treatment facility in Bangladesh	Intervention	Initiation of treatment in inpatient settings
Laillou et al 2020 [60]	Wasted children and wasted time: A challenge to meeting the	Intervention	Treatment setting not specified

	nutrition sustainable development goals with a high economic impact to Ethiopia		
Lima et al 2000 [61]	Feasibility, acceptability and cost of kangaroo mother care in Recife, Brazil	Intervention	Not a nutrition intervention
Lukaczer 1971 [63]	Lessons for the federal effort against hunger and malnutrition--from a case study	Intervention	Prevention not treatment
Malan et al 1992 [63]	The cost of neonatal care	Intervention	Treatment in inpatient settings
Marino et al 2013 [64]	Cost comparison between powdered versus energy dense infant formula for undernourished children in a hospital setting	Intervention	Initiation of treatment in inpatient settings
Nordermoen and Bratlid 2010 [65]	[Costs for treatment of very-low-birth-weight infants]	Intervention	Treatment in inpatient settings; not on costs related to nutrition interventions
Parker et al 2012 [66]	Effect of breast milk on hospital costs and length of stay among very low-birth-weight infants in the NICU	Intervention	Treatment in inpatient settings
Peabody et al 2017 [67]	Comparative effectiveness of two disparate policies on child health:	Intervention	Treatment setting not specified

	experimental evidence from the Philippines		
Rice et al 2010 [68]	Economic evaluation of enhanced staff contact for the promotion of breastfeeding for low birth weight infants	Intervention	Treatment in inpatient settings
Russell et al 2007 [69]	Cost of hospitalization for preterm and low birth weight infants in the United States	Intervention	Treatment in inpatient settings
Seigel et al 2014 [58]	Economic impact of human milk on medical charges of extremely low birth weight infants.	Intervention	Economic impact, and not seeking answers on the setting
Sharma et al 2017 [86]	To compare growth outcomes and cost-effectiveness of "Kangaroo ward care" with "intermediate intensive care" in stable extremely low birth weight infants: randomized control trial	Intervention	Treatment in inpatient settings
Scholz et al 2019 [70]	An exclusive human milk diet for very low birth weight newborns-A cost-effectiveness and EVPI study for Germany	Intervention	Treatment in inpatient settings
Stevenson et al 1991 [71]	Predicting costs and outcomes of neonatal intensive care for very low birthweight infants	Intervention	Treatment in inpatient settings

Tahir et al 2020 [72]	Economic evaluation alongside the Speed of Increasing milk Feeds Trial (SIFT)	Intervention	Treatment in inpatient settings
Taylor et al 2018 [73]	Prioritising allocation of donor human breast milk amongst very low birthweight infants in middle-income countries	Intervention	Strategies for allocating donor milk
Thanh et al 2015 [74]	Health service use and costs associated with low birth weight--a population level analysis	Intervention	Not specific to a treatment setting; No costs related to nutrition interventions
Tommiska et al 2003 [75]	Economic costs of care in extremely low birthweight infants during the first 2 years of life	Intervention	Treatment in inpatient settings
Tongo et al 2009 [76]	The economic burden of preterm/very low birth weight care in Nigeria	Intervention	Treatment in inpatient settings
Trang et al 2018 [77]	Cost-effectiveness of supplemental donor milk versus formula for very low birth weight infants	Intervention	Supplemental donor human milk (DHM) versus preterm formula (PTF)
Tudehope et al 1989 [78]	Cost-analysis of neonatal intensive and special care	Intervention	Treatment in inpatient settings

Vahidi et al 2014 [79]	Cost and effectiveness analysis of Kangaroo mother care and conventional care method in low-birth-weight neonates in Tabriz 2010-2011	Intervention	No nutrition costs; Treatment in inpatient settings
Daga et al 2010 [80]	Syndromic management of common illnesses in hospitalized children and neonates: A cost identification study	Intervention	No nutrition costs; Treatment in inpatient settings
Walker et al 1985 [81]	Economic analysis of regionalized neonatal care for very low-birth-weight infants in the state of Rhode Island	Intervention	Treatment in inpatient settings
Westrupp et al 2014 [82]	Community-based healthcare costs for children born low birthweight, preterm and/or small for gestational age: data from the longitudinal study of Australian children	Intervention	Looking at costs beyond the period of treatment in hospital settings
Wong and Radin 2019 [83]	Benefit-cost analysis of a package of early childhood interventions to improve nutrition in Haiti	Intervention	Treatment setting not specified
Wynn et al 2017 [84]	Mentor mothers program improved child health outcomes	Intervention	Prevention not treatment

	at a relatively low cost in South Africa		
Yang et al 2013 [59]	Ready-to-use food-allocation policy to reduce the effects of childhood undernutrition in developing countries.	Intervention	Strategies for allocating ready to use therapeutic foods
Zoungrana et al 2019 [85]	Effectiveness and cost of management of severe acute malnutrition with complications in kaya, Burkina Faso	Intervention	Treatment in inpatient settings
Ackatia-Armah et al 2015 [88]	Malian children with moderate acute malnutrition who are treated with lipid-based dietary supplements have greater weight gains and recovery rates than those treated with locally produced cereal-legume products: A community-based, cluster-randomized trial	Outcome	Effectiveness outcomes
Aguayo et al 2014 [89]	Providing care for children with severe acute malnutrition in India: new evidence from Jharkhand	Outcome	Effectiveness outcomes
Greco et al 2006 [90]	Effect of a low-cost food on the recovery and death rate of malnourished children	Outcome	Effectiveness outcomes

Binns et al 2016 [91]	Evidence-based interventions for improvement of maternal and child nutrition: What can be done and at what cost?	Outcome	Safety and practicability outcomes
Bredow et al 1994 [92]	Community based, effective, low cost approach to the treatment of severe malnutrition in rural Jamaica	Outcome	Effectiveness outcomes
Coppieters and Parent 2000 [93]	Assessment of hospital morbidity, mortality, and cost-effectiveness of a nutritional program for children under 5 years of age in Pala, Chad	Outcome	Effectiveness outcomes
Fink et al 2017 [94]	Home- and community-based growth monitoring to reduce early life growth faltering: An open-label, cluster-randomized controlled trial	Outcome	Effectiveness outcomes
Griswold et al 2019 [95]	Comparative cost-effectiveness of four supplementary foods in treating moderate acute malnutrition in children 6–59 months in Sierra Leone, report to USAID from the Food Aid Quality Review: Section3: Environmental Enteric Dysfunction Sub-Study	Outcome	No cost-related outcomes reported; focusing on effectiveness outcomes among children with environmental enteric dysfunction

Korachais et al 2020 [96]	Impact of the extension of a performance-based financing scheme to nutrition services in Burundi on malnutrition prevention and management among children below five: A cluster-randomized control trial	Outcome	Effectiveness outcomes
Kozuki et al 2020 [97]	Severe acute malnutrition treatment delivered by low-literate community health workers in South Sudan: A prospective cohort study	Outcome	Effectiveness outcomes
Lagrone et al 2010 [98]	Locally produced ready-to-use supplementary food is an effective treatment of moderate acute malnutrition in an operational setting	Outcome	Full text article doesn't have any cost data
Lack 2012 [99]	A tipping point for child survival, health, and nutrition	Outcome	No cost data
Lansot 1963 [100]	Economic and social factors in infant malnutrition. Study of a sample of 200 families of children admitted to the" Dr. Pedro Visca" hospital, August-September 1961	Outcome	No cost data

Laugesen 1974 [101]	A weight chart and weighing scale for nutrition surveys and grading of malnutrition in clinics	Outcome	No cost data
Hummer et al 2014 [102]	Low birth weight and health expenditures from birth to late adolescence	Outcome	No costs related to nutrition interventions
Leroy and Olney 2013 [103]	The evaluation of tubaramure in Burundi: an integrated food aid program	Outcome	No cost data
Leveno et al 1985 [104]	Prenatal care and the low-birth-weight infant	Outcome	No costs related to nutrition interventions
Lewit et al 1995 [105]	The direct cost of low birth weight	Outcome	No costs related to nutrition interventions
Lim et al 2009 [106]	CIHI survey: Hospital costs for preterm and small-for-gestational age babies in Canada	Outcome	No costs related to nutrition interventions
Magnin et al 2018 [107]	A realistic evaluation approach highlighted the success factors and difficulties of an innovative and comprehensive malnutrition programme in Madagascar	Outcome	No cost data
Marini 2004 [108]	Three essays on economic determinants of child malnutrition	Outcome	No setting costs

Marzouk et al 2017 [109]	Prenatal and post-natal cost of small for gestational age infants: a national study	Outcome	No costs related to nutrition interventions
Meregaglia et al 2020 [110]	Low socio-economic conditions and prematurity-related morbidities explain healthcare use and costs for 2-year-old very preterm children	Outcome	No costs related to nutrition interventions
Moench-Pfanner and Bloem 2013 [111]	ASEAN: insights and considerations toward nutrition programs	Outcome	No cost data
Moench-Pfanner et al 2016 [112]	The economic burden of malnutrition in pregnant women and children under 5 years of age in Cambodia	Outcome	No setting costs
Newman 1986 [113]	The very low birthweight infant - what cost?	Outcome	No costs related to nutrition interventions
Norgaard et al 2021 [114]	Cost of illness in young children: a prospective birth cohort study	Outcome	Cost of illness
Owino et al 2014 [115]	Development and acceptability of a novel milk-free soybean-maize-sorghum ready-to-use therapeutic food (SMS-RUTF)	Outcome	No cost data

	based on industrial extrusion cooking process		
Beam et al 2020 [116]	Estimates of healthcare spending for preterm and low-birthweight infants in a commercially insured population: 2008-2016	Outcome	No costs related to nutrition interventions
Raketa et al 2020 [117]	Evaluation of the effectiveness of cost-free nutrition programme on children in Reo health district, Burkina Faso	Outcome	No cost data
Rashid et al 2019 [118]	Efficacy of F-75 & F-100 recipes in the treatment of severe acute malnutrition: a randomized controlled trial	Outcome	No cost data
Rogowski 1998 [119]	Cost-effectiveness of care for very low birth weight infants	Outcome	No costs related to nutrition interventions
Sandhu et al 1986 [120]	Cost of neonatal intensive care for very-low-birthweight infants	Outcome	No costs related to nutrition interventions
Segre et al 2017 [121]	Local versus offshore production of ready-to-use therapeutic foods and small quantity lipid-based nutrient supplements	Outcome	Production costs; also a review
Shen et al 2017 [122]	Costing methods for a cluster-randomized cost-effectiveness	Outcome	No setting costs

	trial comparing the performance of four supplementary foods in treating Sierra Leonean children with moderate acute malnutrition (MAM)		
Steenson et al 1996 [123]	Cost of care for a geographically determined population of low birthweight infants to age 8-9 years. I. Children without disability	Outcome	No costs related to nutrition interventions
Stevenson et al 1996 [124]	Cost of care for a geographically determined population of low birthweight infants to age 8-9 years. II. Children with disability	Outcome	No costs related to nutrition interventions
Wanzira 2019 [125]	Supportive supervision as an approach to improve the quality of care for children with acute malnutrition in Arua district, Uganda: Baseline systematic assessment, cluster Randomised controlled trial and cost-effectiveness analysis	Outcome	No cost data in the retrieved study paper (Lazzerini et al 2019)
Yu and Bajuk 1981 [126]	Medical expenses of neonatal intensive care for very low birthweight infants	Outcome	No costs related to nutrition interventions

Ahmed et al 2021 [31]	Cost-effectiveness of a market-based home fortification of food with micronutrient powder programme in Bangladesh	Population	Children with Fe deficiency anaemia
Altare et al 2017 [32]	Unconditional cash transfers do not prevent children's undernutrition in the moderate acute malnutrition out (MAM'Out) cluster-randomized controlled trial in rural Burkina Faso	Population	Prevention intervention so included all children under the age of 1year
Aye et al 2020 [33]	Stunting: an overlooked problem in Myanmar - an economic evaluation	Population	Stunting in children under the age of 5years
Colombatti et al 2008 [34]	A short-term intervention for the treatment of severe malnutrition in a post-conflict country: Results of a survey in Guinea Bissau	Population	Children up to 17 years
Delport et al 2020 [35]	Ending malnutrition in all its forms requires scaling up proven nutrition interventions and much more: a 129-country analysis	Population	Not specific to wasting or growth faltering; and not specific to the age groups of interest
Galasso and Wagstaff 2019 [36]	The aggregate income losses from childhood stunting and the returns to a nutrition intervention aimed at reducing stunting	Population	Stunting in childhood

Goudet et al 2018 [37]	Cost effectiveness of a community-based prevention and treatment of acute malnutrition programme in Mumbai slums, India	Population	Includes children without wasting or growth failure/faltering as it is also looking at prevention; no separation of costs
Heckert et al 2020 [38]	The cost of improving nutritional outcomes through food-assisted maternal and child health and nutrition programmes in Burundi and Guatemala	Population	Pregnant women and children aged up to 2 years included in the sample- targeted at preventing malnutrition.
Korte 1974 [39]	Operational aspects of different approaches to nutritional rehabilitation	Population	18-84 months and the results for up to 5years not reported separately
Lakdawalla et al 2014 [40]	Impact of oral nutrition supplements on hospital outcomes in pediatric patients	Population	Paediatric population treated with oral nutrition supplements; not necessarily MAM/SAM/growth faltering

Langendorf et al 2014 [41]	Preventing acute malnutrition among young children in crises: a prospective intervention study in Niger	Population	Aimed at prevention among children regardless of nutrition status
Mason et al 1974 [42]	Treatment of severe malnutrition in relief	Population	
Melville et al 1995 [43]	Growth monitoring: The role of community health volunteers	Population	Healthy children; prevention not treatment
Neufeld et al 2019 [44]	A fortified food can be replaced by micronutrient supplements for distribution in a Mexican social protection program based on results of a cluster-randomized trial and costing analysis	Population	Pregnant and lactating women and children
Puett et al 2013 [45]	Protecting child health and nutrition status with ready-to-use food in addition to food assistance in urban Chad: A cost-effectiveness analysis	Population	Children not acutely malnourished; prevention not treatment
Shen et al 2020 [46]	Impact of stakeholder perspectives on cost-effectiveness estimates of four specialized nutritious foods for preventing stunting and wasting	Population	Includes children without wasting or growth failure/faltering as it is looking at prevention

	in children 6-23 months in Burkina Faso		
Tandon et al 2018 [47]	A randomised controlled trial of ready to use therapeutic food (RUTF) for moderate/severe acute malnourished Indian children with cancer	Population	Children 5-15years
Whittaker et al 1985 [48]	The cost effectiveness of the Philani Nutrition Day Centre in Crossroads squatter camp, Cape Town	Population	Aged 0-7years
Huybregts et al 2017 [142]	The impact of integrated prevention and treatment on child malnutrition and health: the PROMIS project, a randomized control trial in Burkina Faso and Mali	Publication type	Protocol
Levitt et al 1993 [143]	Low-birth-weight symposium: summary of proceedings	Publication type	Symposium summary
Mason 1999 [144]	Investing in Child Nutrition in Asia	Publication type	Book/summaries
Melh et al 2004 [145]	The cost-benefit threshold for low-birth-weight infants [1] (multiple letters)	Publication type	Letter to the Editor

Ashworth et al 2006 [127]	Efficacy and effectiveness of community-based treatment of severe malnutrition	Study design	Review
Ayokunle and Odusoga 2014 [128]	Community-based management of micronutrient deficiency in malnourished children in Ghana	Study design	Review
Bachmann 2010 [129]	Cost-effectiveness of community-based treatment of severe acute malnutrition in children	Study design	Review
Bhutta et al 2013 [130]	Evidence-based interventions for improvement of maternal and child nutrition: What can be done and at what cost?	Study design	Review
Collins 2007 [131]	Treating severe acute malnutrition seriously	Study design	Review
Collins et al 2006 [132]	Key issues in the success of community-based management of severe malnutrition	Study design	Review
Horton 1992 [133]	Unit costs, cost-effectiveness, and financing of nutrition interventions	Study design	Review
Manary et al 2020 [134]	Role of Optimized Plant Protein Combinations as a Low-Cost Alternative to Dairy Ingredients in Foods for Prevention and Treatment of Moderate Acute	Study design	Review

	Malnutrition and Severe Acute Malnutrition		
McCamish 1993 [135]	Malnutrition and nutrition support interventions: cost, benefits, and outcomes	Study design	Review
McLachlan 2006 [136]	Tackling the child malnutrition problem: From what and why to how much and how	Study design	Review
Mizumoto et al 2015 [137]	Health economics of nutrition intervention in Asia: cost of malnutrition	Study design	Review
Popkin 1978 [138]	Some economic aspects of planning health interventions among malnourished populations	Study design	Review
Schofield 1995 [139]	Treatment of malnutrition	Study design	Review
Suri et al 2016 [140]	The role of dairy in the comparative effectiveness and cost of fortified blended foods versus ready-to-use foods in treatment of children with moderate acute malnutrition	Study design	Review
Tica 1978 [141]	Food mixtures of high nutritional value and low cost in the fight	Study design	Review

	against protein-calorie<br><br>malnutrition		
