# Supplementary material for: Costs and cost-effectiveness of treatment setting for children with wasting, oedema and growth failure/faltering: A systematic review
Source: PLOS Glob Public Health. 2023 Nov 8;3(11):e0002551. doi: 10.1371/journal.pgph.0002551 (PMC10631642; doi:10.1371/journal.pgph.0002551)
Supplement: S6 File — (PDF) [file pgph.0002551.s006.pdf]

S6 File. Study definitions for moderate wasting, severe wasting and/or bilateral pitting oedema, or growth failure/faltering and inclusion/exclusion criteria

**First author (Year)**	**Inclusion criteria**
Akram (2016) [146]	Mid upper arm circumference (MUAC) less than 115mm; and a z score of <-3 based on WHO Child Growth Standards
Ali (2017) [147]	Weight-for-height z-score <-3SD, and/or MUAC less than 115mm, and/or have oedema
Ashraf (2019) [148]	Weight-for-height/length less than minus 3 Z-scores (<-3 WHZ); weight-for-age less than minus 3 Z-scores (<-3 WAZ); height/length for age less than minus 3 Z-scores (<-3 HAZ); bilateral pitting oedema; mid upper arm circumference (MUAC) < 115 mm in children 6–59 months old.
Ashworth (1997) [149]	Weight-for-height <60% of the reference median (not critically ill)
Bachmann (2009) [150]	MUAC of 11 cm or less, or bilateral pitting oedema

First author (Year)	Inclusion criteria
Bai (1972) [151]	Protein-calorie malnutrition with retarded growth, apathy, oedema, skin and hair changes
Bailey (2020) [152]	Children with MUAC<12.5cm and/or edema & MUAC 11.5 to <12.5 cm and clinically uncomplicated
Chapko (1994) [153]	About to be discharged from the paediatrics service at the national hospital; weight-for-height below —2 SD or a diagnosis of kwashiorkor; residing within Niamey
Fotso (2019) [154]	Children with SAM based on weight-for-height Z-score <-3SD
Frankel (2015) [155]	Children with severe acute malnutrition
Garg (2018) [156]	Weight-for-height <−3 SD of the WHO standard, and/or oedema of both feet, or both were included. Complicated SAM excluded.
Gomez (1983) [175]	NCHS/WHO: mild= 95 and 86%, moderate= 85-76%, and severe= <76% of the weight/height ratio; normal 96 to 110% and overweight >110%.

First author (Year)	Inclusion criteria
IRC (2016) [157]	Children under the age of five with severe acute malnutrition
Isanaka (2017) [160]	SAM; MUAC <11.5, WHZ <-3 and bilateral oedema
Isanaka (2019) [158]	WHO standards <=-3SD; Weight for Length Z score <-2; Mid upper arm circumference <=11,5cm/<12,5 cm
Karniski (1986) [159]	<5th percentile for weight
Masiiwa (2013) [161]	Severely malnutritioned children admitted for inpatient care
N'Diaye (2020) [162]	Weight-for-height z score <−3 and/or a mid-upper arm circumference (MUAC) < 115 mm, no oedema, passed an appetite test and had no medical complications
Nkonki (2017) [163]	Moderately wasted (-3 TO -2Z)
	Severely wasted children (<-3Z)

**First author (Year)**	**Inclusion criteria**
Puette (2013) [164]	Cases of SAM with no medical complications
Purwestri (2012) [165]	Weight-for-height z-score (WHZ) ≥ –3 to < –1.5, no birth defect or disease limiting ad libitum food intake.
Reed (2012a) [168]	MUAC 11.5 - 12.5 cm and registered for supplementary feeding
	MUAC<11.5 cm with bilateral oedema and medical complications
	MUAC<11.5 cm with good appetite and no medical complications
Reed (2012b) [167]	MUAC < 11.5 cm
	MUAC ≥ 11.5 cm and < 12.5 cm
Reed (2012c) [166]	MUAC <11.5, SAM without complications
	MUAC >11.5 -<12.5
Rogers (2017) [169]	Caregivers of children identified as having MAM at health clinics or during community screenings.
Rogers (2018) [171]	SAM; MUAC <11.5, WHZ <-3 and bilateral oedema

**First author (Year)**	**Inclusion criteria**
Rogers (2019) [170]	Uncomplicated SAM cases
Tekeste (2012) [172]	SAM with WHZ<60% median
UNICEF (2012) [176]	Bilateral lower limb oedema grade + or ++ or P/T index <180; -3 z-scores, or PB <115mm; P/T > 1 000; -3 & -2 z-scores or a PB >115 and < 125 mm
Wilford (2011) [173]	SAM; WHZ<-3, MUAC 11.5, Oedema
Wilunda (2021) [174]	SAM; WHZ<-3, MUAC 11.5, Oedema
