# Supplementary material for: Costs and cost-effectiveness of treatment setting for children with wasting, oedema and growth failure/faltering: A systematic review
Source: PLOS Glob Public Health. 2023 Nov 8;3(11):e0002551. doi: 10.1371/journal.pgph.0002551 (PMC10631642; doi:10.1371/journal.pgph.0002551)
Supplement: S1 Table — (DOCX) [file pgph.0002551.s007.docx]

**S1 Table. Cost analysis results for management of growth failure/faltering in infants <12 months of age**

| **Author, year** | **Country, WHO region** | **Target population** | **Treatment arms** | **Setting, level of care/treatment setting** | **Cost perspective** | **Cost per** | | |
| --- | --- | --- | --- | --- | --- | --- | --- | --- |
|  |  |  |  |  |  | **Child treated** | **Child recovered** | **Other** |
| **Transfer from inpatient to community treatment** | | | | | | | | |
| Karniski (1986) [159] | USA; Americas | Growth faltering; <12 months | Transfer from an inpatient to a community setting | Urban; medical placement home | Provider | $6,776 |  |  |
| **Treatment in an inpatient setting** | | | | | | | | |
| Karniski (1986) [159] | USA; Americas | Growth faltering; <12 months | Inpatient treatment | Urban; hospital | Provider | $5,773 |  |  |
