# Supplementary material for: Costs and cost-effectiveness of treatment setting for children with wasting, oedema and growth failure/faltering: A systematic review
Source: PLOS Glob Public Health. 2023 Nov 8;3(11):e0002551. doi: 10.1371/journal.pgph.0002551 (PMC10631642; doi:10.1371/journal.pgph.0002551)
Supplement: S2 Table — (DOCX) [file pgph.0002551.s008.docx]

**S2 Table.** **Cost analysis results for management of moderate wasting in infants and children <60 months of age**

| **Author, year** | **Country, WHO region** | **Target population** | **Treatment arms** | **Setting, level of care/treatment setting** | **Cost perspective** | **Cost per** | | |
| --- | --- | --- | --- | --- | --- | --- | --- | --- |
|  |  |  |  |  |  | **Child treated** | **Child recovered** | **Other** |
| **Initiation of treatment in a community setting** | | | | | | | | |
| Nkonki (2017) [163] | South Africa; Africa | 0-59 months^#^^ | Supplementation food and maternal education | NR; community | Provider |  |  | Total: $5,874,105 |
| Purwestri (2012) [165] | Indonesia; South-East Asia | 6-59 months | Daily distribution of RUF Nias biscuits | Semi-urban; community | Provider (institutional) | $860 SD: $284 - $1,435 | $502 SD: $47 - $958 |  |
|  |  |  | Weekly distribution of RUF Nias biscuits | Rural; community |  | $943 SD: $385 - $1,502 | $467 SD: $23 - $911 |  |
|  |  |  | Daily distribution of RUF Nias biscuits | Semi-urban; community | Societal | $1,380 SD: $426 - $2,335 | $822 SD: $75 - $1,568 |  |
|  |  |  | Weekly distribution of RUF Nias biscuits | Rural; community |  | $1,218 SD: $463 - $1,972 | $604 SD: $17 - $1,190 |  |
| Reed (2012b) [167] | Kenya; Africa | 6-59 months | Supplementation (mostly Plumpy-Nut^®^, a commercial product of Nutriset | Arid, semi-arid lands and Urban; community | Provider | $201 |  |  |
| Reed (2012c) [166] | Nepal; South-East Asia | 6-59 months | Counselling | NR; community | Provider | $18 |  |  |
| Rogers (2017) [169] | Malawi; Africa | 6-59 months | Control (monthly rations of 1L oil+8kg corn-soy blend (CSB)+social and behaviour change communication (SBCC)) | Rural; home | Societal | $237 |  |  |
|  |  |  | Intervention group 1 (2.6L oil+8 kg CSB+SBCC in bulk+enhanced SBCC) |  |  | $407 |  |  |
|  |  |  | Intervention group 2 (2.6L oil+8 kg CSB+SBCC in 4 X 2kg packages with printed messages+enhanced SBCC) |  |  | $449 |  |  |
| **Initiation of treatment in outpatient settings** | | | | | | | | |
| Isanaka (2019) [158] | Mali; Africa | 6-35 months | Treat with RUSF | Rural; community health centre | Provider | $247 |  |  |
|  |  |  | Treat with CSB++ |  |  | $251 |  |  |
|  |  |  | Treat with MI |  |  | $252 |  |  |
|  |  |  | Treat with LMF |  |  | $277 |  |  |
| Reed (2012a) [168] | Pakistan; Eastern Mediterranean | 6-59 months | Supplementary feeding programme with RUSF, RUTF or fortifies blended foods | Rural & urban; NS | Provider | $118 |  |  |

^No subgroup analysis for those aged <6 months

^#^No subgroup analysis for those aged 6-59 months
