# Supplementary material for: Costs and cost-effectiveness of treatment setting for children with wasting, oedema and growth failure/faltering: A systematic review
Source: PLOS Glob Public Health. 2023 Nov 8;3(11):e0002551. doi: 10.1371/journal.pgph.0002551 (PMC10631642; doi:10.1371/journal.pgph.0002551)
Supplement: S3 Table — (DOCX) [file pgph.0002551.s009.docx]

**S3 Table. Cost-effectiveness analysis results for the management of moderate wasting in infants and children <60 months of age**

| **Author, year** | **Country, WHO region** | **Target population** | **Comparator** | **Setting, level of care/treatment setting** | **Cost perspective** | **Intervention** | **Cost per** | | |
| --- | --- | --- | --- | --- | --- | --- | --- | --- | --- |
|  |  |  |  |  |  |  | **Incremental cost per death averted** | **Incremental cost per DALY averted** | **Other** |
| Isanaka (2019) [158] | Mali; Africa | 6-35 months | No treatment | Rural; community health centre | Provider | Initiation of treatment in outpatient settings | Reference | Reference |  |
|  |  |  | Treat with RUSF |  |  |  | $27,246 | $963 |  |
|  |  |  | Treat with CSB++ |  |  |  | Dominated | Dominated |  |
|  |  |  | Treat with MI |  |  |  | Dominated | Dominated |  |
|  |  |  | Treat with LMF |  |  |  | Dominated | Dominated |  |
| Rogers (2017) [169] | Malawi; Africa | 6-59 months | Monthly rations of 1L oil+8kg corn soy blend (CSB)+social and behaviour change communication (SBCC) | Rural; home | Societal | Initiation of treatment in a community setting |  |  | Reference |
|  |  |  | Intervention group 1 (2.6L oil+8 kg CSB+SBCC in bulk + enhanced SBCC) |  |  |  |  |  | $249 per additional caregiver meeting or exceeding target |
|  |  |  | Intervention group 2 (2.6L oil+8 kg CSB+SBCC in 4 X 2kg packages with printed messages + enhanced SBCC) |  |  |  |  |  | $396 per additional caregiver meeting or exceeding target |
