# Supplementary material for: Costs and cost-effectiveness of treatment setting for children with wasting, oedema and growth failure/faltering: A systematic review
Source: PLOS Glob Public Health. 2023 Nov 8;3(11):e0002551. doi: 10.1371/journal.pgph.0002551 (PMC10631642; doi:10.1371/journal.pgph.0002551)
Supplement: S4 Table — (DOCX) [file pgph.0002551.s010.docx]

**S4 Table.** **Cost analysis results for the management of** **severe wasting and/or bilateral pitting oedema in infants and children <60 months of age**

| **Author, year** | **Country, WHO region** | **Target population** | **Treatment arms** | **Setting, level of care/treatment setting** | **Cost perspective** | **Cost per** | | |
| --- | --- | --- | --- | --- | --- | --- | --- | --- |
|  |  |  |  |  |  | **Child treated** | **Child recovered** | **Other** |
| **Initiation of treatment in a community setting** | | | | | | | | |
| Akram (2016) [146] | Pakistan; Eastern Mediterranean | 6-23 months | Locally produced Indigenous high-density diet (HDD) plus a micronutrient powder ‘Baby Active’ | Rural; home | Provider |  | $126 |  |
| Garg (2018) [156] | India; South-East Asia | 6-59 months | RUTF-C (government) | Urban; home | Provider | $1,779 |  | $13  per child covered |
|  |  |  | RUTF-L (government) |  |  | $1,812 |  |  |
|  |  |  | A-HPF (government) |  |  | $2,047 |  |  |
|  |  |  | RUTF-C (research setting) |  |  | $7,618 |  | $54  per child covered |
|  |  |  | RUTF-L (research setting) |  |  | $7,685 |  |  |
|  |  |  | A-HPF (research setting) |  |  | $7,987 |  |  |
| Isanaka (2017) [160] | Niger; Africa | 6-59 months | N/A | Rural; community | Provider | $202 |  |  |
| Nkonki (2017) [163] | South Africa; Africa | Severe wasting; 0-59 months | Supplementation food and maternal education | NR; community | Provider |  |  | Total: $13,205,874 |
| Puette (2013) [164] | Bangladesh; South-East Asia | 6-36 months | Community treatment | Rural; community | Societal | $671 | $732 |  |
| Rogers (2018) [171] | Mali; Africa | 6-59 months | CHW-delivered care | Rural; community | Societal | $691 | $733 |  |
|  |  |  |  |  | Provider | $662 | $703 |  |
| Rogers (2019) [170] | Pakistan; Eastern Mediterranean | 6-59 months | Intervention (government employed lady health workers complemented by NGO delivered outpatient care) | NR; community | Provider (institutions) | $799 | $1,051 |  |
|  |  |  |  |  | Provider (government) | $206 | $270 |  |
|  |  |  |  |  | Community | $64 | $84 |  |
| Tekeste (2012) [172] | Ethiopia; Africa | 6-59 months | Community based therapeutic care | Rural; community | Societal | $757 | $817 |  |
| Wilunda (2021) [174] | Tanzania; Africa | 6-59 months | Community health workers -delivered care | Rural; home | Provider | $426 | $470 |  |
| **Initiation of treatment in a community/inpatient/outpatient settings** | | | | | | | | |
| Isanaka (2017) [160] | Niger; Africa | 6-59 months | N/A | Rural; hospital & community | Provider | $399 |  |  |
| UNICEF (2012) [176] | Chad; Africa | 0-59 months | Community based therapeutic care  Outpatient care | Rural; Kindergarten  Rural; Health Centres | Provider | $505 |  |  |
| Wilford (2011) [173] | Malawi; Africa | <60 months | CMAM + health services | NR; hospital/PHC/community | Provider | $445 |  |  |
|  |  |  | CMAM - health services |  |  | $44 |  |  |
| **Initiation of treatment in outpatient settings** | | | | | | | | |
| Ali (2017) [147] | Nigeria; Africa | <60 months | Outpatient therapeutic centre | NR; Outpatient therapeutic center | Provider | $67 |  |  |
|  |  |  | No programme implementation | NR; No programme implementation |  | $8 |  |  |
|  |  |  | Outpatient therapeutic centre | NR; Outpatient therapeutic center | Societal | $76 |  |  |
|  |  |  | No programme implementation | NR; No programme implementation |  | $10 |  |  |
| Ashraf (2019) [148] | Bangladesh; South-East Asia | 2-59 months | Outpatient at a day clinic | Urban; day clinic | Societal | $529 |  |  |
|  |  |  |  |  | Provider | $474 |  |  |
|  |  |  |  |  | Household | $55 |  |  |
| Ashworth (1997) [149] | Bangladesh; South-East Asia | 12-60 months | Day care | Urban; day care | Provider (Institutional) |  | $329 |  |
|  |  |  |  |  | Parental |  | $25 |  |
| Bachmann (2009) [150] | Zambia; Africa | <60 months | Outpatient at primary health care centre | Urban; PHC | Provider | $78 |  |  |
|  |  |  | Do nothing |  |  | $0 |  |  |
| Bai (1972) [151] | India; South-East Asia | <60 months | Outpatient at rural health centre | Rural; health centre | NR | $6,365 |  |  |
| Fotso (2019) [154] | Ethiopia; Africa | <60 months | Outpatient with CMAM surge approach (designed to support better planning and response to periodic  caseload surges) | NR; Health centres and health posts | Societal |  | $1,129 |  |
|  |  |  | Outpatient with standard CMAM services |  |  |  | $438 |  |
| Frankel (2015) [155] | Nigeria; Africa | NR months | Outpatients | NR; PHC | Societal |  | $500 |  |
| IRC (2016) [157] | Kenya; Africa | <60 months | Outpatient at a health care centre | NR; PHC | Provider | $395 |  |  |
|  | Kenya; Africa |  |  |  |  | $335 |  |  |
|  | Mali; Africa |  |  |  |  | $788 |  |  |
|  | Mali; Africa |  |  |  |  | $854 |  |  |
|  | Mali; Africa |  |  |  |  | $291 |  |  |
|  | Niger; Africa |  |  |  |  | $237 |  |  |
|  | Yemen; Eastern Mediterranean |  |  |  |  | $3,374^+^ |  |  |
|  | Yemen; Eastern Mediterranean |  |  |  |  | $2,412^+^ |  |  |
| Isanaka (2019) [158] | Mali; Africa | 6-35 months | Treat SAM only | Rural; community health centre | Provider | $103 |  |  |
| N'Diaye (2020) [162] | Burkina Faso; Africa | 6-59 months | Standard RUTF dose | Urban & non-urban; PHC | Provider (institutional) | $204 |  |  |
|  |  |  |  |  | Societal | $246 |  |  |
|  |  |  | Reduced RUTF dose |  | Provider (institutional) | $162 |  |  |
|  |  |  |  |  | Societal | $205 |  |  |
|  |  |  |  |  |  |  |  |  |
| Reed (2012a) [168] | Pakistan; Eastern Mediterranean | 6-59 months | Outpatient treatment programme | Rural & urban; outpatient centres | Provider | $1,267 |  |  |
| Reed (2012b) [167] | Kenya; Africa | 6-59 months | Outpatient treatment | Arid, semi-arid lands and Urban; PHC | Provider | $334 |  |  |
| Reed (2012c) [166] | Nepal; South-East Asia | 6-59 months | Outpatient treatment | NR; PHC | Provider | $188 |  |  |
| Rogers (2018) [171] | Mali; Africa | 6-59 months | Outpatient facility-based care | Rural; PHC | Societal | $1,252 | $1,419 |  |
|  |  |  |  |  | Provider | $1,208 | $1,369 |  |
| Rogers (2019) [170] | Pakistan; Eastern Mediterranean | 6-59 months | Control (NGO delivered outpatient care) | NR; PHC | Provider (institutions) | $790 | $952 |  |
|  |  |  |  |  | Provider (government) | $199 | $239 |  |
|  |  |  |  |  | Community | $117 | $141 |  |
| Tekeste (2012) [172] | Ethiopia; Africa | 6-59 months | Facility based care | Rural; therapeutic feeding centre | Societal | $1,597 | $1,796 |  |
| Wilunda (2021) [174] | Tanzania; Africa | 6-59 months | Outpatient care | Rural; PHC | Provider | $470 | $626 |  |
| **Transfer from outpatient to community treatment** | | | | | | | | |
| Ashworth (1997) [149] | Bangladesh; South-East Asia | 12-60 months | Domiciliary care | Urban; household care | Provider (Institutional) |  | $163 |  |
|  |  |  |  |  | Parental |  | $52 |  |
| **Referral to treatment in an inpatient setting** | | | | | | | | |
| Masiiwa (2013) [161] | Zimbabwe; Africa | 0-59 months | 0 | Urban; hospital | Household |  |  | $84*  per household |
| Puette (2013) [164] | Bangladesh; South-East Asia | 6-36 months | Standard of care (inpatient) | Rural; Upazila health complex | Societal | $5,465 | $37,204 |  |
| Reed (2012a) [168] | Pakistan; Eastern Mediterranean | 6-59 months | Stabilization centre | Rural & urban; hospital | Provider | $714 |  |  |
| Reed (2012b) [167] | Kenya; Africa | 6-59 months | Stabilisation centre | Arid, semi-arid lands and Urban; Hospital | Provider | $298 |  |  |

*Costs are as reported in the paper due to hyperinflation in the country and inability to convert costs to meaningful 2020 USD

^+^ The 2013 PPP was used for Yemen in the absence of a more recent figure
