# Supplementary material for: Costs and cost-effectiveness of treatment setting for children with wasting, oedema and growth failure/faltering: A systematic review
Source: PLOS Glob Public Health. 2023 Nov 8;3(11):e0002551. doi: 10.1371/journal.pgph.0002551 (PMC10631642; doi:10.1371/journal.pgph.0002551)
Supplement: S5 Table — (DOCX) [file pgph.0002551.s011.docx]

**S5 Table. Cost-effectiveness analysis results for the management of severe wasting and/or bilateral pitting oedema in infants and children <60 months of age**

| **Author, year** | **Country, WHO region** | **Target population** | **Comparator** | **Setting, level of care/treatment setting** | **Cost perspective** | **Intervention** | **Cost per** | | |
| --- | --- | --- | --- | --- | --- | --- | --- | --- | --- |
|  |  |  |  |  |  |  | **Death averted** | **DALY averted** | **Other** |
| Ali (2017) [147] | Nigeria; Africa | <60 months | Outpatient therapeutic centre | NR; Outpatient therapeutic centre | Provider | Initiation of treatment in outpatient settings |  | $145 | $5,376 per life saved |
|  |  |  | No programme implementation | NR; No programme implementation |  |  |  | Reference | Reference |
|  |  |  | Outpatient therapeutic centre | NR; Outpatient therapeutic centre | Societal |  |  | $161 | $5,951 per life saved |
|  |  |  | No programme implementation | NR; No programme implementation |  |  |  | Reference | Reference |
| Bachmann (2009) [150] | Zambia; Africa | <60 months | Outpatient at primary health care centre | Urban; PHC | Provider | Initiation of treatment in outpatient settings | $677 95% CI: $228 - $3,903 | $20 95% CI: $6.93 - $118 |  |
|  |  |  | Do nothing |  |  |  | Reference | Reference |  |
| Fotso (2019) [154] | Ethiopia; Africa | <60 months | Outpatient with CMAM surge approach | NR; Health centres and health posts | Societal | Initiation of treatment in outpatient settings | $4,973 95%CI: $3,640.33 - $7,089 | $70 95%CI: $53 - $91 |  |
|  |  |  | Outpatient with standard CMAM services |  |  |  | $2,480 95%CI: $1,891.32 - $3,781 | $35 95%CI: $27 - $48 |  |
| Frankel (2015) [155] | Nigeria; Africa | NR months | Outpatients | NR; PHC | Societal | Initiation of treatment in outpatient settings | $2,549 | $68 |  |
|  |  |  |  |  |  | Do nothing | Reference | Reference |  |
| Puette (2013) [164] | Bangladesh; South-East Asia | 6-36 months | No treatment (zero cost) | Rural; Upazila health complex | Societal | Referral to treatment in an inpatient setting | Reference | Reference |  |
|  |  |  | Standard of care (inpatient) |  |  |  | $185,787 | $5,465 |  |
|  |  |  | No treatment (zero cost) | Rural; community |  | Initiation of treatment in community settings | Reference | Reference |  |
|  |  |  | Community treatment |  |  |  | $3,534 | $106 |  |
| Rogers (2019) [170] | Pakistan; Eastern Mediterranean | 6-59 months | NGO delivered outpatient care | NR; PHC | Provider (institutions) | Initiation of treatment in outpatient settings |  |  | Incremental cost per additional child recovered: $1,483 |
|  |  |  | Government employed lady health workers complemented by NGO delivered outpatient care | NR; community |  | Initiation of treatment in a community setting |  |  | Reference |
| Wilford (2011) [173] | Malawi; Africa | <60 months | CMAM + health services | NR; hospital/PHC/community | Provider | Initiation of treatment in a community/inpatient/outpatient settings |  | $110 |  |
|  |  |  | CMAM - health services |  |  |  |  | Reference |  |
| Wilunda (2021) [174] | Tanzania; Africa | 6-59 months | Outpatient care | Rural; PHC | Provider | Initiation of treatment in outpatient settings |  |  | Reference |
|  |  |  | Community health workers -delivered care | Rural; community | Provider | Initiation of treatment in a community setting |  |  | $391 per additional child treated |
|  |  |  |  |  |  |  |  |  | $381 per additional child cured |
