# Supplementary material for: Costs and cost-effectiveness of treatment setting for children with wasting, oedema and growth failure/faltering: A systematic review
Source: PLOS Glob Public Health. 2023 Nov 8;3(11):e0002551. doi: 10.1371/journal.pgph.0002551 (PMC10631642; doi:10.1371/journal.pgph.0002551)
Supplement: S6 Table — (DOCX) [file pgph.0002551.s012.docx]

**S6 Table. Cost analysis results for the management of moderate wasting and severe wasting and/or bilateral pitting oedema together in infants and children <60 months of age**

| **Author, year** | **Country, WHO region** | **Target population** | **Treatment arms** | **Setting, level of care/treatment setting** | **Cost perspective** | **Cost per** | | |
| --- | --- | --- | --- | --- | --- | --- | --- | --- |
|  |  |  |  |  |  | **Child treated** | **Child recovered** | **Other** |
| **Transfer from inpatient to outpatient treatment** | | | | | | | | |
| Chapko (1994) [153] | Niger; Africa | MAM and SAM; 5-28 months | Transfer from inpatient to outpatient care | Urban; ambulatory rehabilitation centre | Provider | $98 |  |  |
|  |  |  | Transfer from inpatient to another inpatient facility | Urban; hospital |  | $217 |  |  |
| **Initiation of treatment in outpatient settings** | | | | | | | | |
| Bailey (2020) [152] | Kenya, South Sudan; Africa | MAM and SAM; 6-59 months | Standard protocol: different protocols for SAM & MAM | Rural & urban; PHC | Societal |  | $2,855 |  |
|  |  |  | Combined protocol: same protocol for SAM and MAM |  |  |  | $2,518 |  |
| Gomez (1983) [175] | Chile; Americas | SAM and MAM; 0-23 months | Outpatient treatment | Urban; Outpatient | Societal |  | $11* |  |
|  |  | SAM and MAM; 24 -71 months |  |  |  |  | $5.89* |  |
|  |  | SAM and MAM; 0 -71 months |  |  | Household | $5.50^+^ |  |  |
| Initiation of treatment in a community setting | | | | | | | | |
| Gomez (1983) [175] | Chile; Americas | SAM and MAM; 0-23 months | Community treatment | Urban; Kindergarten | Societal |  | $21* |  |
|  |  | SAM and MAM; 24 -71 months |  |  |  |  | $15* |  |
|  |  | SAM and MAM; 0 -71 months |  |  | Household | $1.49^+^ |  |  |

*cost per child recovered per day

^+^cost per child treated per day
