# Supplementary material for: Costs and cost-effectiveness of treatment setting for children with wasting, oedema and growth failure/faltering: A systematic review
Source: PLOS Glob Public Health. 2023 Nov 8;3(11):e0002551. doi: 10.1371/journal.pgph.0002551 (PMC10631642; doi:10.1371/journal.pgph.0002551)
Supplement: Attachment — Submitted filename: Responses to Reviewers.docx [file pgph.0002551.s013.docx]

**Costs and cost-effectiveness of treatment setting for children with wasting, oedema and growth failure/faltering: a systematic review**

**Responses to Editorial and Reviewer comments**

We thank the editorial team and the reviewers for their comments. We have addressed them and they have certainly helped improve our manuscript. Below are the point-by-point responses to each of the comments.

**Editorial comments**

| **Comment** | **Response** |
| --- | --- |
| Please provide separate figure files in .tif or .eps format. | We have now uploaded figure file as .tif files. |
| Please send a completed 'Competing Interests' statement, including any COIs declared by your co-authors. If you have no competing interests to declare, please state "The authors have declared that no competing interests exist". | The ‘competing interests’ statement is included in the submission system |
| Please provide a detailed Financial Disclosure statement. This is published with the article. It must therefore be completed in full sentences and contain the exact wording you wish to be published.  a. State the initials, alongside each funding source, of each author to receive each grant. For example: ""This work was supported by the National Institutes of Health (####### to AM; ###### to CJ) and the National Science Foundation (###### to AM).""  b. State what role the funders took in the study. If the funders had no role in your study, please state: “The funders had no role in study design, data collection and analysis, decision to publish, or preparation of the manuscript.” | The financial disclosure statement is included in the submission system |
| Copyright issues with Fig 2 | Fig 2 have been removed as information on the countries within which the studies were conducted is also provided in Table 3. |
| We do not publish any copyright or trademark symbols that usually accompany proprietary names, eg (R), (C), or TM (e.g. next to drug or reagent names). Please remove all instances of trademark/copyright symbols throughout the text, including ® on page 8. | Copyright or trademark symbols have been removed, including ® on page 8. |
| We have noticed that you have uploaded Supporting Information files, but you have not included a list of legends. Please add a full list of legends for your Supporting Information files after the references list. | A full list of legends for Supporting Information files is now provided after the references list. |

**Reviewer comments**

| **Reviewer Number** | **Comment** | **Response** |
| --- | --- | --- |
| 1 | I appreciate the authors for the tremendous work in completing the review. I am glad that the care giver perspective and the societal perspective are taken into consideration. The review fulfilled the reporting guidelines appropriately. | We thank the reviewer for their positive comment |
| 2 | Overall, the article is good in reporting the effectiveness of treatment settings for children with wasting, oedema, and growth failure/faltering in terms of both cost and outcomes. | We thank the reviewer for their positive comment |
| 2 | 1. In table 1, the exclusion criteria mentioned are just the opposite to the statements mentioned in inclusion criteria which can’t be so and need to be re-framed | We have removed the exclusions that were opposite to the inclusion criteria in Table 1 |
| 2 | 2. Line number 215, 396, 552 to be checked for typo | We thank the reviewer for identifying the typos. We have now amended the lines as follows:  Line 216 in the clean version (previously line 215): *“We revised our review according* ***to*** *the feedback provided where necessary.”*  Line 396: “…*involved a team comprising a doctor,* ***a*** *lay health supervisor,* ***a*** *lady health worker and* ***a*** *project supervisor…”*  Line 553 in the clean version (previously line 552): *“Studie****s*** *identified for this group reported the cost and cost-effectiveness analysis for…”* |
| 2 | 3. Figure 1 reference to 115 records exclusion in search results | We followed the recommended reporting standards by including the references for the 115 records that were excluded in the main text under ‘Search results’ **(page 13, lines 221-225 in the clean version)**, and using the PRISMA flow diagram to provide a snapshot/summary of what is in the text. We have also provided information on each excluded study in Supplementary material 5. We believe that this is adequate. |
